# Quantum nonlinearity for optical neural computing

**DOI:** 10.1038/s41467-026-77202-y

**Published:** 2026-08-27

**Authors:** Qingyi Zhou, Jungmin Kim, Yutian Tao, Guoming Huang, Ming Zhou, Zewei Shao, Zongfu Yu

**Affiliations:** 1 Department of Electrical and Computer Engineering, University of Wisconsin-Madison, Madison, WI USA; 2 The Computer Sciences Department, University of Wisconsin-Madison, Madison, WI USA; 3 Department of Electrical Engineering, Stanford University, Stanford, CA USA

**Keywords:** Optical physics, Optical physics

## Abstract

The rapid scaling of deep neural networks comes at the cost of unsustainable power consumption. While optical neural networks offer an alternative, their capabilities remain constrained by the lack of efficient optical nonlinearities. To address this, we propose an optical neural computing architecture by embedding quantum emitters in inverse-designed nanophotonic structures. Due to their saturability, quantum emitters exhibit exceptionally strong nonlinearity compared with conventional materials. Using physics-aware training, we numerically demonstrate that the proposed architecture can solve complex tasks, including nonlinear classification and reinforcement learning, within all-optical neural networks. To enable fair comparison across different platforms, we introduce a framework that quantitatively links nonlinearity to a network’s expressive power. Analysis shows that our quantum activation operates at nW/*μ*m^2^ intensity, which is seven orders of magnitude below the nonlinearity threshold of conventional optical materials. Looking ahead to large language models, we estimate the nonlinearity-limited optical power, which scales sublinearly with model size. Our results indicate that quantum nanophotonics may provide a route toward sustainable AI inference.

## Abstract (web summary)

Optical neural networks could cut the energy cost of AI, yet weak optical nonlinearity limits their capability. Here, the authors propose activation units by embedding a quantum emitter in an inverse-designed photonic structure, numerically demonstrating all-optical deep learning at nanowatt-scale intensities.

## Introduction

In the past decade, the rapid advancement of deep learning has profoundly transformed science and technology. Deep neural networks have achieved state-of-the-art performance across diverse fields, ranging from computer vision^1^ and game-playing^2^ to protein design^3^ and language processing^4^. Such progress has been driven by the continuous scaling of the model size. However, this scaling trend imposes an energy cost toward unsustainable levels^5^. A growing effort has been directed towards finding alternative computing paradigms. In particular, following the pioneering work of Shen et al.^6^, optical neural networks (ONNs) have emerged as a promising candidate^7^, inspired by the vision that a passive optical device can implement linear transformation with high speed^8^ and low energy cost^9^. Specifically, recent works have demonstrated that linear optical matrix operations can be performed with sub-photon energy consumption^10^. Despite these advantages, linear operations are insufficient for deep learning. The expressive power of ONN is severely limited by the lack of efficient optical nonlinearity^9,11^. In conventional materials, optical nonlinearities are often perturbative^12^. As a result, existing all-optical nonlinear activation units demand high optical power and large footprint^13–17^, making it difficult to scale up. This nonlinearity bottleneck has remained a long-standing challenge for the optical computing community. Existing works that rely on hybrid opto-electronic architectures^6,18–20^ suffer from additional latency and substantial system complexity due to frequent optical-electrical-optical (O/E/O) conversions. More recently, structural nonlinearity schemes have been proposed^21–23^, in which the input is encoded not in the optical field but in tunable parameters of a linear structure. However, the connection between such systems and standard deep learning models is often unclear.

To address the nonlinearity bottleneck, we first point out that nonlinear optical phenomena are not intrinsically restricted to high intensities. A single quantum emitter (including an atom, quantum dot, or color center) can be saturated by the absorption of a few photons per lifetime, leading to extremely strong optical nonlinearity^24^. There have been both theoretical proposals^25,26^ and experimental demonstrations^27,28^ of using quantum emitter media as activation units, underscoring their potential for realizing strong optical nonlinearities. However, it remains unclear what quantitative benefit such quantum nonlinearity offers for ONNs. Furthermore, to fully utilize the nonlinear functionality of quantum emitters, it is essential to enhance the interaction between light and quantum emitters, which can be achieved using properly designed nanophotonic structures. Recent progress in quantum nanophotonics has enabled deterministic integration of individual emitters with on-chip photonic structures^29–33^. Therefore, we believe it is the right time to address the above-mentioned questions and systematically investigate whether quantum technologies can provide the strong nonlinearity required by optical neural networks.

In this work, we present a theoretical proposal for a low-power optical neural network architecture that exploits the strong nonlinearity of individual quantum emitters. Specifically, we introduce a quantum-enhanced activation unit by embedding quantum emitters into adjoint-optimized nanophotonic structures^34^. A strong nonlinear response can be achieved at an intensity level of nW/μm^2^. With full-wave simulations, we verify the performance on nonlinear classification task as well as reinforcement learning tasks, demonstrating functionality beyond linear models. To enable a fair comparison across different physical nonlinearities, we develop a theoretical framework that quantifies the expressive power of an arbitrary activation unit. Unlike existing theoretical analyses that focus on purely linear optics^35–38^ or restricted classes of unitary transformations^39^, our framework directly links a nonlinear input-output response to the depth-wise growth of ONN expressivity. This allows us to translate a targeted expressive power into the required light intensity, enabling a quantitative assessment of ONN’s energy efficiency. Applying this framework, we show that conventional platforms based on the Kerr effect or saturable absorption would require prohibitively high intensities to match the digital baseline. In contrast, the proposed quantum-enhanced activation reduces the required intensity by seven orders of magnitude. Finally, we look into the future by estimating the nonlinearity-limited optical power for optical large language models (LLMs). Our analysis shows that this lower bound, set by the nonlinear activation alone, remains at the watt level for the proposed scheme and scales sublinearly with model size. Taken together, our results indicate that large-scale optical neural computing, powered by quantum-enhanced nonlinearities, could reduce the energy footprint of AI inference, providing a path beyond the limits of electronic hardware.

## Results

### Overcoming the nonlinearity bottleneck with quantum activations

**Fig. 1:**
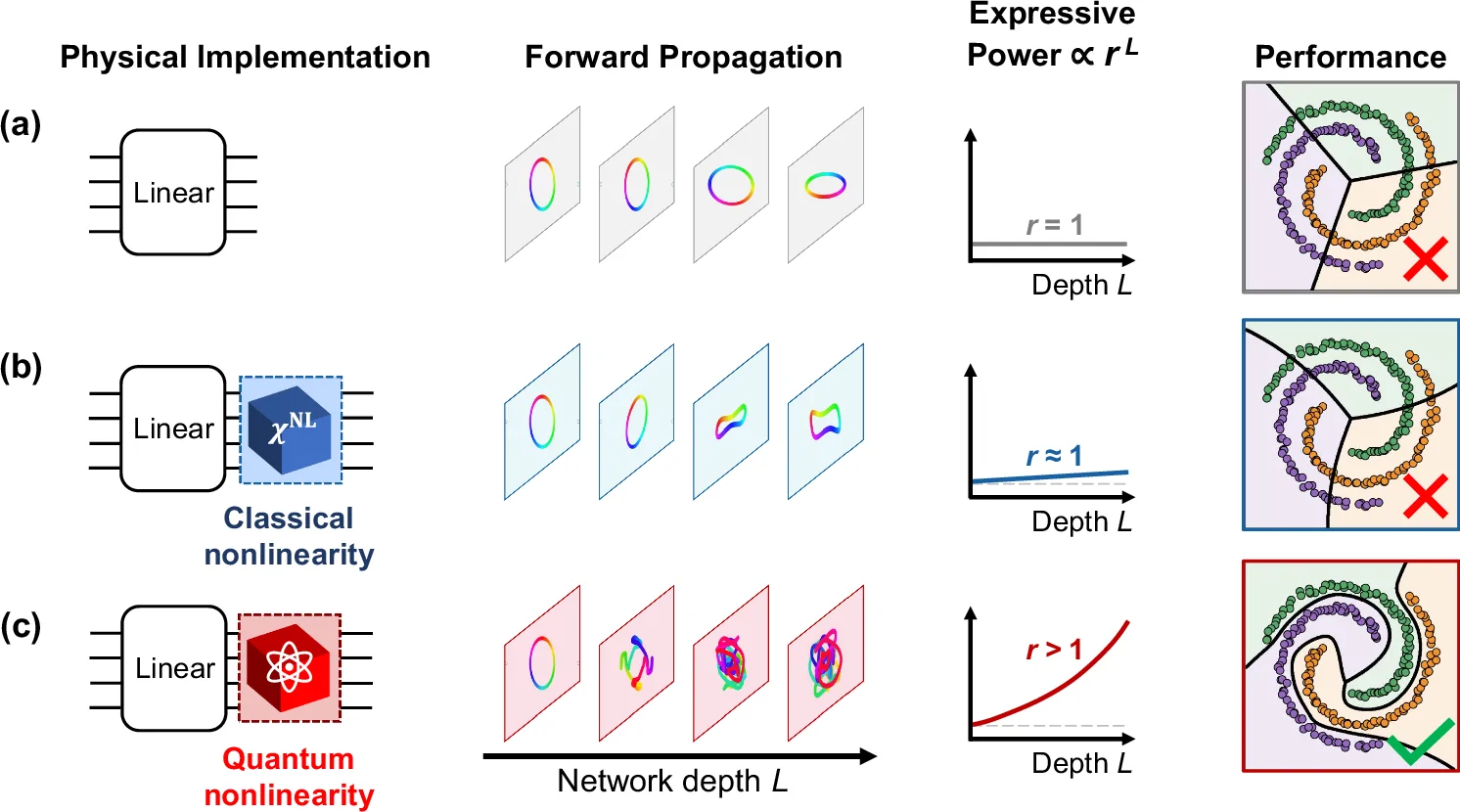
Comparison of ONN architectures with different nonlinearities. **a** A linear ONN, whose expressive power remains constant regardless of depth *L*. **b** ONN with classical nonlinearity. With weak nonlinearity, the expressive power increases slightly with depth, yet is not enough for complex tasks. **c** ONN with quantum nonlinearity. The expressive power  ∝ *r*^*L*^ grows exponentially with *r* > 1, and is able to handle complex tasks.

We consider a generic multi-layer ONN architecture. Each layer consists of a linear transformation **y** = **W**^(*i*)^**x** + **b**^(*i*)^, comprising a weight matrix **W**^(*i*)^ and a bias **b**^(*i*)^, followed by a nonlinear activation *f*( ⋅ ), mirroring the architecture of a typical multi-layer perceptron (MLP). In machine learning theory, it is well established that the expressive power of a deep neural network grows exponentially with its depth^40–42^. A purely linear network cannot enjoy this benefit since a composition of linear transformations is still linear. As a result, the overall expressive power of an ONN is limited by its nonlinear activation units. We follow the framework developed in refs. ^41,42^ and introduce a metric for quantifying expressive power. As illustrated in Fig. 1, a closed trajectory of data points serves as the input. The total curvature *K* of this trajectory provides a robust measure of curve complexity and is monitored as the curve propagates through successive layers. Intuitively, the total curvature measures the degree of folding applied to the data manifold, a capability that is fundamentally impossible with linear transformations. As shown in Fig. 1(a), for a purely linear network, the total curvature remains constant. In contrast, in the presence of nonlinear activations, the curvature increases by a growth factor *r* > 1 after each layer, leading to an exponential growth ~*r*^*L*^ with depth *L*^41,42^. The growth factor *r* is therefore used as a quantitative measure of expressive power (see Supplementary Note 10 for details).

Conventional optical materials possess small nonlinear susceptibilities^12^. At realistic light intensities, the response is only weakly nonlinear, which yields a growth factor *r* ≈ 1. The expressive power, as shown in Fig. 1(b), shows little increase with depth. Existing all-optical activation units typically require mW/*μ*m^2^ laser intensities, together with large footprints^13,14,16,43,44^ (see Supplementary Note 1 for a summary of representative designs obtained from literature). Such requirements are incompatible with large-scale ONNs. On the other hand, it has long been recognized that low-dimensional systems exhibit much stronger optical nonlinearities than bulk media^45–47^, owing to enhanced oscillator strength under quantum confinement^48^. In particular, zero-dimensional quantum emitters behave as two-level systems (TLSs) that can be saturated by the absorption of only a few photons per lifetime. This leads to extremely strong nonlinear scattering responses, which have been observed in various waveguide- and cavity-QED platforms^49–53^. These observations suggest that emitter-based nonlinearities could be leveraged to overcome the nonlinearity bottleneck in ONNs (Fig. 1(c)).

### Device design and verification of nonlinear classification

**Fig. 2:**
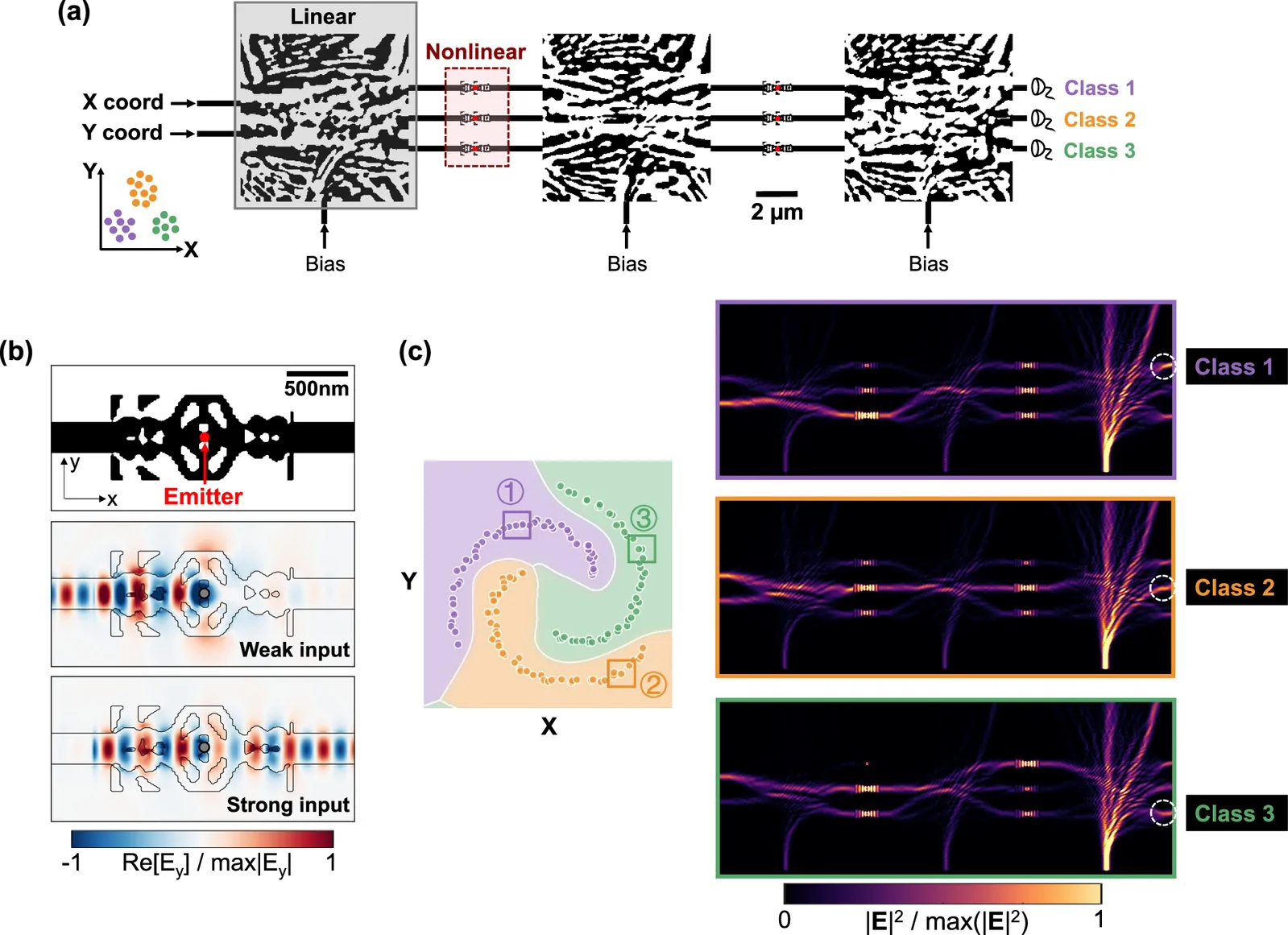
Physics-aware training and verification on nonlinear classification. **a** All-optical neural network design, constructed by stacking nonlinear activation units between linear blocks, which are also designed through adjoint optimization. The 2D coordinates are encoded as input from the left ports, while a separate port at the bottom provides a constant optical bias. The detected intensities are interpreted as classification results. **b** The proposed quantum nonlinear activation unit. A single SiV^−^ color center is embedded in an inverse-designed GaP-on-diamond structure. Simulated ${{\mathrm{Re}}}\,[{E}_{y}]$ field distributions (obtained via 3D nonlinear FDFD) illustrate the transition from resonant scattering to saturation, resulting in a nonlinear response at low intensity. **c** Performance verification. Classification results for the spiral dataset are obtained via nonlinear FDFD simulations. The ∣*E*∣^2^ intensity distributions of 3 representative inputs (marked with square boxes) are visualized. Different intensity distributions at the output port correspond to different predictions.

Motivated by the above considerations, we propose an all-optical activation unit, consisting of a single quantum emitter embedded inside an inverse-designed GaP-on-diamond nanophotonic structure (design region 1.5 × 0.7 μm^2^), as shown in Fig. 2(a). We utilized adjoint optimization to design a nanophotonic interface that maximizes light-matter interaction and minimizes loss (see Supplementary Note 4 for design details). The emitter is modeled as a TLS with a field-driven dipole moment ${d}_{y}\propto \frac{\Omega /{\Gamma }_{0}}{1+2{(\Omega /{\Gamma }_{0})}^{2}}$, where the Rabi frequency *Ω* is set by the local electric field^54,55^ and *Γ*_0_ = 2*π* × 94 MHz is the spontaneous emission rate (parameters are obtained from SiV^−^ color centers, assuming lifetime-limited linewidth; see Supplementary Note 2 for details). The electric field distributions for two different input intensities are shown in Fig. 2(b), both obtained using full-wave three-dimensional nonlinear finite-difference frequency-domain (FDFD) simulations (see Supplementary Note 3 for details). The nonlinear activation unit is constructed based on a realistic GaP-on-diamond platform (200 nm patterned GaP on a 160 nm diamond film, with an SiO_2_ substrate). The passive linear weight blocks are designed based on the variational effective-index method^56,57^. Note that the specific implementation of linear blocks is not central to this work and could be realized with other schemes such as Mach–Zehnder interferometers. The device is engineered to operate in two distinct regimes: in the weak-field limit, the emitter acts as a linear scatterer, while in the strong-field limit, it becomes nearly transparent. In this two-port geometry, the emitter is configured to induce a change in transmission coefficient ∣*Δt*∣ = 1 via interference, which results in a strong nonlinearity. Using *N* > 1 emitters could in principle achieve a larger transmission change of ∣*Δt*∣ = 2 (see Supplementary Note 4 for the analysis). From the simulated input-output curve, we extract an effective nonlinear activation function with an ultra-low intensity threshold. To evaluate the expressive power of the obtained activation function, we compute its growth factor *r*(*I*), which reaches *r* ≈ 1.14 at intensity *I* = 0.24 nW/μm^2^, exceeding the digital baseline. In contrast, conventional silicon- and graphene-based nonlinearities remain near *r* = 1 under similar operating conditions (see Supplementary Note 11 and Supplementary Fig. 22 for details). We have also analyzed the effect of low quantum efficiency and demonstrate that our method remains robust even when the TLS’ quantum efficiency drops to 60% (see Supplementary Note 5 for details). We also characterize the robustness of the activation unit against two different non-idealities: lateral position randomness and spectral disorder (see Supplementary Note 14 for details). These results confirm our key physical intuition: by utilizing saturable quantum emitters embedded in nanophotonic structures, strong optical nonlinearity can be realized at ultra-low optical power.

To demonstrate the practical advantage of the proposed activation, we benchmark our system on nonlinear classification task that is challenging for models that are weakly nonlinear. We adopt a physics-aware training approach to design our ONN in a physically consistent manner. We first characterize the nonlinear activation unit using nonlinear FDFD simulations to obtain its input-output transmission curve (see Supplementary Fig. 8), which is then used as the activation function in a PyTorch-based ONN model. The linear weight matrices are represented as complex transmission matrices subject to energy-preserving constraints, ensuring that they can be implemented by passive structures. With this differentiable model, the ONN is trained in the digital domain via backpropagation. After training, we map the trained network to concrete photonic structures in a modular fashion. For each layer, the trained complex weight matrix is interpreted as a target transmission matrix between input and output ports. We then run a separate adjoint-based optimization to realize a compact block that implements this target matrix with high fidelity (see Supplementary Notes 4 and 8 for quantitative metrics). The nonlinear layers are implemented by inserting the quantum activation units between these inverse-designed linear blocks, as illustrated in Fig. 2(a). By dissecting the network into multiple modules that can be designed individually, this approach avoids heavy full-wave simulations of the entire network during training (the training procedure is explained in Supplementary Note 7). We verify the final design using full-wave nonlinear FDFD simulations. The classification result for a three-class spiral dataset is shown in Fig. 2(c). The corresponding light intensities for three representative input points are also visualized, revealing how the network steers optical energy toward different output regions associated with different classes. We provide more examples in Supplementary Note 6 (performance on MNIST and FashionMNIST) and Supplementary Note 8 (performance on nonlinear regression task). These results confirm that the physics-aware training yields physically realizable ONNs. Within realistic optical intensities well below 1 mW/μm^2^, conventional materials cannot provide sufficient expressive power. In stark contrast, quantum-enhanced nonlinearity can function below 1 nW/μm^2^, enabling the system to solve complex tasks.

### Generalizing to intelligent optical agents: reinforcement learning

Having established that quantum-enhanced activations enable nonlinear classification, we next ask whether the same photonic building blocks can support more complex tasks. Reinforcement learning (RL), which has achieved impressive results on game-playing benchmarks^2,58^ and robotics^59^, provides a natural testbed for our purpose. Note that RL based on photonic spiking network has recently been demonstrated on control benchmarks^60^. To demonstrate the generality of our approach, we evaluate it on two distinct tasks: a discrete game-playing task (Atari Pong) and a continuous control task (HalfCheetah), both provided by the Gymnasium library^61^.

**Fig. 3:**
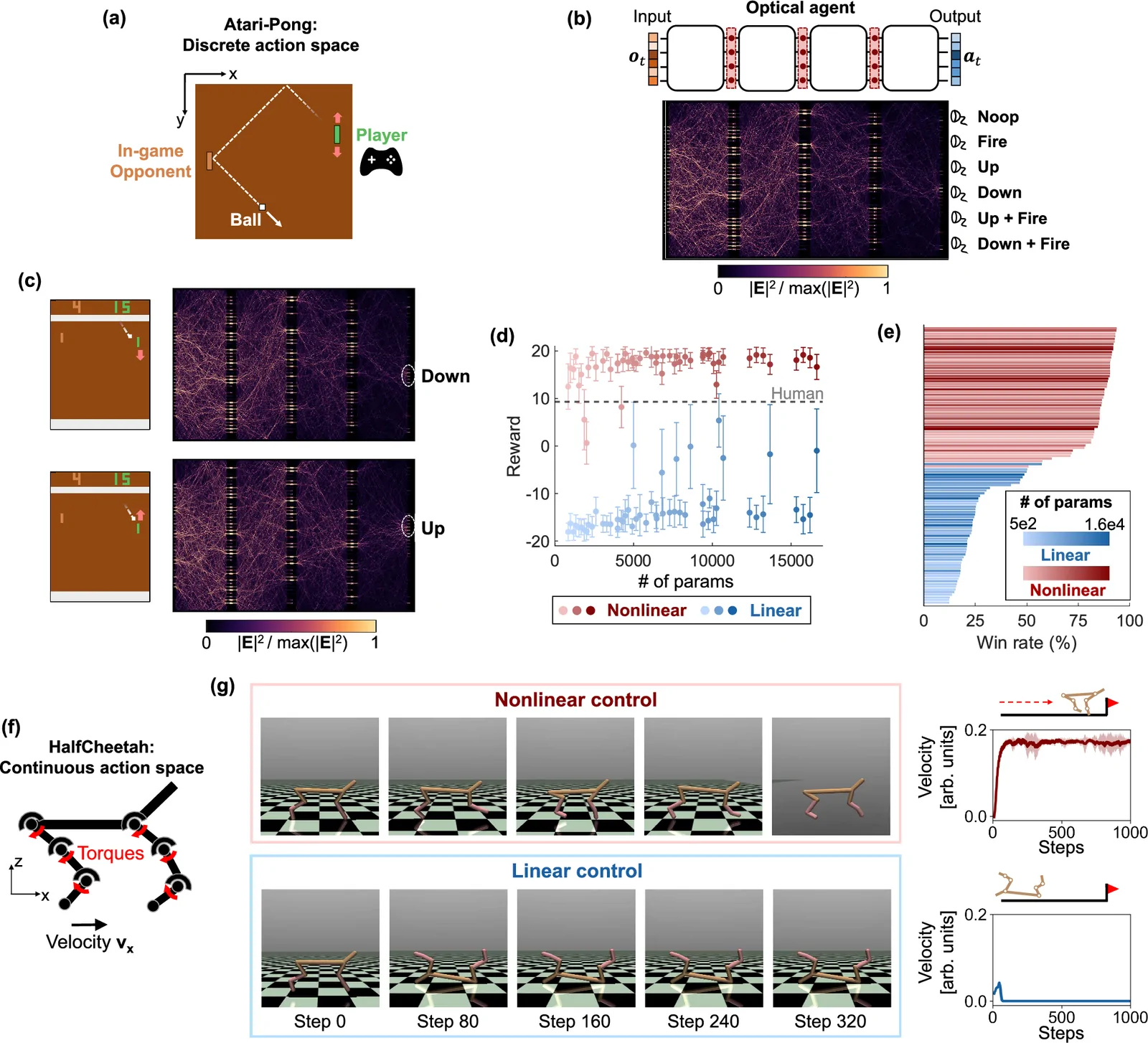
Generalizing to reinforcement learning tasks. **a** Schematic illustration of the Pong environment. The player controls the green paddle and tries to block the ball (white) to win score. **b** The structure of our optical agent, which functions as a policy network. At each time step *t*, historical game frames are encoded into the input **o**_*t*_. The network outputs the logits for six discrete actions. **c** Visualization of the learned policy. Light intensity distributions of two key frames are displayed. **d** Final reward versus model size. Linear models (blue) saturate at a low performance ceiling with high variance. In contrast, nonlinear models (red) converge to near-perfect play very quickly. Error bars denote standard deviations obtained over 30 episodes. The human-level performance (reward = 9.3)^2^ is visualized using gray dashed line. **e** Performance ranking. Best achieved rewards for 104 trained models (52 linear, 52 nonlinear) are sorted. Nonlinear models consistently outperform linear models. **f** Schematic illustration of the HalfCheetah control task. **g** Snapshots obtained during testing. The nonlinear ONN runs stably, while the linear ONN falls down. The insets plot the corresponding velocity curves, averaged over 10 episodes. The shaded areas visualize the standard deviations.

The Atari Pong environment is illustrated schematically in Fig. 3(a). In Pong, an agent controls the right paddle against the in-game opponent. An episode ends when a player reaches 21 points, and the reward is defined as the final score difference. As shown in Fig. 3(b), the ONN acts as a policy network: at each time step *t*, a stack of *F* recent frames is encoded into an observation **o**_*t*_, which serves as the input (see Supplementary Fig. 17 for details). The output intensities (divided into six regions) are interpreted as logits for six discrete actions. An action *a*_*t*_ is sampled from this distribution and sent back to the environment, which then advances to the next time step. The policy parameters are trained using a standard proximal policy optimization (PPO) algorithm^62^, based on the same physics-aware framework described above (see Supplementary Note 9 for details). We note that while the nonlinear activation mechanism has been verified with 3D simulation in Fig. 2, the present Atari Pong demonstration relies on 2D simulations due to limited computing resources. The optical intensity distributions for two representative game frames are shown in Fig. 3(c). Different spatial configurations of the ball and paddles lead to distinct activation patterns and different intensity hotspots at the output ports. We then systematically benchmark the performance of linear versus nonlinear ONNs. We train 104 models, sweeping across network width *H*, number of hidden layers *L*, and the number of input frames *F*. All models are trained for an identical number of iterations (see Supplementary Note 9 for details). The results are summarized in Fig. 3(d), where the final reward is plotted against the number of parameters. Fig. 3(e) further ranks all the trained models based on their final rewards. Linear models saturate at a low performance ceiling regardless of model size and exhibit high variance, indicating that the learned strategies cannot win reliably. In contrast, ONNs equipped with quantum activations achieve much higher rewards as they scale up, converging to near-perfect play.

We further evaluate our ONN on the MuJoCo HalfCheetah control benchmark, as illustrated in Fig. 3(f). With a continuous action space, HalfCheetah is much more challenging than Pong. At each time step, the optical agent receives a 17-dimensional observation (joint positions and velocities, see Supplementary Fig. 18) and outputs a 6-dimensional action vector that specifies torques applied at the six hinge joints. We adopt a similar ONN backbone as in Pong, with one key difference: the output uses balanced detection to support negative action values (see Supplementary Note 9 for details). Training is performed using the standard soft actor-critic (SAC) algorithm^63^. The corresponding snapshots collected during testing are shown in Fig. 3(g). With quantum activation, the ONN learns to run smoothly, whereas a linear ONN fails to acquire a viable control policy and falls down early in the episode. The insets in Fig. 3(g) plot the averaged velocity *v*_*x*_ over 10 episodes, highlighting the stability enabled by optical nonlinearity. The above results confirm that strong nonlinearity is essential for enabling complex capabilities in deep ONNs.

### Nonlinearity-limited power requirements for large-scale networks

Having established the importance of strong nonlinearity at the device level, we now examine how the choice of nonlinearity constrains the optical power of large-scale systems. We consider only the optical power needed to drive the nonlinear activations, and ask what this nonlinearity-limited power would be for present-day LLMs. As a quantitative baseline, we note that in standard digital MLPs, common activation functions typically increase the total curvature by *r*_digital_ ≃ 1.045 ~ 1.095 per layer (see Supplementary Note 10 for details). We therefore ask: for a given physical nonlinearity, what is the minimum optical intensity ${I}_{\min }$ required to match this digital baseline? Using our established framework, we compute the expressive power *r*(*I*) for three representative platforms: Kerr nonlinearity in a 50 μm long silicon waveguide^64,65^, saturable absorption in stacked graphene layers^45,66^ (15 nm total thickness), and our proposed quantum activation unit (see Supplementary Note 11 for details). We find that maintaining the target expressive power in silicon requires intensities exceeding 72.6 W/μm^2^, while graphene requires approximately 0.02 W/μm^2^. In contrast, the quantum activation unit achieves the same baseline at merely  ~ 0.24 nW/μm^2^. This represents an efficiency improvement of roughly 8.3 × 10^7^ times relative to graphene and 3.0 × 10^11^ times relative to silicon. This quantitative comparison reveals the inadequacy of conventional nonlinear materials and shows that the quantum nonlinearity proposed here can overcome this limitation.

Given these intensity thresholds, we next estimate the nonlinearity-limited optical power for LLM-scale models. For a standard decoder-only transformer architecture with context length *L*_seq_, embedding dimension *d*_model_, and *L* transformer layers^4^, we estimate the optical input dimension per layer as 3*L*_seq_*d*_model_, accounting for the parallel projection of query, key, and value matrices. Assuming each optical neuron occupies an effective cross-sectional area *A* ≈ 0.1*μ*m^2^, corresponding to an on-chip waveguide mode,^6^ and is driven at ${I}_{\min }$, the nonlinearity-limited optical power is estimated as

1 $$P\approx {I}_{\min }\,A\cdot (3{L}_{{{{\rm{seq}}}}}{d}_{{{{\rm{model}}}}})\cdot L.$$

**Fig. 4:**
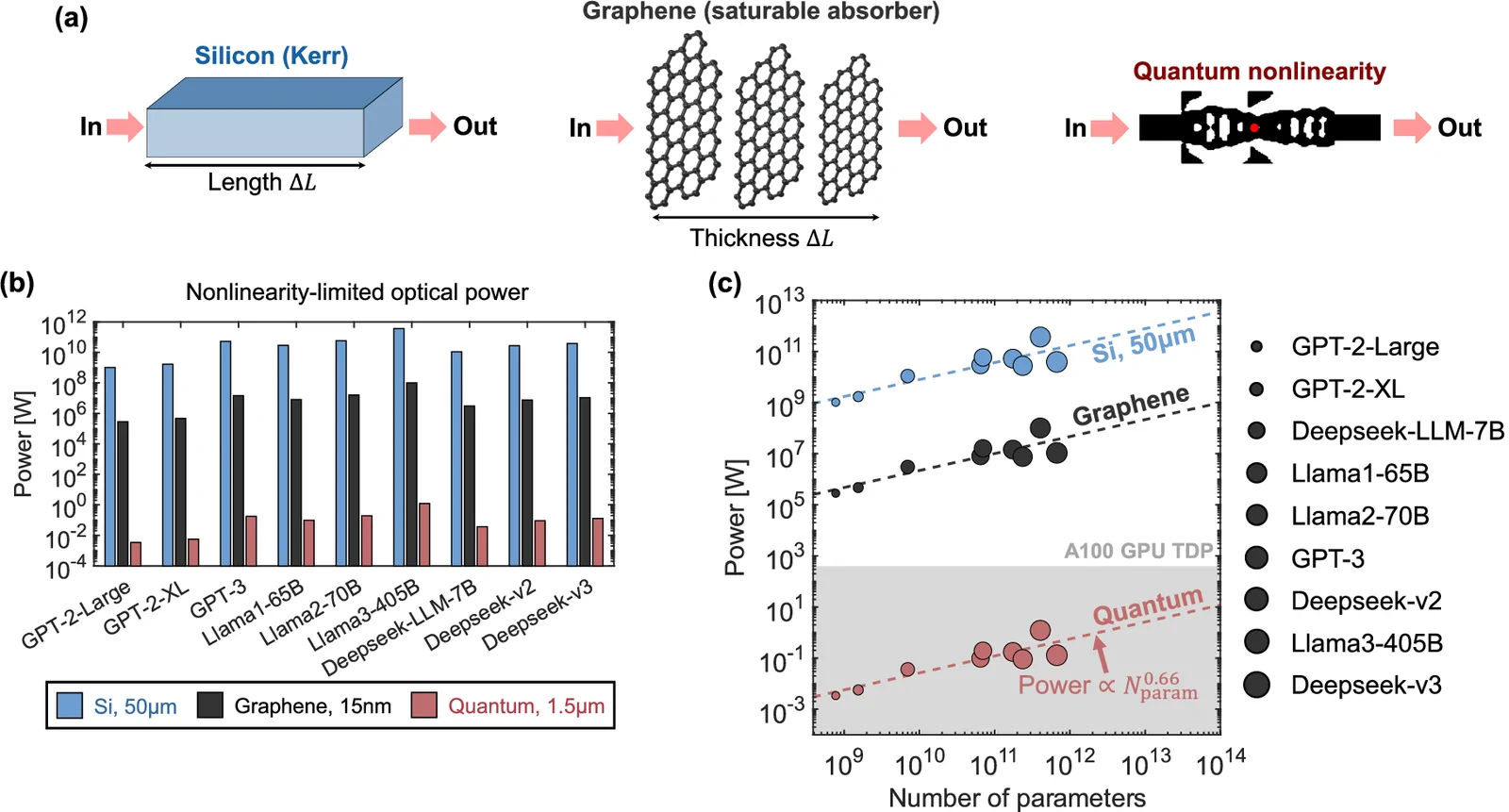
Scalability of nonlinearity-limited power requirements. **a** Schematic illustration of nonlinear platforms: conventional bulk material (silicon, 50 μm length), 2D saturable absorber (stacked graphene, 15 nm thickness), and the proposed quantum activation unit. **b** Histogram showing the estimated nonlinearity-limited optical power of optical LLMs. Conventional materials demand prohibitive power levels, while the proposed scheme is not restricted by nonlinearity. **c** Estimated nonlinearity-limited optical power versus model size *N*_param_. The shaded area marks the thermal design power of a single NVIDIA A100 GPU (≈400 W), shown only as a reference. ONNs follow a sublinear scaling $P\propto {N}_{\,{\mbox{param}}\,}^{0.66}$, as shown by the dashed lines, indicating that optical computing has a growing advantage as models scale up.

This value should be understood as a lower bound set by the nonlinear activation alone and does not represent system-level power consumption. Using the architectural parameters of representative LLMs ranging from GPT-2 to DeepSeek-V3^67–74^ (see Supplementary Note 12 for details), we evaluate Eq. (1) and summarize the results in Fig. 4(b). When using conventional nonlinearities based on silicon or graphene, the required optical power quickly reaches prohibitive levels (exceeding 10^8^ W for the largest models). However, the proposed quantum architecture keeps this nonlinearity-limited power below 1.2W across all investigated models. Finally, we analyze how this power requirement scales with model size. Fig. 4(c) plots the estimated optical power against the total number of trainable parameters, *N*_param_. We also indicate the thermal design power of a single high-end GPU (NVIDIA A100,  ≈ 400W) as a shaded area. We include this only as a rough reference for scale. We do not use it to claim that an optical system would consume less total power than a GPU. For ONNs the data points follow a sublinear scaling law, $P\propto {N}_{\,{\mbox{param}}\,}^{0.66}$. This behavior stems from the geometric nature of the network: the parameter count grows with the volume of the network ($\sim L\cdot {d}_{\,{\mbox{model}}\,}^{2}$), whereas the required optical power scales with the number of inputs ( ~ *L* ⋅ *d*_model_ ⋅ *L*_seq_). Such a distinction leads to a scaling exponent smaller than one, consistent with known results^75^. We would like to point out that the dimensionality of current integrated photonic circuits^76,77^ remains far below LLM scale, so a near-term large-scale optical LLM is more plausibly realized on free-space platforms (e.g., diffractive networks). The same sublinear scaling applies to such platforms, since the exponent comes from the network geometry and is platform-independent. In contrast, the power consumption of electronic processors typically scales linearly with *N*_param_. The nonlinearity-limited optical power therefore scales more favorably with model size, although translating this into a system-level energy advantage would require addressing the additional overheads. Overall, these results suggest that quantum nonlinearity can substantially relax the optical-power bottleneck that conventional nonlinear materials impose at large scale. By shifting to quantum nonlinearities, it should be possible to construct optical deep learning models within a feasible power budget.

## Discussion

In summary, we have presented a theoretical framework to address the nonlinearity bottleneck in optical computing. At the device level, by integrating quantum emitters with inverse-designed nanophotonic structures, strong nonlinearity can be realized at intensities below 1 nW/μm^2^. This enables complex functionalities ranging from nonlinear classification to reinforcement learning, presenting a clear performance gap over linear ONNs. Moreover, we have developed a general theoretical framework to quantify the expressive power of arbitrary nonlinear physical systems, which in turn allows us to determine the light intensity requirements. At the scale of large language models, we estimate the nonlinearity-limited optical power and find that it stays at the watt level, with a favorable sublinear scaling in model size. Together, these results suggest that the lack of nonlinearity is not a fundamental limit, but rather a challenge that could be overcome with quantum technologies.

Despite these advances, transforming our theoretical proposal into large-scale hardware is still facing several practical challenges. A key trade-off exists between intensity threshold and operation bandwidth: the high sensitivity is inherently related to the emitter’s long radiative lifetime. For a bare SiV^−^ center, the intrinsic response bandwidth lies in the sub-GHz range, below the 10–50 GHz modulation rates typically used in optical computing. We point out that this limit is not fundamental, since the response speed can be increased through Purcell enhancement in optimized photonic structures^78^. Our inverse-designed activation units already exhibit an emergent Purcell factor *F*_*P*_ ≈ 2.74 (Supplementary Note 13), and Purcell-enhanced linewidth as large as 2*π* × 4.6 GHz has been demonstrated in photonic crystal cavity^79^, offering a realistic route to GHz-scale operation. Another challenge is the inhomogeneity of solid-state emitters. Since the activation requires each emitter to be resonant with the optical signal, the inhomogeneous broadening of transition frequencies becomes an obstacle as the system scales up. To tackle this issue, solutions such as DC Stark tuning have been demonstrated to tune the resonance of individual emitters^80^, which provides a route to align each emitter independently. Regarding integration, while deterministic placement of emitters remains difficult, recent advances in fabrication techniques offer promising solutions for large-scale integration^32,81,82^. Finally, solid-state quantum emitters often require cryogenic operation to suppress dephasing and to approach lifetime-limited linewidths^83,84^, which introduces an additional power overhead for cooling.

Looking forward, this work shows that in order to unlock the full potential of optical computing, we should exploit the strong nonlinearity provided by quantum emitters rather than conventional bulk materials. By combining inverse-designed nanophotonics with modern deep learning theory, we provide a path toward low-power optical neural computing, in which the lack of strong nonlinearity is no longer the limiting factor. Realizing this vision will ultimately pave the way toward sustainable, next-generation artificial intelligence.

## Methods

### Nonlinear electromagnetic simulations

We model the quantum emitter as an ideal two-level system with ground state $\left\vert g\right\rangle$ and excited state $\left\vert e\right\rangle$. The emitter has a transition frequency *ω*_0_ and a transition dipole moment **d**_0_. In this work we neglect pure dephasing. When the emitter is driven by a monochromatic field, the field-driven dipole moment at steady state can be derived as

2 $${{{\bf{d}}}}=\frac{\Omega \left(-\Delta+i{\Gamma }_{0}/2\right)}{{\Delta }^{2}+{\left({\Gamma }_{0}/2\right)}^{2}+{\Omega }^{2}/2}{{{{\bf{d}}}}}_{0},$$

where *Ω* = *d*_0_*E*_inc_/*ℏ* is the Rabi frequency, *Δ* = *ω*_d_ − *ω*_0_ is the detuning, and *Γ*_0_ is the radiative decay rate. The device simulations are performed under resonant excitation with *Δ* = 0 (analysis regarding the robustness to spectral disorder can be found in Supplementary Note 14). The dipole moment leads to a current source **J**(**r**) = − *iω***d** ⋅ *δ*(**r** − **r**_0_). This current source, which depends nonlinearly on the local electric field, is included in a three-dimensional finite-difference frequency-domain model. Specifically, after spatial discretization, Maxwell’s equations take the form

3 $${{{\bf{F}}}}({{{\bf{x}}}})\equiv {{{\bf{A}}}}{{{\bf{x}}}}-{{{{\bf{b}}}}}_{0}-{{{{\bf{b}}}}}_{{{{\rm{TLS}}}}}({{{\bf{x}}}})={{{\bf{0}}}},$$

where **x** represents the electric field distribution, **A** is the discretized Maxwell operator, **b**_0_ is the external source, and **b**_TLS_ corresponds to the dipole source. The above nonlinear equation can be solved using the Newton–Raphson method. At each iteration, the equation is linearized around the current solution, and the resulting Jacobian system is solved for the field update. Details of the derivation, including the treatment of the dipole’s primary radiation and the full Jacobian matrix, are provided in Supplementary Notes 2 and 3.

### Platform and inverse design

The nonlinear activation unit is based on a single SiV^−^ color center. At cryogenic temperature, we focus on its zero-phonon line at *λ*_0_ = 737.134 nm. The platform consists of a 200-nm-thick patterned GaP layer on a 160-nm-thick unetched diamond film and a SiO_2_ substrate. The refractive indices of GaP, diamond, and SiO_2_ are 3.22, 2.40, and 1.45, respectively. The input light enters through a GaP strip waveguide (260 nm wide and 200 nm thick) via its fundamental TE mode. A single SiV^−^ center is placed at the center of the design region, 30 nm below the diamond surface, with its dipole oriented along the *y* direction.

We optimize the in-plane GaP pattern within a 1.5 × 0.7 μm^2^ design region using the adjoint method. The optimization runs for 500 iterations using Adam. A conic filter (radius 100 nm) is also applied to control the minimum feature size. The objective function and material parameterization are explained in Supplementary Note 4. We also design the passive linear blocks on the same platform to realize the weight matrices. We use a 2.5D variational effective-index method to reduce the computational cost. Details of the effective-index model and adjoint optimization are provided in Supplementary Note 4.

### Physics-aware neural-network training

We use the response curve obtained from nonlinear FDFD simulations as the activation function in the neural network. We first train the neural network in the digital domain using PyTorch. All complex weight matrices are constrained to be isometric, so that they can be implemented using passive photonic structures. After training, each matrix is treated as a target transmission matrix and translated into a linear optical block through adjoint optimization. The optimized linear blocks are then concatenated with the nonlinear activation units to obtain the final structure.

For the reinforcement learning tasks, the Atari Pong task is trained using proximal policy optimization (PPO), while the HalfCheetah task is trained using soft actor–critic (SAC). For each task, the linear and nonlinear models use the same network architecture and training settings, with the activation disabled in the linear baseline. Further details on network training and evaluation are provided in Supplementary Notes 7 and 9.

### Quantification of expressive power

In order to evaluate the expressive power of a given neural network, we follow the approach of Raghu et al.^42^ and use a circular trajectory as the network input,

4 $${{{\bf{x}}}}(\theta )=\sqrt{{N}_{{{{\rm{dim}}}}}}\left[{{{\bf{u}}}}\cos (\theta )+{{{\bf{v}}}}\sin (\theta )\right],\qquad \theta \in [0,2\pi ],$$

where *N*_dim_ is the input dimension, and **u** and **v** are random orthonormal unit vectors. Specifically, we propagate this trajectory through successive network layers and calculate its total curvature *K* after each layer. A linear transformation does not increase the total curvature, whereas a nonlinear activation progressively deforms the trajectory, which can lead to an exponential increase in total curvature with network depth. The growth factor *r* can be extracted by fitting the layer-dependent curvature to *K* ∝ *r*^*L*^, where *L* denotes the network depth. Thus, a larger *r* indicates stronger expressive power. For optical activations, we repeat this procedure at different input intensities to obtain *r* as a function of intensity, *r*(*I*). Details related to the trajectory sampling, network dimensions, and curve fitting are provided in Supplementary Notes 10 and 11.

## Supplementary information

Supplementary Information

Transparent Peer Review file

## Data Availability

The data supporting the findings of this study are publicly available together with the associated code in the GitHub repository at https://github.com/zhouqingyi616/quantum-nonlinearity-for-optical-neural-computing. The same files are permanently archived on Zenodo at https://doi.org/10.5281/zenodo.21762053.

## Code availability

The custom code used to generate and analyze the results reported in this study is publicly available together with the supporting data in the GitHub repository at https://github.com/zhouqingyi616/quantum-nonlinearity-for-optical-neural-computing. The same files are permanently archived on Zenodo at https://doi.org/10.5281/zenodo.21762053.
