# Supplementary material for: Quantum nonlinearity for optical neural computing
Source: Nat Commun. 2026 Aug 27;17:10241. doi: 10.1038/s41467-026-77202-y (PMC13616904; doi:10.1038/s41467-026-77202-y)
Supplement: Supplementary file 1 [file 41467_2026_77202_MOESM1_ESM.pdf]

Supplementary information for
"Quantum Nonlinearity for Optical Neural Computing"

Qingyi Zhou†,[1,*] Jungmin Kim,[1,*] Yutian Tao,[2,*] Guoming Huang,[1] Ming Zhou,[3] Zewei Shao,[1] and Zongfu Yu[1]

[1] *Department of Electrical and Computer Engineering, University of Wisconsin-Madison, Madison, WI 53706, USA*

[2] *The Computer Sciences Department, University of Wisconsin-Madison, Madison, WI 53706, USA*

[3] *Department of Electrical Engineering, Stanford University, Stanford, CA 94305, USA*

(Dated: August 11, 2026)

CONTENTS

Supplementary Note 1. Comparison with existing literature 3

Supplementary Note 2. Dipole moment of two-level system 5

Supplementary Note 3. Nonlinear FDFD simulation 7
A. FDFD with nonlinear dipole 7
B. Discretized nonlinear equation 7
C. Newton-Raphson solver 8
D. Benchmarking nonlinear FDFD 9

Supplementary Note 4. Adjoint-based optimization 10
A. Why optimization is required 10
B. Adjoint method 10
C. Material platform of the activation unit 11
D. Optimization setup 12
E. Understanding $N_{\mathrm{TLS}} = 2$ with coupled-mode theory 12
F. Final result 14
1. 3D activation unit 14
2. 2D activation unit 14
G. Inverse design of linear blocks 16

Supplementary Note 5. Effect of low quantum efficiency 18

Supplementary Note 6. Performance on supervised learning tasks 19
A. MNIST 19
B. FashionMNIST 19

Supplementary Note 7. Physics-aware training procedure 20

Supplementary Note 8. Nonlinear regression task 21

Supplementary Note 9. Reinforcement learning tasks: details 23
A. Atari Pong 23
B. HalfCheetah 24

Supplementary Note 10. Theory: quantifying expressive power 26

Supplementary Note 11. Expressive power of optical nonlinearities 28
A. Optical nonlinearity of conventional materials 28
1. Kerr effect of silicon 28

* These authors contributed equally to this work.
† Corresponding author: qzhou75@wisc.edu

2. Saturable absorption based on graphene 28
B. Quantifying the expressive power 28
C. Comparison with quantum-enhanced activation 30

Supplementary Note 12. Power requirements of optical LLMs 31
A. Architecture of modern LLMs 31
B. Estimating the nonlinearity-limited optical power 31

Supplementary Note 13. Purcell factor of the activation unit 33

Supplementary Note 14. Robustness against emitter non-idealities 34
A. Robustness to emitter position randomness 34
B. Robustness to spectral disorder 34

Supplementary References 35

SUPPLEMENTARY NOTE 1. COMPARISON WITH EXISTING LITERATURE

In this part, we compare the proposed quantum-enhanced nonlinear activation unit with other activation units proposed in existing literature. We first show in Supplementary Fig. 1 a high-level comparison between different types of implementations. As shown in the left column, most existing optical neural network architectures choose to carry out activation function in electrical domain. In that sense, optical-electrical-optical (O/E/O) conversion is required for every single layer, making the system complicated. On the other hand, all-optical neural network architectures do not require O/E/O conversion for the intermediate layers. However, when relying on conventional materials, these ONNs often suffer from intrinsically small nonlinear susceptibilities. To compensate for such weak nonlinearity, high laser power and large footprint are often required for all-optical NNs. In order to address these challenges, we propose to construct an activation unit based on the strong nonlinearity of single quantum emitter. As shown in the right column of Supplementary Fig. 1, the proposed scheme reaches strong nonlinearity at low light intensity ($\sim$ nW/μm^2), and has a very compact footprint ($\sim$ 5μm) at the same time.

	Electrical nonlinearity	Optical nonlinearity	
Device schematic	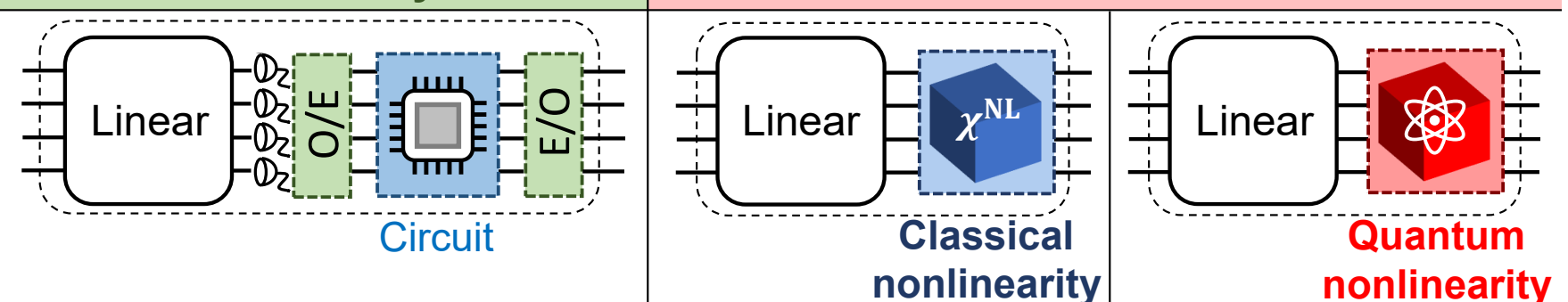		
Nonlinearity	Strong	Weak	Strong
Threshold	~mW/μm²	~mW/μm²	~nW/μm²
Footprint	Large: ~100μm	Large: ~100μm	Small: ~5μm
System complexity	High (O/E: PD+TIA; E/O: Driver)	Low (transduction-free)	Low (transduction-free)

Supplementary Figure 1. **Comparison of ONN architectures with different mechanisms for nonlinear activations.** Carrying out nonlinear operations on electrical signal (the left column) requires frequent O/E/O conversions, which leads to extra overhead. Optical nonlinearities based on conventional materials (the middle column) are typically weak, thus requiring high laser power and large footprint. Our proposed quantum-enhanced nonlinearity (the right column) can function well under low laser power, with a much smaller footprint.

Next, we display the comparison results by comparing our proposed activation unit with existing literature. We focus on two metrics: the footprint, defined as the 2D area occupied by the activation unit; the nonlinearity threshold, defined as the required light intensity to observe obvious nonlinear effect. The results are shown in Supplementary Fig. 2 as a scatter plot. Red circles correspond to all-optical implementations [1–13], while blue squares correspond to opto-electronic implementations [14–17]. Unfilled circles indicate that the corresponding papers are theoretical proposals and include no experimental results. As a reference, we also visualize the nonlinearity thresholds for seven different materials, namely:
(1) silicon, with $\chi^{(3)} = 2.45 \times 10^{-19}$ m^2/V^2 [18];
(2) MoS_2, with $\chi^{(2)} = 10^{-7}$ m/V [19];
(3) tellurene, with $\chi^{(2)} = 3.26 \times 10^{-9}$ m/V [20];
(4) graphene, with intensity threshold 5.3×10^{-3} W/μm^2 [21, 22].
(5) As_2S_3, with $n_2 = 3 \times 10^{-18}$ m^2/W [23];
(6) AlGaAs, with $n_2 = 2.6 \times 10^{-17}$ m^2/W [24];
(7) $LiNbO_3$, with $\chi^{(2)} = 27$ pm/V [25];
The solar irradiance (1360.8 W/m^2) [26] is also marked using the orange dashed line, which is much lower than the nonlinearity thresholds of existing activation units. The threshold of each reference material is obtained by requiring the nonlinear refractive-index change to reach $\Delta n = 0.01$. For a material with third-order nonlinearity, $\Delta n = n_2 I$, so the threshold intensity is $I_{\rm th} = \Delta n / n_2$, with the nonlinear index obtained from the third-order susceptibility through $n_2 = 3\chi^{(3)}/(4\varepsilon_0 c n_0^2)$. For a material with second-order nonlinearity, the field-induced index change is $\Delta n = \chi^{(2)} E$, giving a threshold intensity $I_{\rm th} = \frac{1}{2}\varepsilon_0 c\,(\Delta n/\chi^{(2)})^2$. For the individual works compiled in Supplementary Fig. 2, the operating intensity is estimated from the input condition reported in each paper. Note that this comparison is intended as an order-of-magnitude overview rather than a precisely standardized benchmark. At the same time, our

proposed activation unit shows a threshold that is orders of magnitude lower than all existing implementations.

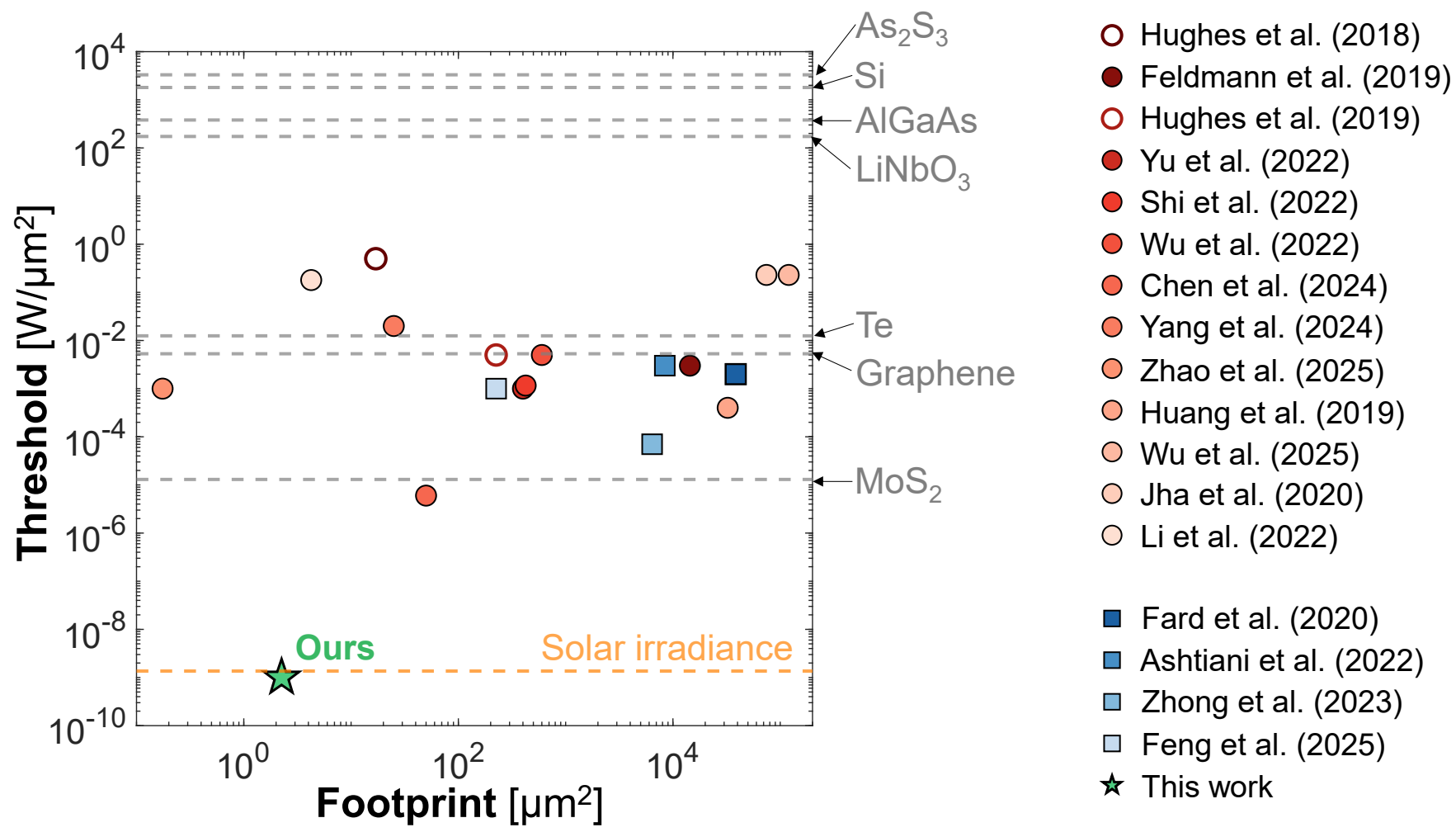

Supplementary Figure 2. **Detailed comparison.** We summarize the performance of different activation units proposed in literature [1–17]. Unfilled markers indicate theoretical proposals. The nonlinearity thresholds of several materials have been visualized using gray dashed lines. Each material threshold corresponds to the input intensity at which the nonlinear index change reaches $\Delta n = 0.01$. The comparison is intended as an order-of-magnitude overview.

SUPPLEMENTARY NOTE 2. DIPOLE MOMENT OF TWO-LEVEL SYSTEM

In this part we derive the dipole moment of a quantum emitter, under the illumination of a monochromatic incident wave. The quantum emitter is modeled as an ideal two-level system (TLS), with ground state $|g\rangle$ and excited state $|e\rangle$. The resonance frequency is denoted as $\omega_0 = \frac{E_e - E_g}{\hbar}$. We denote the dipole moment matrix element as $\mathbf{d}_0 = \langle e|\hat{\mathbf{d}}|g\rangle$, which is assumed to be a real quantity without loss of generality. Such a TLS has radiative decay rate $\Gamma_0 = \frac{d_0^2\omega_0^3}{3\pi\hbar\epsilon_0 c_0^3}$ in free space. The Hamiltonian of such a TLS driven by electromagnetic field can be written as

$$H = H_0 + H_{\rm int} = \hbar\omega_0 \left|e\right\rangle \left\langle e\right| - \mathbf{d}_0 \cdot \mathbf{E}(t) \big(\left|g\right\rangle \left\langle e\right| + \left|e\right\rangle \left\langle g\right|\big). \tag{1}$$

The time-evolution of the density matrix obeys the von-Neumann equation $\frac{d\rho}{dt} = -\frac{i}{\hbar}[H,\, \rho]$. The operators can be resolved in the two-level basis:

$$\rho = \begin{pmatrix} \rho_{\rm gg} & \rho_{\rm ge} \\ \rho_{\rm eg} & \rho_{\rm ee} \end{pmatrix},\ H_0 = \begin{pmatrix} 0 & 0 \\ 0 & \hbar\omega_0 \end{pmatrix},\ H_{\rm int}(t) = \begin{pmatrix} 0 & -\,\mathbf{d}_0\!\cdot\!\mathbf{E}(t) \\ -\,\mathbf{d}_0\!\cdot\!\mathbf{E}(t) & 0 \end{pmatrix}. \tag{2}$$

By calculating the commutators, the above equations give

$$-\frac{i}{\hbar}\,[H_0, \rho] = \begin{pmatrix} 0 & i\,\omega_0\,\rho_{\rm ge} \\ -\,i\,\omega_0\,\rho_{\rm eg} & 0 \end{pmatrix}, \tag{3}$$

$$-\frac{i}{\hbar}\,[H_{\rm int}, \rho] = \frac{i\,(\mathbf{d}_0\!\cdot\!\mathbf{E})}{\hbar} \begin{pmatrix} \rho_{\rm ge} - \rho_{\rm eg} & \rho_{\rm ee} - \rho_{\rm gg} \\ \rho_{\rm gg} - \rho_{\rm ee} & \rho_{\rm eg} - \rho_{\rm ge} \end{pmatrix}. \tag{4}$$

The time-evolution of density matrix ρ thus follows

$$\begin{cases} \dfrac{d\,\rho_{\rm ee}}{dt} = -\Gamma_0\,\rho_{\rm ee} + \dfrac{2\,(\mathbf{d}_0\!\cdot\!\mathbf{E})}{\hbar}\ {\rm Im}\big[\rho_{\rm eg}\big], \\ \dfrac{d\,\rho_{\rm eg}}{dt} = \Big(-\,i\omega_0 - \dfrac{\Gamma_0}{2}\Big)\rho_{\rm eg} + \dfrac{i\,(\mathbf{d}_0\!\cdot\!\mathbf{E})}{\hbar}\big(1 - 2\rho_{\rm ee}\big). \end{cases} \tag{5}$$

Note that the radiative decay rate Γ_0 has been introduced phenomenologically. Next we assume that the polarization of the incident wave is aligned with the dipole moment $\mathbf{d}_0$, so that the inner product can be replaced by $d \cdot E(t)$. The monochromatic incident field oscillates at the laser frequency ω_d, so that its time-profile can be written as $E(t) = E_0\cos(\omega_d t) = \frac{E_0}{2}(e^{i\omega_d t} + e^{-i\omega_d t})$. The corresponding Rabi frequency can be defined as $\Omega = \frac{d_0 E_0}{\hbar}$. We further define the detuning $\Delta = \omega_d - \omega_0$. Transforming into the frame rotating at the laser frequency, where the slowly-varying coherence is $\tilde{\rho}_{eg} = \rho_{\rm eg}e^{i\omega_d t}$, the equations become

$$\begin{cases} \dfrac{d\,\rho_{\rm ee}}{dt} = \Omega\ {\rm Im}\big[\tilde{\rho}_{eg}\big] - \Gamma_0\,\rho_{\rm ee}, \\ \dfrac{d\,\tilde{\rho}_{eg}}{dt} = i\dfrac{\Omega}{2}\big(1 - 2\rho_{\rm ee}\big) + \Big(i\Delta - \dfrac{\Gamma_0}{2}\Big)\tilde{\rho}_{eg}. \end{cases} \tag{6}$$

If we further assume that the laser is on resonance with the emitter, i.e. $\Delta = 0$, the above equations reduce to

$$\begin{cases} \dfrac{d\,\rho_{\rm ee}}{dt} = \Omega\ {\rm Im}\big[\tilde{\rho}_{eg}\big] - \Gamma_0\,\rho_{\rm ee}, \\ \dfrac{d\,\tilde{\rho}_{eg}}{dt} = i\dfrac{\Omega}{2}\big(1 - 2\rho_{\rm ee}\big) - \dfrac{\Gamma_0}{2}\tilde{\rho}_{eg}, \end{cases} \tag{7}$$

The steady state of the above equation can be derived by enforcing the time-derivatives to be zero. The results can be summarized as

$$\rho_{\rm ee}(t \to \infty) = \frac{1}{2}\cdot\frac{\Omega^2}{\Omega^2 + \Gamma_0^2/2}, \tag{8}$$

$$\tilde{\rho}_{eg}(t \to \infty) = \frac{1}{2}\cdot\frac{i\Gamma_0\Omega}{\Omega^2 + \Gamma_0^2/2}. \tag{9}$$

At steady state, the TLS shows a dipole moment $\mathbf{d} = 2\mathbf{d}_0 \cdot \tilde{\rho}_{eg}(t \to \infty) = 2i\mathbf{d}_0 \cdot \frac{\Gamma_0 \Omega}{\Gamma_0^2 + 2\Omega^2}$. The physical intuition behind these equations can be understood by checking two extreme cases: when the incident field is weak ($\Omega \ll \Gamma_0$), the TLS acts like a resonant dipole whose dipole moment $\mathbf{d} \propto \frac{\Omega}{\Gamma_0}$ is proportional to E_0; on the other hand, when the incident field is strong ($\Omega \gg \Gamma_0$), the TLS saturates with $\rho_{\rm ee} \approx \frac{1}{2}$ and the dipole moment vanishes.

The above derivations have assumed that the TLS is ideal. In reality, the presence of non-radiative decay rate $\Gamma_{\rm nrad}$ leads to imperfect quantum efficiency $\eta_Q = \frac{\Gamma_0}{\Gamma_0 + \Gamma_{\rm nrad}} < 1$. By including this non-radiative decay, the time-evolution in Supplementary Equation (5) becomes

$$\begin{cases} \dfrac{d\,\rho_{\rm ee}}{dt} = -(\Gamma_0 + \Gamma_{\rm nrad})\,\rho_{\rm ee} + \dfrac{2\,(\mathbf{d}_0 \cdot \mathbf{E})}{\hbar}\ \mathrm{Im}\big[\rho_{\rm eg}\big], \\ \dfrac{d\,\rho_{\rm eg}}{dt} = \Big(-i\omega_0 - \dfrac{\Gamma_0 + \Gamma_{\rm nrad}}{2}\Big)\rho_{\rm eg} + \dfrac{i\,(\mathbf{d}_0 \cdot \mathbf{E})}{\hbar}\big(1 - 2\rho_{\rm ee}\big). \end{cases} \tag{10}$$

Note that we do not include pure dephasing rate γ_ϕ in this paper. The dipole moment now becomes $\mathbf{d} = 2i\mathbf{d}_0 \cdot \frac{(\Gamma_0 + \Gamma_{\rm nrad}) \cdot \Omega}{(\Gamma_0 + \Gamma_{\rm nrad})^2 + 2\Omega^2}$, which is weaker compared to the ideal case.

SUPPLEMENTARY NOTE 3. NONLINEAR FDFD SIMULATION

In this part, we explain in detail the formulation of our full-wave simulations. Our simulations are implemented by introducing a nonlinear dipole source into the standard finite-difference frequency domain (FDFD) approach.

A. FDFD with nonlinear dipole

We start from Maxwell's equations in frequency domain:

$$\nabla \times \mathbf{H} = \mathbf{J} - i\omega\epsilon_r\epsilon_0\mathbf{E}, \quad \nabla \times \mathbf{E} = i\omega\mu_0\mathbf{H}, \tag{11}$$

where $\mathbf{J}$ denotes the current source, and ϵ_r is the spatial distribution of relative permittivity. By eliminating $\mathbf{H}$ field the above equations can be simplified as

$$-\nabla \times \nabla \times \mathbf{E} + \epsilon_r k_0^2\mathbf{E} = -i\omega\mu_0\mathbf{J}, \tag{12}$$

where $k_0 = \omega/c_0$ denotes the wavenumber. In conventional electromagnetic simulations where the current source $\mathbf{J}$ is given, the $\mathbf{E}$ field can be obtained by treating Supplementary Equation (12) as a linear equation $\mathbf{Ax} = \mathbf{b}$, where

$$\mathbf{A} \sim -\nabla \times \nabla \times +\epsilon_r k_0^2, \quad \mathbf{x} \sim \mathbf{E}, \quad \mathbf{b} \sim -i\omega\mu_0\mathbf{J}. \tag{13}$$

In our case, however, the current source $\mathbf{J}$ is provided by the TLS and is related to the $\mathbf{E}$ field. Specifically, since we are working in frequency domain, the relationship between current density $\mathbf{J}$ and the TLS' dipole moment $\mathbf{d}$ is

$$\mathbf{J}(\mathbf{r}) = -i\omega\mathbf{d} \cdot \delta(\mathbf{r} - \mathbf{r}_0), \tag{14}$$

where $\delta(\mathbf{r} - \mathbf{r}_0)$ is the Dirac δ-function, indicating that the dipole is placed at $\mathbf{r} = \mathbf{r}_0$.

For a single TLS, its dipole moment has already been derived as

$$\mathbf{d} = \frac{\Omega(-\Delta + i\frac{\Gamma_0}{2})}{\Delta^2 + (\frac{\Gamma_0}{2})^2 + \frac{\Omega^2}{2}} \cdot \mathbf{d}_0. \tag{15}$$

When discretized on a uniform grid (spatial resolution Δx), the above equation corresponds to a current source term at location $\mathbf{r}_0$:

$$\mathbf{b}_{\text{TLS}}(\mathbf{r} = \mathbf{r}_0) = -i\omega_0\mu_0 \cdot \frac{-i\omega_0\mathbf{d}}{(\Delta x)^3} = -\frac{\mu_0\omega_0^2}{(\Delta x)^3} \cdot \frac{\Omega(-\Delta + i\frac{\Gamma_0}{2})}{\Delta^2 + (\frac{\Gamma_0}{2})^2 + \frac{\Omega^2}{2}} \cdot \mathbf{d}_0, \tag{16}$$

where we have included $\frac{1}{(\Delta x)^3}$ as a discretization of the Dirac δ-function. Note that $\mathbf{E}^{\text{inc}}(\mathbf{r}_0)$ does not equal to the total field $\mathbf{E}(\mathbf{r}_0)$ since the TLS cannot be driven by its primary radiation field [27, 28]. In the next part we will explain how this can be handled numerically.

B. Discretized nonlinear equation

In our FDFD framework, the simulation domain is discretized into N grid points. The Maxwell's equations can be discretized as $\mathbf{Ax} = \mathbf{b}_0 + \mathbf{b}_{\text{TLS}}$, where $\mathbf{A}$ corresponds to the operator $-\nabla \times \nabla \times +\epsilon_r k_0^2$ and is treated as an $3N \times 3N$ sparse matrix; $\mathbf{x}$ corresponds to $\mathbf{E}$ field distribution and is treated as an $3N \times 1$ vector; the source term $\mathbf{b}_0$ is determined by the incident waves, while the other source term $\mathbf{b}_{\text{TLS}}$ takes into account all TLSs.

For the sake of convenience, we first define a $3N \times 3N$ sparse matrix $\mathbf{M}$, which serves as a "mask" and helps identify the locations of all TLSs:

$$M_{ij} = \begin{cases} 1, & \text{if } i = j \text{ and index } (i - N) \text{ contains a TLS;} \\ 0, & \text{otherwise.} \end{cases} \tag{17}$$

Here we use the index $i - N$ to identify the y component of the electric field, since the dipole moment of our quantum emitter is assumed to align with the y-axis. As we have mentioned, in Supplementary Equation (16) the dipole is driven by $\mathbf{E}^{\text{inc}}(\mathbf{r}_0)$ instead of the total field $\mathbf{E}(\mathbf{r}_0)$, since a field-driven dipole cannot be driven by its own primary

radiation field. Therefore the primary radiation field should be excluded explicitly. Based on the above intuition, we further assume that the detune $\Delta = 0$. By defining the parameter $\alpha_{\text{TLS}} = -\frac{2i\mu_0\omega_0^2 d_0^2}{\Gamma_0\hbar(\Delta x)^3}$, the discretized version of Supplementary Equation (16) can be written as

$$\mathbf{b}_{\text{TLS}} = \alpha_{\text{TLS}}\mathbf{M}\cdot f\left(\mathbf{x} - \beta_{\text{rad}}\mathbf{b}_{\text{TLS}}\right), \tag{18}$$

where the element-wise nonlinear function $f(\cdot)$ is defined as

$$f(x) = \frac{x}{1+2|\frac{d_0 x}{\Gamma_0\hbar}|^2}. \tag{19}$$

The term $\beta_{\text{rad}}\mathbf{b}_{\text{TLS}}$ denotes the primary radiation field that TLSs produce at their own locations. Coefficient β_{rad} is a constant which only depends on the background medium that the TLSs are embedded in. It is determined numerically through a small FDFD simulation, by simulating a point source in homogeneous background and extracting the Green's function at the source location. Based on Supplementary Equation (18), we now proceed to determine the final form of our nonlinear FDFD. Maxwell's equations are discretized as $\mathbf{Ax} = \mathbf{b}_0 + \mathbf{b}_{\text{TLS}}$, which gives $\mathbf{b}_{\text{TLS}} = \mathbf{Ax} - \mathbf{b}_0$. Thus, by eliminating $\mathbf{b}_{\text{TLS}}$ we arrive at a nonlinear equation w.r.t. electric field $\mathbf{x}$:

$$\mathbf{F}(\mathbf{x}) \triangleq \mathbf{Ax} - \mathbf{b}_0 - \alpha_{\text{TLS}}\cdot\mathbf{M}\cdot f\left[\mathbf{x} - \beta_{\text{rad}}(\mathbf{Ax} - \mathbf{b}_0)\right] = \mathbf{0}. \tag{20}$$

The above nonlinear equation $\mathbf{F}(\mathbf{x}) = 0$ is what we aim to solve.

C. Newton-Raphson solver

In order to solve for $\mathbf{F}(\mathbf{x}) = 0$ we apply the Newton-Raphson method [29]. The vanilla Newton-Raphson algorithm can be understood as updating the solution $\mathbf{x}$ in an iterative manner: $\mathbf{x} \to \mathbf{x} - \mathbf{J}(\mathbf{x})^{-1}\mathbf{F}(\mathbf{x})$, where $\mathbf{J}(\mathbf{x}) \triangleq \frac{\partial \mathbf{F}(\mathbf{x})}{\partial \mathbf{x}}$ denotes the Jacobian matrix. In this part we derive the explicit form of $\mathbf{J}(\mathbf{x})$. For the sake of convenience, we define an intermediate variable $\mathbf{s} = \mathbf{x} - \beta_{\text{rad}}(\mathbf{Ax} - \mathbf{b}_0)$. The change of variable leads to

$$\mathbf{J}(\mathbf{x}) = \mathbf{A} - \alpha_{\text{TLS}}\cdot\mathbf{M}\cdot\frac{df(\mathbf{s})}{d\mathbf{s}}\cdot\frac{\partial\mathbf{s}}{\partial\mathbf{x}}. \tag{21}$$

Note that $\frac{\partial\mathbf{s}}{\partial\mathbf{x}} = \mathbb{1} - \beta_{\text{rad}}\mathbf{A}$, where $\mathbb{1}$ denotes the identity matrix. However, since $\mathbf{s}$ is complex, $\frac{df(\mathbf{s})}{d\mathbf{s}}$ should be treated carefully using Wirtinger derivatives [30]. By re-writing $f(\mathbf{s})$ as

$$f(\mathbf{s},\mathbf{s}^*) = \frac{\mathbf{s}}{1+2\left(\frac{d_0}{\Gamma_0\hbar}\right)^2\mathbf{s}^*\odot\mathbf{s}}, \tag{22}$$

the corresponding Wirtinger derivatives can be derived as

$$\frac{\partial f}{\partial\mathbf{s}} = \frac{1}{\left(1+2(\frac{d_0}{\Gamma_0\hbar})^2|\mathbf{s}|^2\right)^2}, \quad \frac{\partial f}{\partial\mathbf{s}^*} = -\frac{2(\frac{d_0}{\Gamma_0\hbar})^2\mathbf{s}^2}{\left(1+2(\frac{d_0}{\Gamma_0\hbar})^2|\mathbf{s}|^2\right)^2}. \tag{23}$$

Therefore, the Jacobian can be calculated as

$$\begin{aligned}\frac{\partial\mathbf{F}}{\partial\mathbf{x}} &= \mathbf{A} - \alpha_{\text{TLS}}\mathbf{M}\cdot\left(\frac{\partial f(\mathbf{s},\mathbf{s}^*)}{\partial\mathbf{s}}\cdot\frac{\partial\mathbf{s}}{\partial\mathbf{x}} + \frac{\partial f(\mathbf{s},\mathbf{s}^*)}{\partial\mathbf{s}^*}\cdot\frac{\partial\mathbf{s}^*}{\partial\mathbf{x}}\right)\\ &= \mathbf{A} - \alpha_{\text{TLS}}\mathbf{M}\cdot\text{diag}\left\{\frac{1}{\left(1+2(\frac{d_0}{\Gamma_0\hbar})^2|\mathbf{s}|^2\right)^2}\right\}\cdot(\mathbb{1} - \beta_{\text{rad}}\mathbf{A}),\end{aligned} \tag{24}$$

$$\begin{aligned}\frac{\partial\mathbf{F}}{\partial\mathbf{x}^*} &= -\alpha_{\text{TLS}}\mathbf{M}\cdot\left(\frac{\partial f(\mathbf{s},\mathbf{s}^*)}{\partial\mathbf{s}}\cdot\frac{\partial\mathbf{s}}{\partial\mathbf{x}^*} + \frac{\partial f(\mathbf{s},\mathbf{s}^*)}{\partial\mathbf{s}^*}\cdot\frac{\partial\mathbf{s}^*}{\partial\mathbf{x}^*}\right)\\ &= -\alpha_{\text{TLS}}\mathbf{M}\cdot\text{diag}\left\{-\frac{2(\frac{d_0}{\Gamma_0\hbar})^2\mathbf{s}^2}{\left(1+2(\frac{d_0}{\Gamma_0\hbar})^2|\mathbf{s}|^2\right)^2}\right\}\cdot(\mathbb{1} - \beta_{\text{rad}}^*\mathbf{A}^*).\end{aligned} \tag{25}$$

Based on the above results, we can finally arrive at the formulation of our Newton-Raphson solver. At each iteration, the solution is updated via $\mathbf{x} \to \mathbf{x} + \Delta\mathbf{x}$, where $\Delta\mathbf{x} = \mathrm{Re}(\Delta\mathbf{x}) + i\,\mathrm{Im}(\Delta\mathbf{x})$ can be solved using the following linear equation:

$$\begin{bmatrix} \mathrm{Re}(\frac{\partial \mathbf{F}}{\partial \mathbf{x}}) + \mathrm{Re}(\frac{\partial \mathbf{F}}{\partial \mathbf{x}^*}) & -\mathrm{Im}(\frac{\partial \mathbf{F}}{\partial \mathbf{x}}) + \mathrm{Im}(\frac{\partial \mathbf{F}}{\partial \mathbf{x}^*}) \\ \mathrm{Im}(\frac{\partial \mathbf{F}}{\partial \mathbf{x}}) + \mathrm{Im}(\frac{\partial \mathbf{F}}{\partial \mathbf{x}^*}) & \mathrm{Re}(\frac{\partial \mathbf{F}}{\partial \mathbf{x}}) - \mathrm{Re}(\frac{\partial \mathbf{F}}{\partial \mathbf{x}^*}) \end{bmatrix} \begin{bmatrix} \mathrm{Re}(\Delta\mathbf{x}) \\ \mathrm{Im}(\Delta\mathbf{x}) \end{bmatrix} = - \begin{bmatrix} \mathrm{Re}(\mathbf{F}(\mathbf{x})) \\ \mathrm{Im}(\mathbf{F}(\mathbf{x})) \end{bmatrix}. \tag{26}$$

D. Benchmarking nonlinear FDFD

We provide a simple example that serves as the benchmark for our nonlinear FDFD solver. Consider a single TLS placed inside vacuum, and a plane-wave serves as the incident wave. It is well-known that the scattering cross section of a resonant dipole cannot exceed σ_0, which is $\sigma_0 = \frac{3\lambda_0^2}{2\pi}$ in 3D space. Here we benchmark using a 2D simulation, where the maximum scattering cross section is $\sigma_0 = \frac{2\lambda_0}{\pi}$. We now consider the fact that this TLS can be saturated when the intensity of the incident wave is strong. Based on Supplementary Equation (15), when on resonance, the scattering cross section can be calculated analytically as

$$\frac{\sigma}{\sigma_0} = \left(\frac{\Gamma_0^2}{\Gamma_0^2 + 2\Omega^2} \right)^2, \tag{27}$$

where Ω denotes the Rabi frequency. We now vary Ω and check the scattering cross section obtained via our nonlinear FDFD solver. The simulation results are displayed in Supplementary Fig. 3. In Supplementary Fig. 3(a) we compare two different scenarios, namely $\Omega/\Gamma_0 = 0.01$ and $\Omega/\Gamma_0 = 2$. The TLS is placed at the middle. The scattered field distributions are different for these two cases, since for the second case the TLS already begins to saturate. Detailed comparison can be found in Supplementary Fig. 3(b). The cross sections calculated using our FDFD agrees perfectly with the analytical results, justifying the correctness of our numerical scheme.

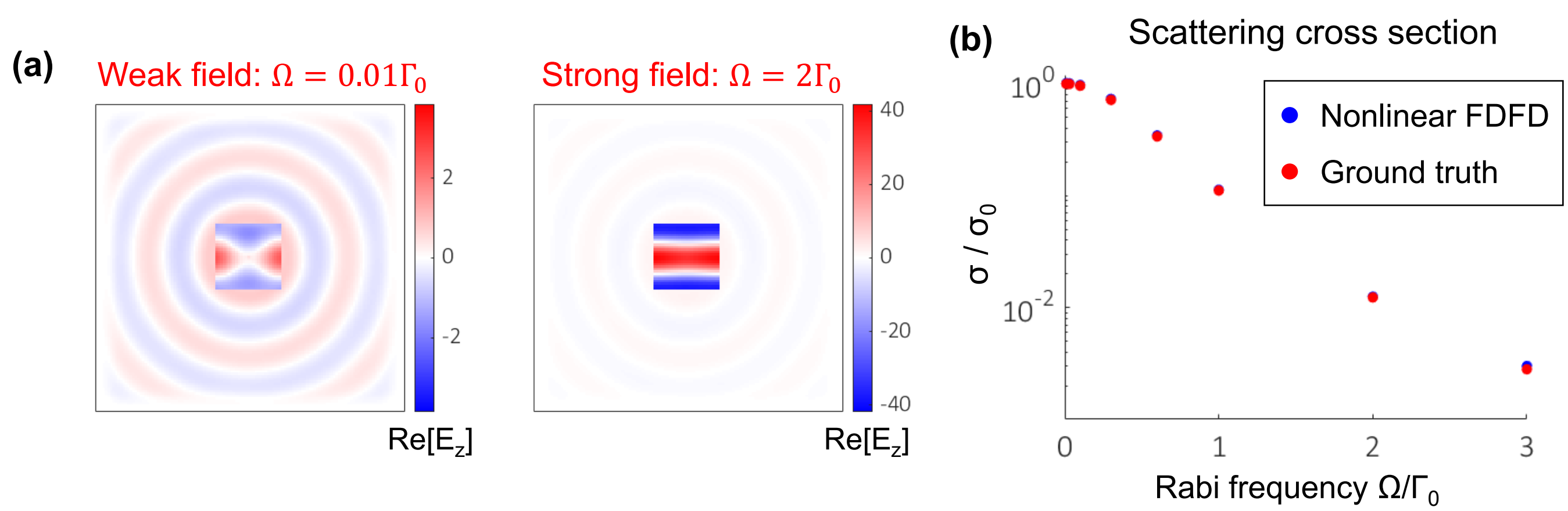

Supplementary Figure 3. **Benchmark: scattering cross section of a single TLS.** (a) The $\mathrm{Re}[E_z]$ field distributions for weak incidence ($\Omega/\Gamma_0 = 0.01$) and strong incidence ($\Omega/\Gamma_0 = 2$). The square region in the middle marks the total-field region, introduced to provide plane-wave excitation. (b) Numerical results of σ/σ_0 under different Rabi frequencies. Our FDFD matches perfectly well with the ground truth.

SUPPLEMENTARY NOTE 4. ADJOINT-BASED OPTIMIZATION

A. Why optimization is required

In this part we explain why we turn to adjoint optimization when designing the quantum-enhanced activation unit. We start from a simple implementation by embedding a quantum emitter inside a silicon waveguide (250 nm width). The quantum emitter has a radiative decay rate of $\Gamma_0 = 2\pi \times 94$ MHz when placed inside homogeneous environment. The input-output relationship of such a waveguide QED system is displayed in Supplementary Fig. 4(a). Here the input x and the output $f(x)$ are calculated by multiplying the electric field amplitude with $\frac{d_0}{\Gamma_0 \hbar}$. Although the intensity threshold is low ($\sim$ nW/μm^2), the curves only show weak nonlinearity. Such behavior can be understood by inspecting the E_z field distribution: at low intensity the TLS acts like a resonant dipole and scatters the incident field out of the waveguide. Naïvely placing the emitter inside the silicon waveguide leads to a low coupling efficiency.

We further test the effectiveness of this activation function, using the technique developed in Refs. 31, 32. We first construct a 24-layer ONN, plugging in this activation unit at each layer. All hidden layers contain $N_{\text{dim}} = 512$ neurons. The input data points form a circular trajectory, parameterized by $\theta \in [0, 2\pi]$:

$$\mathbf{x}(\theta) = \sqrt{N} \cdot (\mathbf{u}\cos(\theta) + \mathbf{v}\sin(\theta)), \tag{28}$$

where $\mathbf{u}$ and $\mathbf{v}$ are two random unit vectors which form an orthonormal basis for a 2D subspace. The trajectory contains 10000 sampling points, distributed uniformly on $\theta \in [0, 2\pi]$. We track the evolution of such a trajectory during the forward-propagation process, as shown in Supplementary Fig. 4(b). For all the data trajectories we carry out a dimension reduction through principal component analysis (PCA), so that they can be visualized as curves in 3D space. It can be observed that the trajectory only deforms slightly. This is consistent with our understanding that using an activation function that is only weakly nonlinear leads to poor expressivity. Based on such intuition, we turn to adjoint optimization and see whether we can find a structure that increases the interaction between the emitter and the incident light and the emitter, which hopefully results in a stronger nonlinearity.

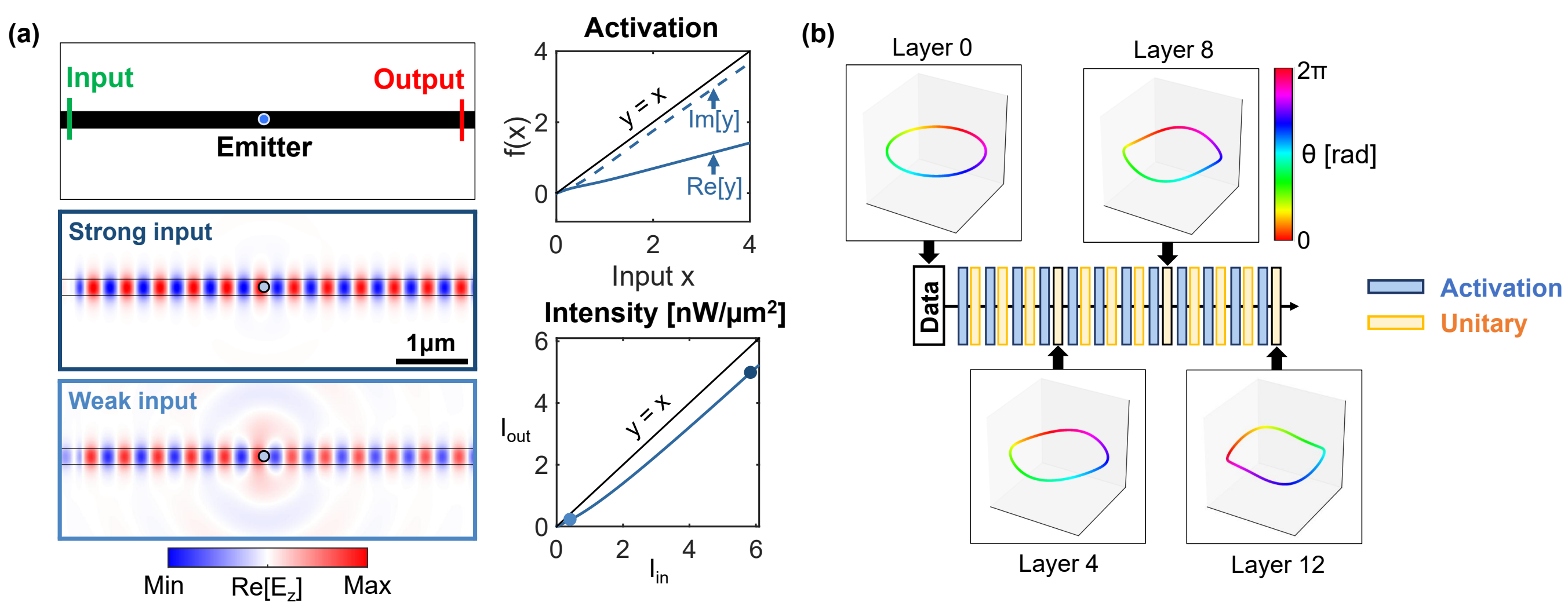

Supplementary Figure 4. **Waveguide-based activation unit.** (a) The input-output relationship shows weak nonlinearity. (b) The evolution of data trajectory during forward-propagation. Since the activation function is only weakly nonlinear, the trajectory is only deformed slightly, indicating low expressive power.

B. Adjoint method

In this part we explain how the conventional adjoint-based optimization can be generalized to our case, where we solve for nonlinear Maxwell's equations. Based on Supplementary Equation (20), the physical constraint is set by the following nonlinear equation:

$$\mathbf{A}\mathbf{x} - \mathbf{b}_0 - \alpha_{\text{TLS}} \cdot \mathbf{M} \cdot f\left(\mathbf{x} - \beta_{\text{rad}}\mathbf{A}\mathbf{x} + \beta_{\text{rad}}\mathbf{b}_0\right) = \mathbf{0}, \tag{29}$$

where the matrix $\mathbf{A} = \mathbf{A}(\mathbf{p})$ depends on a set of design parameters $\mathbf{p}$. We would like to optimize for a given target function, denoted as $L(\mathbf{x})$. Without loss of generality, here we assume that L does not depend explicitly on $\mathbf{p}$. Our aim is to calculate its gradient $\frac{dL}{dp_i}$ w.r.t. the design parameters. To achieve this, we first differentiate Supplementary Equation (29) w.r.t. parameter p_i:

$$\frac{\partial \mathbf{A}}{\partial p_i}\mathbf{x} + \mathbf{A}\frac{d\mathbf{x}}{dp_i} - \alpha_{\text{TLS}}\mathbf{M}\cdot\frac{df}{dp_i} = 0. \tag{30}$$

From the above equation, we can derive

$$\frac{d\mathbf{x}}{dp_i} = -\left(\mathbf{A}^{'}\right)^{-1}\left[\mathbb{1} + \alpha_{\text{TLS}}\cdot\beta_{\text{rad}}\mathbf{M}\cdot\text{diag}\left\{\frac{df}{d\mathbf{s}}\right\}\right]\cdot\frac{\partial \mathbf{A}}{\partial p_i}\mathbf{x}, \tag{31}$$

where we have defined $\mathbf{s} = \mathbf{x} - \beta_{\text{rad}}(\mathbf{A}\mathbf{x} - \mathbf{b}_0)$, and $\mathbf{A}^{'} = \mathbf{A} - \alpha_{\text{TLS}}\mathbf{M}\cdot\text{diag}\left\{\frac{df}{d\mathbf{s}}\right\}\cdot(\mathbb{1} - \beta_{\text{rad}}\mathbf{A})$. We now define the adjoint as

$$\mathbf{x}_{\text{adj}} = -\left(\mathbf{A}^{'}\right)^{-T}\frac{\partial L}{\partial \mathbf{x}}, \tag{32}$$

which can be calculated by solving the following equation:

$$\left(\mathbf{A}^{'}\right)^{T}\mathbf{x}_{\text{adj}} = -\frac{\partial L}{\partial \mathbf{x}}. \tag{33}$$

Note that although the forward simulation involves solving a nonlinear equation, the adjoint simulation only involves solving a linear equation. Finally, we arrive at the gradient

$$\frac{dL}{dp_i} = \left(\frac{\partial \mathbf{A}}{\partial p_i}\mathbf{x}\right)^{T}\left[\mathbb{1} + \alpha_{\text{TLS}}\cdot\beta_{\text{rad}}\mathbf{M}\cdot\text{diag}\left\{\frac{df}{d\mathbf{s}}\right\}\right]\cdot\mathbf{x}_{\text{adj}}. \tag{34}$$

Based on the above Supplementary Equation (34), the calculated gradient can be used to update the design parameters $\mathbf{p}$.

C. Material platform of the activation unit

In the revised proposal, the nonlinear activation unit is designed on a realistic GaP-on-diamond platform, and verified with 3D full-wave simulations. The device operates at the SiV^- zero-phonon line $\lambda_0 = 737.134$ nm [33]. It consists of a patterned top GaP layer of thickness $h_{\text{wg}} = 200$ nm on an unetched thin-film diamond layer ($t = 160$ nm) [34], sitting on the SiO_2 substrate. The refractive indices of the involved materials are $n_{\text{GaP}} = 3.22$, $n_{\text{diamond}} = 2.40$, and $n_{\text{SiO}_2} = 1.45$. We choose GaP, not only because it is transparent at 737 nm, but also because similar structures have been demonstrated in experiments [35, 36]. Light is coupled into and out of the device through GaP strip waveguides ($w_{\text{wg}} = 260$ nm wide, $h_{\text{wg}} = 200$ nm thick), propagating along x direction. We use the fundamental TE mode with effective index $n_{\text{eff}} = 2.646$. The waveguide cross-section and the corresponding guided-mode profile are shown in Supplementary Fig. 5.

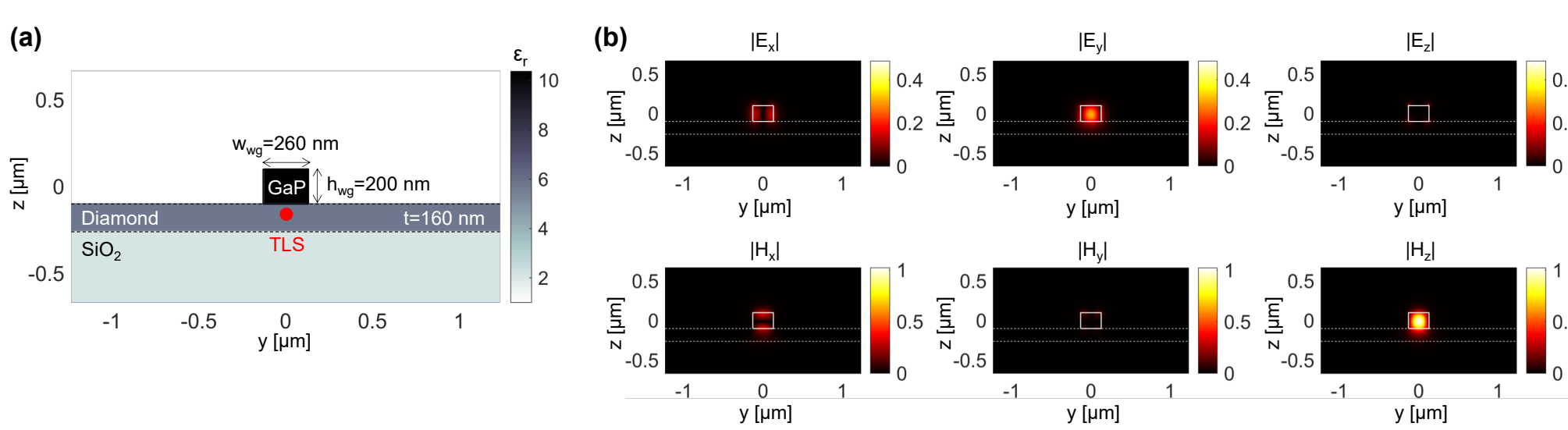

Supplementary Figure 5. **Input waveguide of the 3D activation unit.** (a) Cross-section of the GaP-on-diamond strip waveguide. (b) Fundamental TE_0 guided-mode profile at $\lambda_0 = 737.134$ nm, with effective index $n_{\text{eff}} = 2.646$.

The nonlinearity is provided by a single SiV^- color center, modeled as a two-level system with its dipole moment oriented along y (transverse to the propagation direction). The SiV^- is positioned 30 nm below the diamond surface. Such single emitters can be created deterministically by counted ion implantation, which has already been demonstrated experimentally [37, 38]. The emitter is assumed to operate at cryogenic temperature $T = 4\text{K}$ [39].

D. Optimization setup

In this part we provide details related to optimizing the activation unit.

3D activation unit (GaP-on-diamond). The design region is $1.5 \times 0.7\ \mu\text{m}^2$, located in the top GaP layer of the platform described above, with the single SiV^- emitter at its center. A conic filter with a radius of 100 nm is applied to control the minimum feature size.

2D study (silicon-on-insulator). The 2D results presented in this paper were instead obtained on a silicon-based platform. The design region is $5\ \mu\text{m} \times 1\ \mu\text{m}$. TLSs are placed at the middle part of the design region. The spacing between two neighboring TLSs is fixed as $1\ \mu\text{m}$. A waveguide mode ($E_z \neq 0$; the effective refractive index is $n_{\text{eff}} = 2.951$) serves as the input.

For both the 2D and 3D designs, the relative permittivity at each in-plane location (x, y) inside the design region is parameterized as

$$\epsilon_r(x,y) = \epsilon_{\text{background}} + (\epsilon_{\text{structure}} - \epsilon_{\text{background}}) \cdot \frac{1}{1 + \exp\left[-\beta \cdot p(x,y)\right]}, \tag{35}$$

For the 2D design, the background and structural materials are SiO_2 and Si, respectively, such that $\epsilon_{\text{background}} = \epsilon_{\text{SiO}_2} = 1.44^2$ and $\epsilon_{\text{structure}} = \epsilon_{\text{Si}} = 3.47^2$. For the 3D design, they are vacuum and GaP, respectively, such that $\epsilon_{\text{background}} = \epsilon_{\text{vac}} = 1$ and $\epsilon_{\text{structure}} = \epsilon_{\text{GaP}} = 3.22^2$. The parameter β controls the binarization process and is gradually increased from 5.0 to 150.0 for the 2D design and from 5.0 to 200.0 for the 3D design throughout the optimization.

We now introduce the figure-of-merit (FoM) that we are trying to optimize. In order to achieve a strong nonlinearity, we consider two extreme cases:

(1) **case 1**: suppose the incident wave is so strong that all TLSs have saturated. The TLSs are transparent and can be ignored.

(2) **case 2**: suppose the incident wave is very weak, such that the TLSs can be treated as linear dipoles under the weak-excitation limit.

The above two cases are shown in Supplementary Fig. 6 schematically. The FoM consists of two parts. Specifically, for case i ($i = 1, 2$), denote the amplitude measured at the output port as a_i, which is a complex scalar, calculated from the inner product between the electromagnetic field distribution and the desired waveguide mode. The input amplitude is normalized to be 1. First, for case 1 the transmission should be maximized:

$$\text{FoM}_1 = |a_1|^2. \tag{36}$$

Second, in order to show strong nonlinearity, the difference between a_1 and a_2 should be as large as possible:

$$\text{FoM}_2 = |a_2 - a_1|^2. \tag{37}$$

The final FoM we intend to optimize can be expressed as a weighted sum of these two terms:

$$\text{FoM} = \lambda_1 \cdot \text{FoM}_1 + \lambda_2 \cdot \text{FoM}_2, \tag{38}$$

where we choose $\lambda_1 = 1.0$ and $\lambda_2 = 2.0$ for all the designs. Note that in practice, an extra penalty term is included to minimize the field intensity outside the design region. This term (neglected in the above equation for the sake of brevity) helps us prevent neighboring activation units from coupling to each other.

We carry out gradient descent w.r.t. parameter $p(x, y)$ and iterate for 500 steps to ensure convergence. Adam optimizer is used. The step size, initialized to be 0.02, is decreased by a factor of 0.9958 after each iteration.

E. Understanding $N_{\text{TLS}} = 2$ with coupled-mode theory

We recorded the FoM values obtained during the optimization of the 2D activation unit, varying the number of TLSs N_{TLS} from 1 to 4, as visualized in Supplementary Fig. 6. We observe that using $N_{\text{TLS}} > 1$ leads to a much higher FoM. As shown below using coupled-mode theory, a single resonant dipole cannot modify the transmission coefficient from -1 to 1, which limits the achievable nonlinear contrast. While using $N_{\text{TLS}} \geqslant 2$ improves the performance in this 2D study, deterministically placing more than one emitter inside the same compact device would significantly increase the experimental difficulty. For this reason, our final 3D proposal uses a single emitter ($N_{\text{TLS}} = 1$), which still provides sufficient nonlinear contrast for the tasks demonstrated in this work.

In this part we use coupled-mode theory to prove why using $N_{\text{TLS}} = 1$ leads to low FoM. Consider a two-port system, as shown in Supplementary Fig. 7(a). We define the input vector as $|s^+\rangle = (s_1^+, s_2^+)^T$, and the output vector

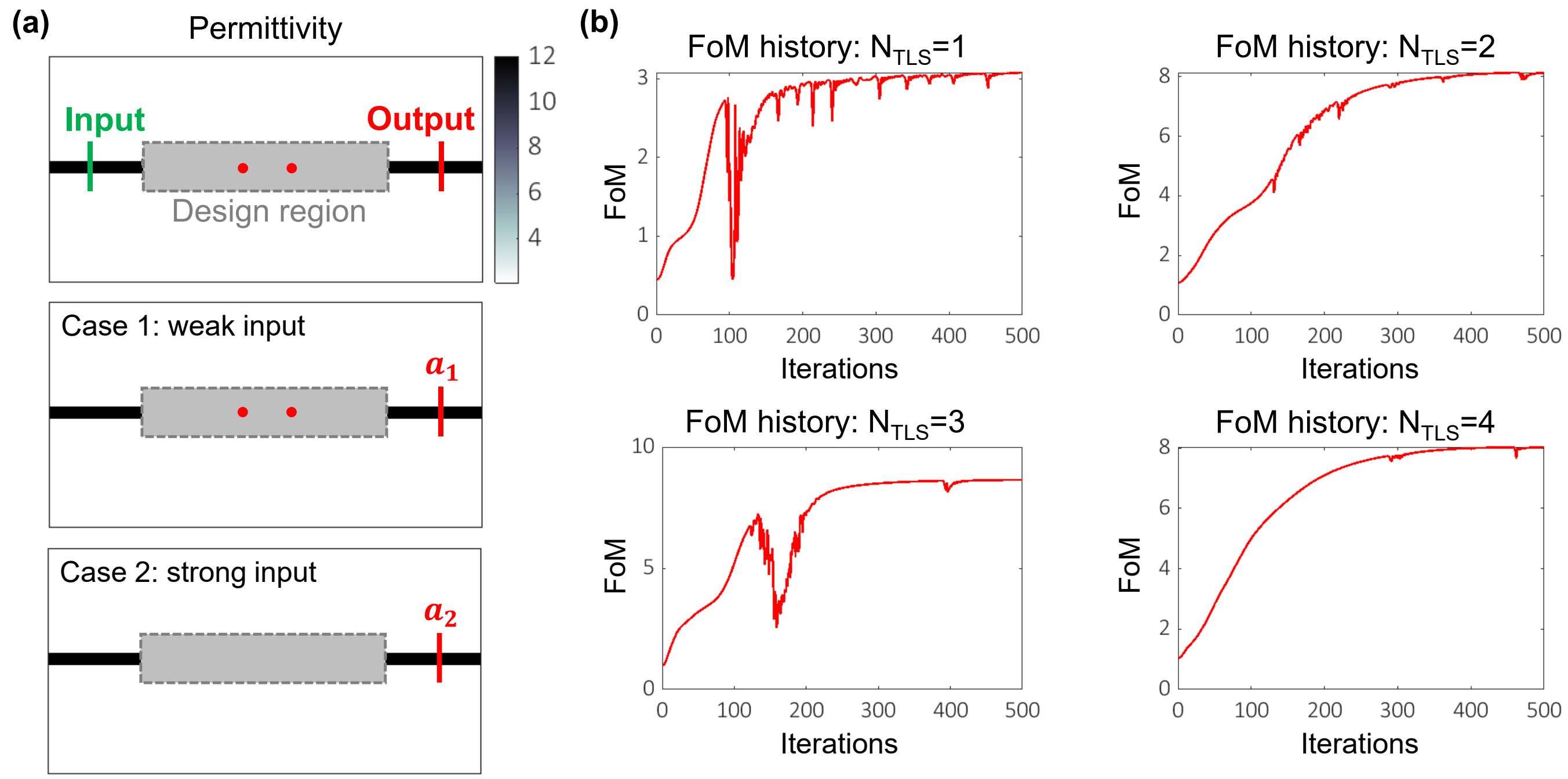

Supplementary Figure 6. **Optimization of the activation unit (2D).** (a) Schematic illustration of the two extreme cases considered. (b) FoM curves obtained throughout the optimization. We vary the number of included emitters $N_{\mathrm{TLS}} \in \{1, 2, 3, 4\}$ and find that $N_{\mathrm{TLS}} \geqslant 2$ results in much better performance.

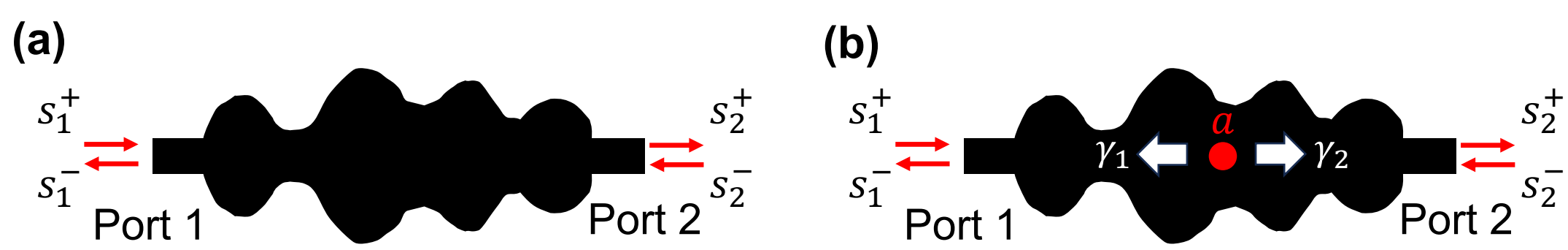

Supplementary Figure 7. **Modeling the 2-port system using coupled-mode theory.** (a) Without the emitter, the structure forms a background. (b) Including $N_{\mathrm{TLS}} = 1$ emitter, which is treated as a single-mode resonator and couples to both ports.

as $|s^-\rangle = (s_1^-, s_2^-)^T$. Without the resonant dipole, we denote the S-matrix of the background structure as a 2×2 matrix $\mathbf{C}$:

$$|s^-\rangle = \mathbf{C}|s^+\rangle. \tag{39}$$

The system is assumed to be passive. Now suppose a resonant dipole is included inside the structure, as shown in Supplementary Fig. 7(b). This resonant dipole is treated as a single-mode resonator, and the entire system can be modeled using temporal coupled-mode theory (TCMT):

$$\frac{da}{dt} = (-i\omega_0 - \gamma)a + \langle d|s^+\rangle, \tag{40}$$

where $\gamma = \gamma_1 + \gamma_2$ denotes the total decay rate, and $|d\rangle = (d_1, d_2)^T$ depicts the coupling coefficients between the resonator and the two ports. These coefficients satisfy $\gamma_1 = \frac{1}{2}|d_1|^2$ and $\gamma_2 = \frac{1}{2}|d_2|^2$, which can be derived based on energy conservation constraint [40]. The output now includes two contributions:

$$|s^-\rangle = \mathbf{C}|s^+\rangle + a|d\rangle. \tag{41}$$

Suppose we work under single frequency ω. By applying the replacement $\frac{d}{dt} \to -i\omega$, Supplementary Equation (40) can be solved to obtain

$$a(\omega) = \frac{\langle d|s^+\rangle}{-i\Delta + \gamma}, \tag{42}$$

where detune $\Delta = \omega - \omega_0$. When on-resonance, the S-matrix of the entire system (defined as $|s^-\rangle = \mathbf{S}(\omega)|s^+\rangle$) can be derived as

$$\mathbf{S}(\omega) = \mathbf{C}(\omega) + \frac{|d\rangle\langle d|}{-i\Delta + \gamma}, \tag{43}$$

which, when on resonance ($\Delta = 0$), can be further simplified as

$$\mathbf{S}(\omega) = \mathbf{C}(\omega) + \frac{|d\rangle\langle d|}{\gamma}. \tag{44}$$

Without the resonant dipole, the transmission coefficient is $t = C_{21}$; with the dipole presented, the transmission coefficient becomes $t' = C_{21} + \frac{d_1^* d_2}{\gamma}$. The difference between t' and t can be simplified as

$$|t' - t| = \left|\frac{d_1^* d_2}{\gamma}\right| = \frac{|d_1| \cdot |d_2|}{\gamma} = \frac{2\sqrt{\gamma_1\gamma_2}}{\gamma_1 + \gamma_2} \leqslant 1. \tag{45}$$

When using only $N_{\text{TLS}} = 1$ emitter, we have proved that for the two extreme cases shown in Supplementary Fig. 6, their transmission coefficients can differ by $|\Delta t| \triangleq |t' - t| \leqslant 1$. This helps explain why the FoM cannot exceed 3 in Supplementary Fig. 6(b): since $|a_1| \leqslant 1$ and $|a_2 - a_1| \leqslant 1$, Supplementary Equation (38) gives FoM $\leqslant \lambda_1 + \lambda_2 = 3$ given that $\lambda_1 = 1.0$ and $\lambda_2 = 2.0$. Using $N_{\text{TLS}} \geqslant 2$ emitters will break this restriction, boosting the performance to FoM > 8.

F. Final result

1. 3D activation unit

We first present our main result, in which the nonlinear activation unit is designed on the GaP-on-diamond platform described above. As shown in Supplementary Fig. 8(a), the inverse-designed structure is a binary GaP pattern, with a single SiV^- emitter embedded 30 nm below the diamond surface. The input-output relationship $S_{21}(x)$, obtained from the nonlinear FDFD simulation, is displayed in Supplementary Fig. 8(b). The transmission $|S_{21}|^2$ rises from zero (weak-field limit) to 0.86 (strong-field limit). Here we assume that the $S_{21}(x)$ curve takes a sigmoid-like form:

$$S_{21}(x) = b_{\text{offset}} + \frac{Ae^{i\phi}}{1 + \exp\left[-\beta(\log_{10}(x) - x_0)\right]}, \tag{46}$$

Here b_{offset} is a complex offset, A and ϕ are the amplitude and phase of the sigmoid contribution, β controls the sigmoid steepness, and x_0 sets the offset along the logarithmic input axis. Fitting the curve gives $b_{\text{offset}} = 0.0584 + 0.0603\,i$, $A = 0.8602$, $\phi = 1.5551$, $\beta = 7.972$, and $x_0 = 0.0299$. The fitted activation is then implemented in PyTorch and used as the nonlinear activation when training the all-optical neural network. With this activation unit, the three-class spiral classification task is solved perfectly, as shown in Fig. 2 of the main text.

The resulting nonlinear threshold can be as low as a few nW/μm^2. This can be understood intuitively based on simple back-of-envelope calculation: for a two-level system whose dipole moment matrix element is d_0, its radiative decay rate is $\Gamma_0 = \frac{n d_0^2 \omega_0^3}{3\pi\hbar\epsilon_0 c_0^3}$. Here n denotes the refractive index of the emitter's environment. In order to observe saturation effect, the incident electric field E_{inc} should satisfy $\frac{d_0 E_{\text{inc}}}{\hbar} \sim \Gamma_0$. The physical intuition behind this equation is that the Rabi frequency should be comparable to its decay rate Γ_0. A quick estimation can be made by taking $n = 2.4$ (corresponds to the refractive index of diamond at 737 nm) and $\Gamma_0 = 2\pi \times 94$ MHz. The dipole moment can be estimated as $d_0 \approx 17.7$ Debye. The electric field that's required can be estimated as $E_{\text{inc}} \approx 1.05 \times 10^3$ V/m, corresponding to a light intensity of $I_{\text{inc}} = \frac{n E_{\text{inc}}^2}{2\eta_0} \approx 3.5$ nW/μm^2.

2. 2D activation unit

We also show the performance of our 2D activation unit, which embeds two quantum emitters inside a silicon structure. This 2D unit serves two purposes: it provides the activation function used in the reinforcement learning demonstrations of Fig. 3 in the main text, and its trajectory evolution offers intuition on how the form of the activation function affects the expressive power. The input-output relationship of this activation unit is displayed

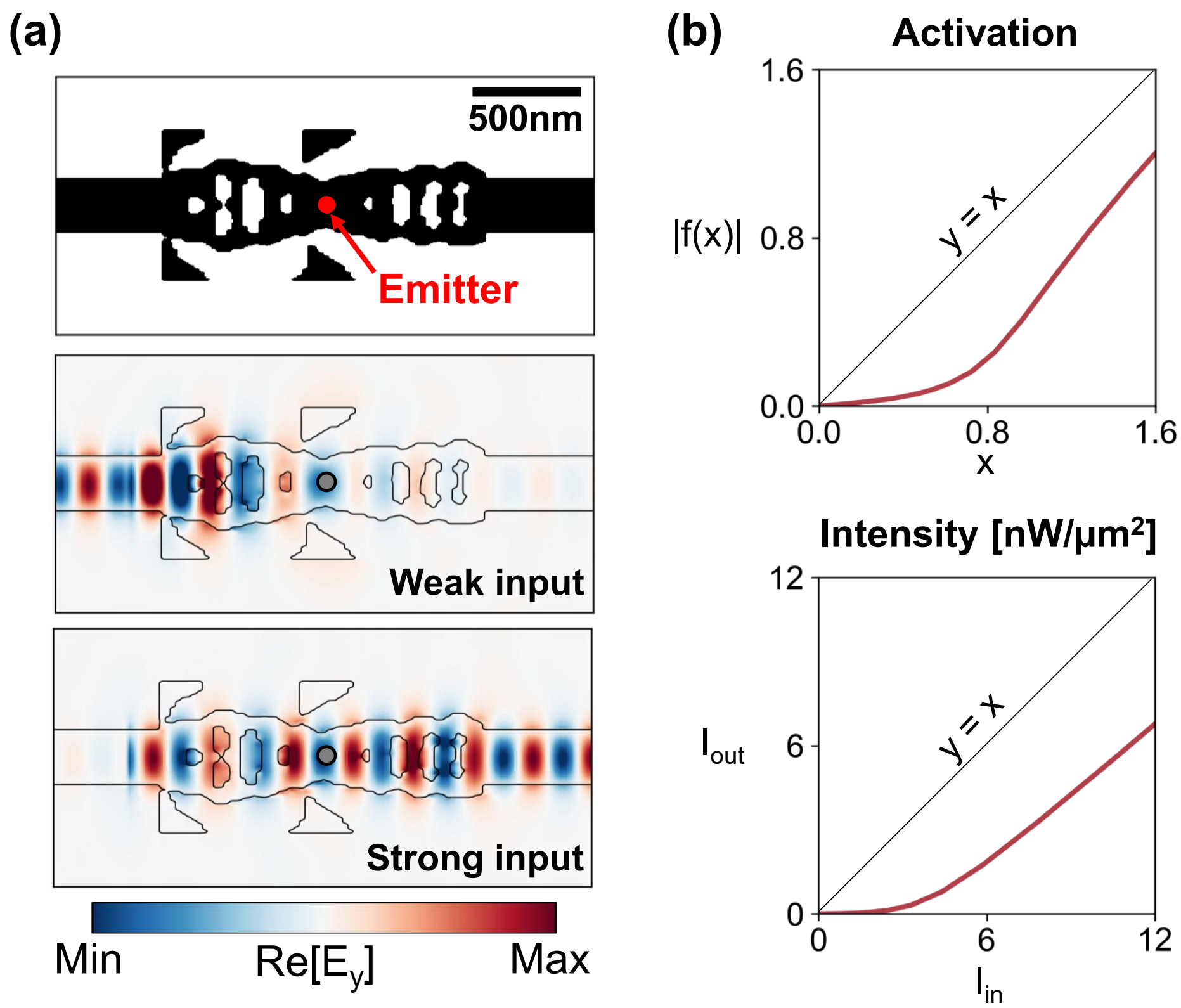

Supplementary Figure 8. **3D inverse-designed activation unit.** (a) Optimized binary GaP pattern in the $1.5 \times 0.7\ \mu\text{m}^2$ design region (black: 200 nm GaP; white: fully etched), with a single SiV^- emitter placed 30 nm below the diamond surface and dipole oriented along y. The $\text{Re}[E_y]$ field distributions of two extreme cases are visualized. (b) Nonlinear input-output activation function obtained from full-wave 3D nonlinear FDFD.

in Supplementary Fig. 9(a). Contrary to the waveguide-based implementation, this time the curves show strong nonlinearity. By inspecting the E_z field distribution we realize that the designed structure tries to focus the incident beam onto the two quantum emitters, thus forming two hot spots. This enhances the light-matter interaction, leading to a strongly nonlinear response.

Again, we test the effectiveness of this activation function by tracking the evolution of a circular trajectory during the forward-propagation process. All the experimental settings remain the same as the previous case. We do a curve fitting based on the input-output relationship obtained from FDFD. Specifically, denote the input as x, the output $y = S_{21}(x) \cdot x$. The S-parameter, which now depends on x, is fitted using the following form:

$$S_{21}(x) = b_{\text{offset}} + w_1(x) \cdot A_1 e^{i\phi_1} + w_2(x) \cdot A_2 e^{i\phi_2}, \tag{47}$$

where the weights $w_1(x)$, $w_2(x)$ are sigmoid-like functions:

$$\begin{aligned} w_1(x) &= \frac{1}{1 + \exp\left[-\beta_1(\log_{10}(x) - x_{10})\right]}, \\ w_2(x) &= 1 - \frac{1}{1 + \exp\left[-\beta_2(\log_{10}(x) - x_{20})\right]}. \end{aligned} \tag{48}$$

The curve fitting results are shown in Supplementary Fig. 10. The fitted parameters are summarized as follows: $A_1 = 0.9250$, $\phi_1 = -3.0528$, $A_2 = 0.9675$, $\phi_2 = 0.0747$, $b_{\text{offset}} = -0.0526$, $\beta_1 = 67.8915$, $x_{10} = -0.6538$, $\beta_2 = 10.1345$, $x_{20} = -0.5311$.

The fitted curve is then implemented in PyTorch (version 2.4.1) and used as the activation function for training ONNs. As shown in Supplementary Fig. 9(b), this time the trajectory changes very quickly as the network depth increases. The total curvature increases quickly, revealing that the designed ONN has strong expressive power.

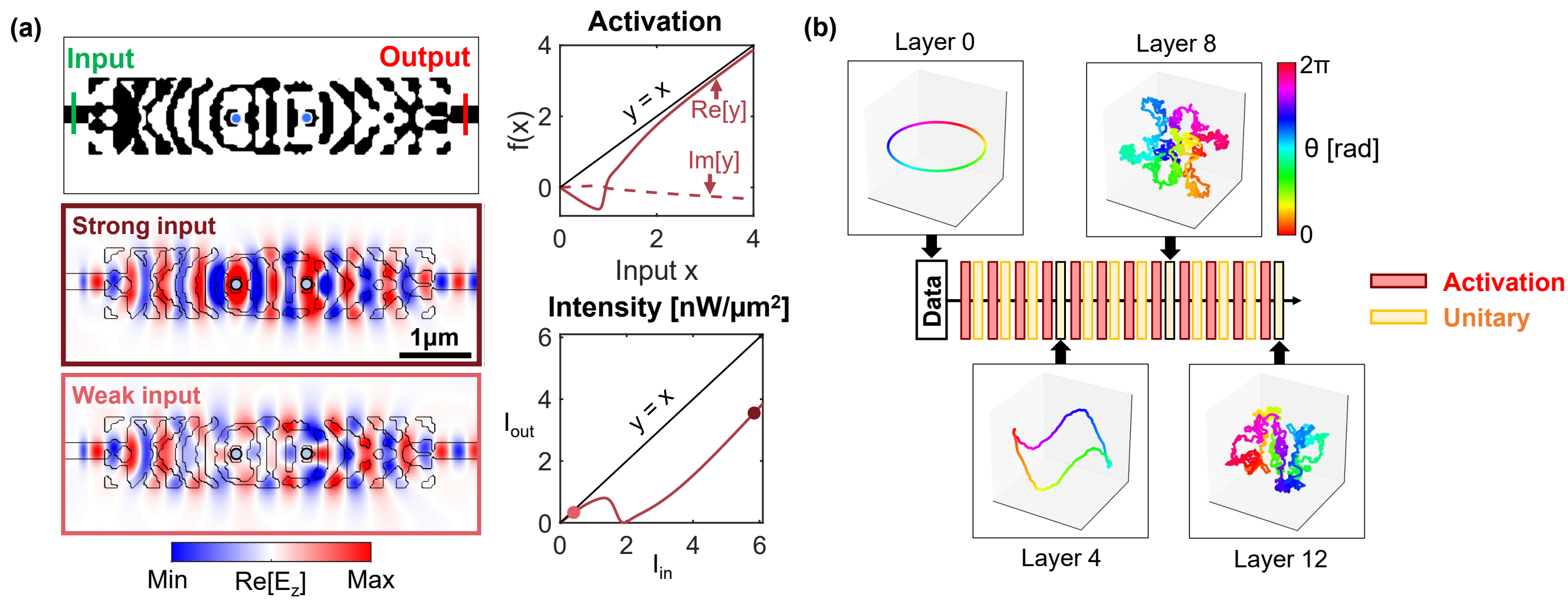

Supplementary Figure 9. **Inverse-designed activation unit.** (a) The input-output relationship shows strong nonlinearity. (b) The evolution of data trajectory during forward-propagation. The trajectory is deformed strongly during the forward-propagation, indicating high expressive power.

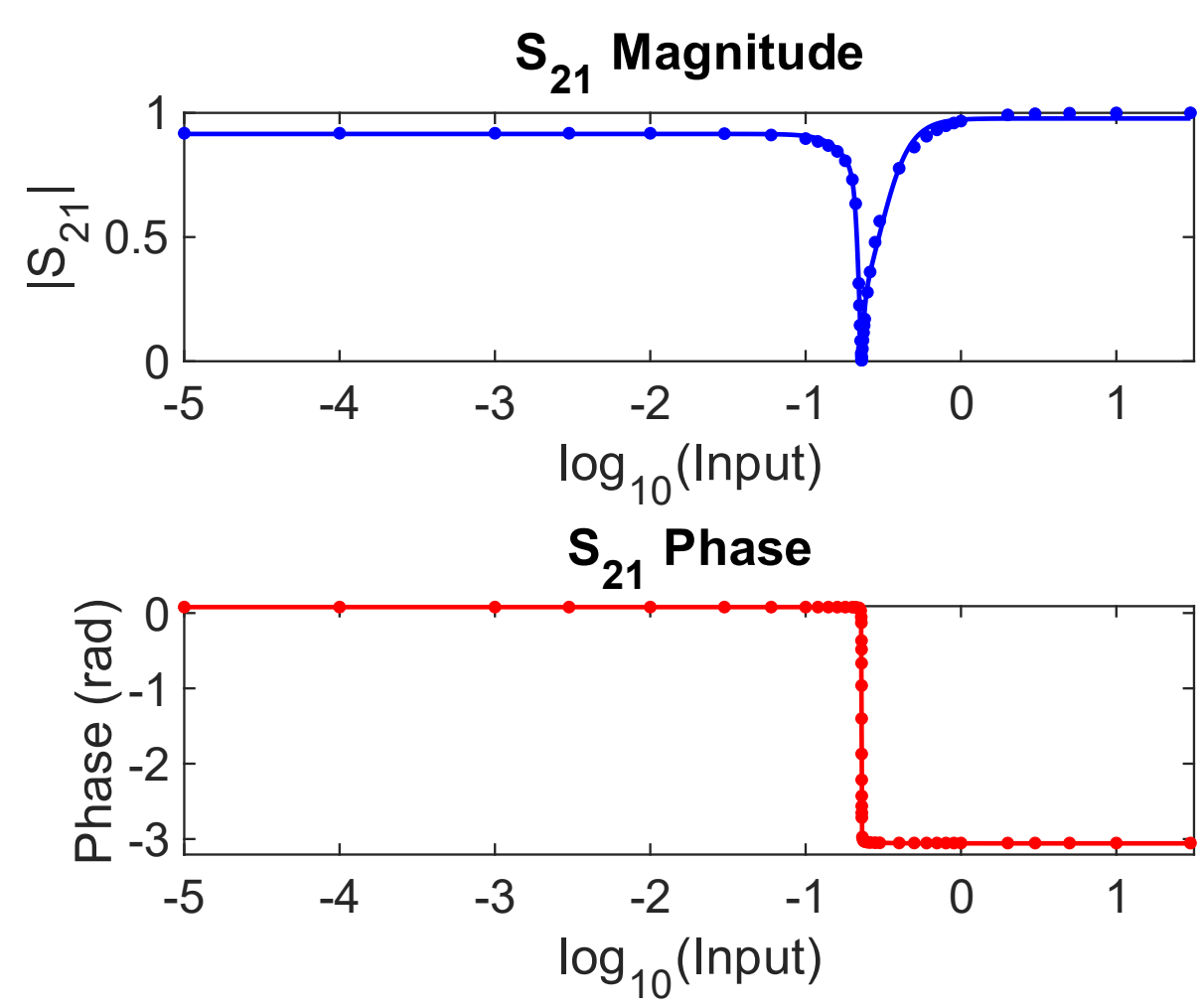

Supplementary Figure 10. **Curve fitting for the nonlinear activation function.** The upper panel displays the magnitude $|S_{21}|$, while the lower panel displays the phase $\angle S_{21}$. The circular markers show results obtained via nonlinear FDFD, while the solid curves represent the fitting results.

G. Inverse design of linear blocks

Finally, we provide details regarding how the linear blocks are designed via adjoint optimization. The linear blocks are passive elements that route light to realize the trained weight matrices. We design them with a 2.5D variational effective-index method (vEIM) [41], which is far cheaper than full 3D optimization. The blocks are GaP-on-diamond devices working at the same wavelength $\lambda_0 = 737.134$ nm. The top GaP layer is patterned by a 40 nm shallow etch, switching between an unetched state (200 nm) and an etched state (160 nm). The vEIM projects the layered structure onto a single reference vertical mode, and collapses it into a 2D effective permittivity map $\epsilon_{\text{eff}}(x, y)$. The two states correspond to effective indices $n_{\text{eff}} = 2.89$ (unetched) and $n_{\text{eff}} = 2.76$ (etched). Such effective-index approach has been demonstrated experimentally for matrix-vector multiplication [42].

Within this 2.5D model, we run adjoint optimization based on FDFD for each layer, so that the device S-matrix reproduces the target weight matrix. For any given location inside the design region, the permittivity distribution is parameterized the same way as Supplementary Equation (35), now interpolating between the etched and unetched effective permittivities. To ensure a fabricable structure, a conic filter with a radius of 200 nm is applied, which helps

control the minimum feature size. The parameter β, which controls the binarization process, is increased from 5.0 to 200.0 gradually throughout the optimization, which guarantees a fully binarized final design. We carry out gradient descent w.r.t. parameter $p(x, y)$ and iterate for 300 steps to ensure convergence. Adam optimizer is used, with default parameter $\beta_1 = 0.9$ and $\beta_2 = 0.999$. The step size is decreased by a factor of 0.994 after each iteration. With this procedure, each optimized block reproduces its target weight matrix to within 4–8% relative error. The resulting device, matrix comparison, and optimization history are summarized in Supplementary Fig. 11.

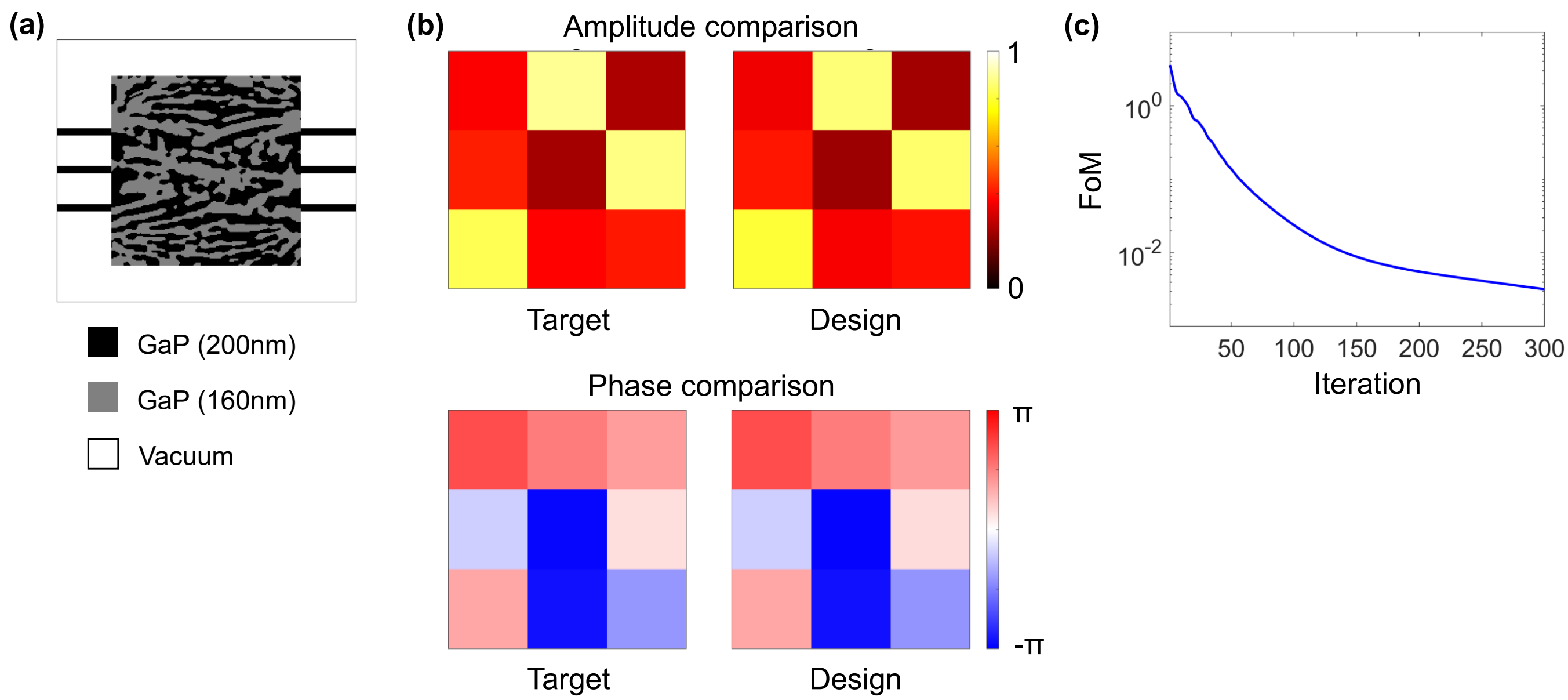

Supplementary Figure 11. **Example of inverse-designed linear block.** (a) The structure consists of etched GaP layer on top of thin-film diamond. (b) Comparison between the S-matrix of the designed structure and the targeted unitary matrix. (c) FoM curve obtained during the optimization. The FoM is defined as the Frobenius norm of the difference between the S-matrix and the target unitary matrix.

SUPPLEMENTARY NOTE 5. EFFECT OF LOW QUANTUM EFFICIENCY

In this part we will show how a non-unity quantum efficiency will affect the design of our activation unit. For the non-ideal case, denote the quantum efficiency as $\eta_Q = \Gamma_0/(\Gamma_0 + \Gamma_{\mathrm{nrad}}) < 1$, where Γ_{nrad} denotes the non-radiative decay rate. As derived in Supplementary Note 2, a reduced η_Q rescales the dressed dipole moment and weakens the coupling between the emitter and the optical field. It enters the nonlinear FDFD formalism through the replacement $f(x) \to f_{\eta_Q}(x) \triangleq f(\eta_Q x)$.

We fix the total decay rate as $\Gamma_0 + \Gamma_{\mathrm{nrad}} = 2\pi \times 94$ MHz, then repeat the 3D inverse design for different quantum efficiencies $\eta_Q \in \{20\%, 40\%, 60\%, 80\%, 100\%\}$. The results are displayed in Supplementary Fig. 12. Specifically, Supplementary Fig. 12(a) shows that the best achievable FoM drops as the quantum efficiency decreases. The resulting transmission curves are plotted in Supplementary Fig. 12(b). For $\eta_Q \geqslant 60\%$, the nonlinear activation retains a similar shape. Below $\eta_Q \approx 40\%$, however, the contrast collapses and the design becomes a transparent waveguide, because the weak-field response can no longer be suppressed once the radiative coupling becomes too weak. We'd like to point out that the quantum efficiency of SiV^- centers has been measured to reach $\sim$60% [43]. Therefore we believe that our proposal remains valid with realistic emitters.

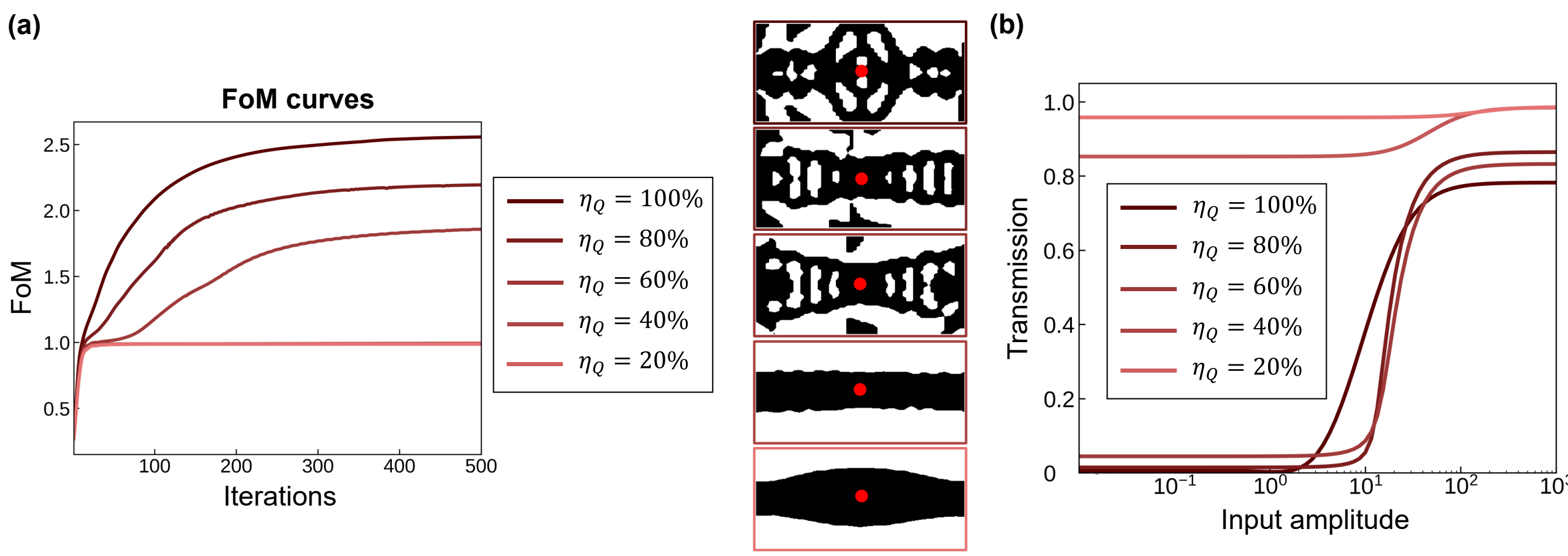

Supplementary Figure 12. **The effect of non-unity quantum efficiency**. (a) FoM curves obtained during the adjoint-based optimization. (b) The resulting transmission curves $|S_{21}|^2$ for $\eta_Q \in \{20\%, 40\%, 60\%, 80\%, 100\%\}$. The nonlinear activation is retained for $\eta_Q \geqslant 60\%$, while the contrast collapses below $\eta_Q \approx 40\%$.

SUPPLEMENTARY NOTE 6. PERFORMANCE ON SUPERVISED LEARNING TASKS

In this part we display the performance of our ONN on two supervised learning tasks: MNIST dataset [44] as well as FashionMNIST dataset [45]. While these two datasets are simple, the results serve two purposes: first, by comparing the performance difference between nonlinear ONN and linear ONN, the effectiveness of our proposed activation function is verified; second, as we will show, a linear model can already solve the task reasonably well, which is consistent with existing literature. While the training is carried out in a physics-aware manner, all the models involved in this part have not been translated into concrete photonic devices, due to their high input dimensionality: designing linear blocks of this size by adjoint optimization is computationally expensive within our current pipeline.

A. MNIST

The MNIST dataset consists of 28×28 images of hand-written digits. These images are first resized to 14×14, then flattened into a 196-dim vector, which serves as the input of our ONN. The ONN consists of two hidden layers and one output layer, each containing 200 neurons. We use the cross entropy loss to train our ONN, which is the standard approach for classification task. Adam optimizer is applied throughout the training process, with a fixed learning rate $\text{lr} = 0.01$. The batch size is fixed as 100. Both models are trained for 30 epochs. The training curves are displayed in Supplementary Fig. 13(a). The final classification accuracy on test dataset is 96.72% (nonlinear) vs. 88.41% (linear). The proposed activation unit leads to a 8.31% improvement.

B. FashionMNIST

The training setup for FashionMNIST are almost identical to that of MNIST. Specifically, the original 28×28 images are resized to 14×14, then flattened into a 196-dim input vector. The ONN consists of two hidden layers and one output layer, each containing 200 neurons. The cross entropy loss is used. Adam optimizer is applied, with a fixed learning rate $\text{lr} = 0.001$. The batch size is fixed as 100. Both models are trained for 30 epochs. The training curves are displayed in Supplementary Fig. 13(b). The final classification accuracy on test dataset is 87.87% (nonlinear) vs. 80.23% (linear). The proposed activation unit leads to a 7.64% improvement.

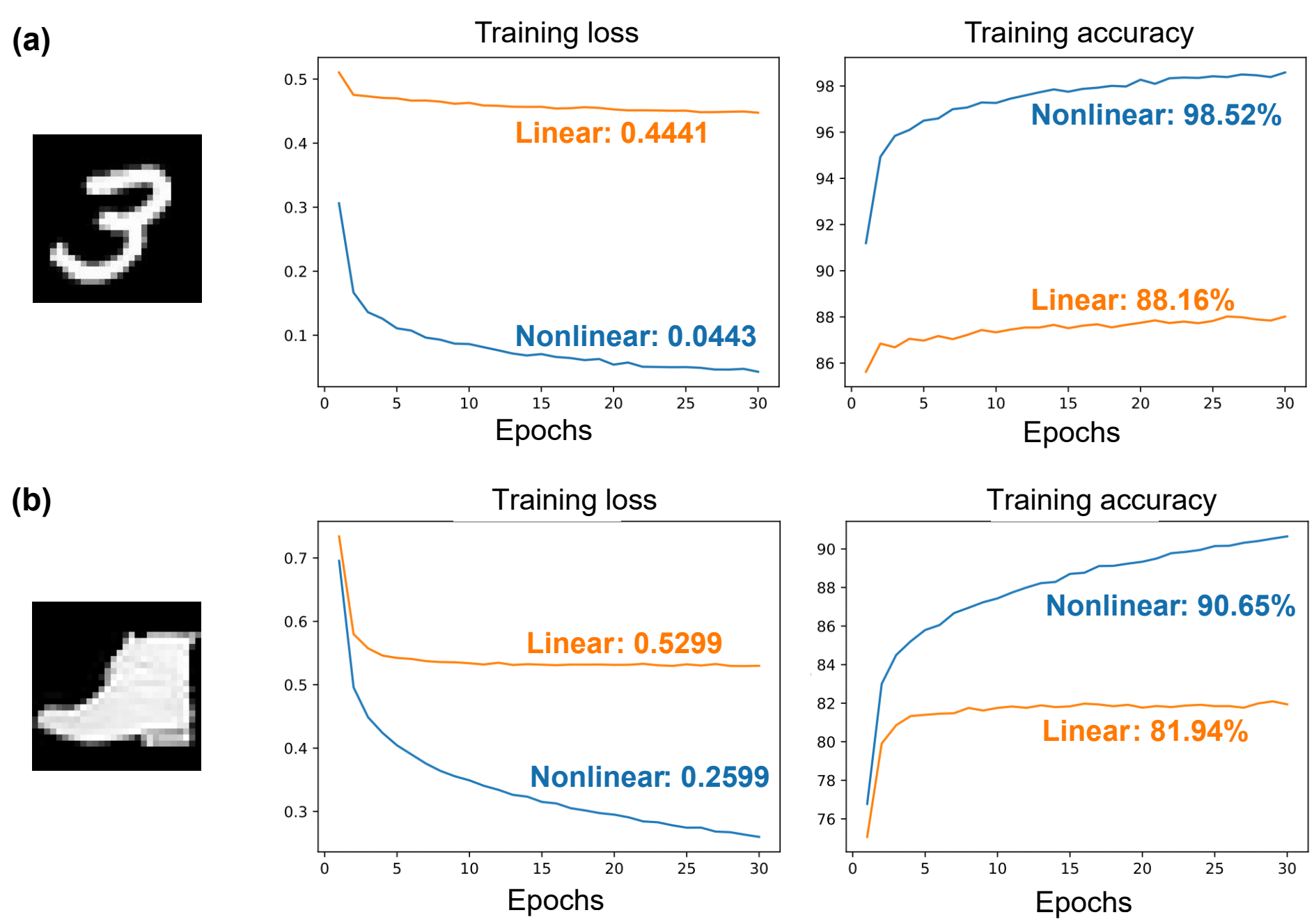

Supplementary Figure 13. **Results for standard supervised learning tasks.** (a) The training curves for MNIST dataset. (b) The training curves for FashionMNIST dataset.

SUPPLEMENTARY NOTE 7. PHYSICS-AWARE TRAINING PROCEDURE

In this part we introduce the physics-aware training procedure in a detailed manner. As shown in Supplementary Fig. 14, the training procedure can be divided into five steps:
(1) **Train**: we first train a digital NN model with the help of PyTorch. Due to the energy-preserving constraint, we restrict the weight matrices $\mathbf{W}^{(i)}$ to be isometric, satisfying $\left\|\mathbf{W}^{(i)}\mathbf{x}\right\|_2^2 = \|\mathbf{x}\|_2^2$. The activation function used comes from Supplementary Equation (47). Beside these differences, this step is almost identical to training a normal multi-layer perceptron (MLP) in PyTorch.
(2) **Dissect**: all complex weight matrices $\mathbf{W}^{(i)}$ can now be extracted from the trained digital model.
(3) **Translate**: the weight matrix $\mathbf{W}^{(i)}$ is treated as a transmission matrix. Therefore it can be "translated" into a photonic device with the help of adjoint optimization. Note that other types of implementations (such as MZI mesh) can also be used: so long as they produce the same transmission matrix, the outcome should remain the same.
(4) **Assemble**: the optimized linear blocks are concatenated with the designed nonlinear activation units.
(5) **Inference**: in order to test its performance, we apply full-wave FDFD simulations to obtain the electric field distributions for different input data.

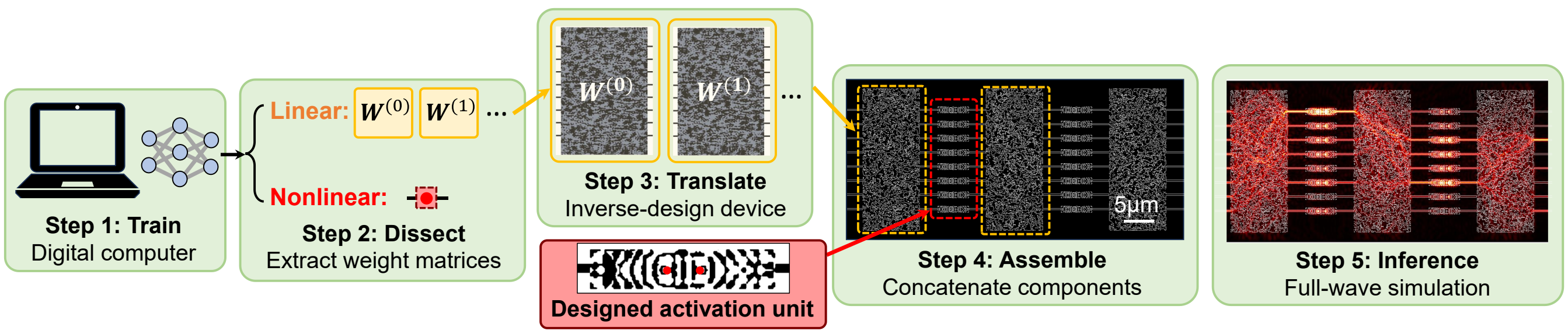

Supplementary Figure 14. **Physics-aware training procedure.** The ONN is first trained on a digital computer. The weight matrices are translated into linear blocks via adjoint optimization. After that, all the linear blocks are concatenated with the nonlinear activation units to form the ONN, which will be tested using full-wave simulations.

In the main text, for the nonlinear classification task the presented results are obtained via full-wave simulation of the entire device. On the other hand, for the reinforcement learning task, the simulation domain is too large, making it expensive to simulate the entire device. As an alternative, we do a domain decomposition and simulate the output of each block individually. The output is then fed into the subsequent block as the input. Finally the $|\mathbf{E}|$ field distributions of different blocks are concatenated. The $|\mathbf{E}|^2$ distributions shown in main text Fig. 3 are obtained in this manner. Such alternative can be justified due to the fact that all our designed linear blocks show negligible reflection. Under such a condition, it is valid to simulate the light propagation in a section-by-section manner, which is a widely-accepted approach for designing diffractive neural networks [46, 47].

SUPPLEMENTARY NOTE 8. NONLINEAR REGRESSION TASK

In this part, we display a nonlinear regression example. Specifically, we try to reproduce $y = \sin(2x)$ with the help of our proposed nonlinear activation unit. As shown in Supplementary Fig. 15(a), the ONN's structure contains two hidden layers, each containing 8 neurons. The input x is a scalar, satisfying $x \in [0, \pi]$. At the output ports, photodetectors are applied, which are modeled as $|\cdot|^2$ in our simulation. Considering that the output $y = \sin(2x)$ can be negative, differential readout is applied, so that the final result is produced by subtracting the total intensity obtained by two groups of detectors. In Supplementary Fig. 15(b), we have displayed the transmission matrix of the

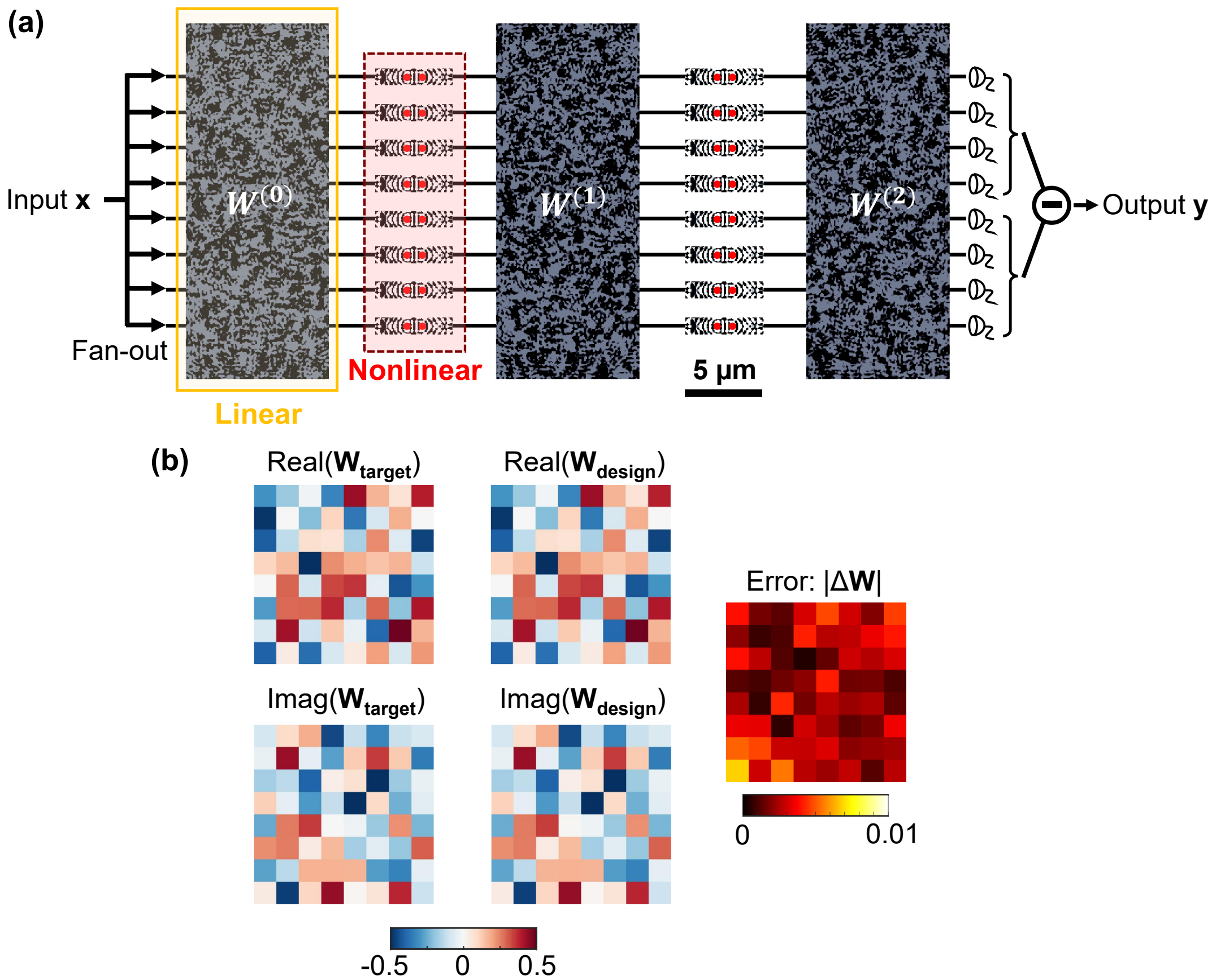

Supplementary Figure 15. **Nonlinear regression task.** (a) ONN architecture for nonlinear regression. The input $x \in [0, \pi]$ is duplicated before sent into input ports. Two hidden layers are included. At the output ports, differential readout is applied to support negative outputs. (b) Transmission matrix design result for $\mathbf{W}^{(0)}$. Through comparison it can be concluded that the designed linear block can reproduce the desired transmission matrix with high fidelity.

first linear block, which corresponds to the matrix $\mathbf{W}^{(0)}$ in Supplementary Fig. 15(a). The unitary matrix obtained from physics-aware training is denoted as $\mathbf{W}_{\text{target}}$, while the transmission matrix of the adjoint-optimized block is denoted as $\mathbf{W}_{\text{design}}$. Both the real parts and the imaginary parts of these two matrices match pretty well. The error $|\Delta\mathbf{W}| = |\mathbf{W}_{\text{design}} - \mathbf{W}_{\text{target}}|$ is also visualized, whose matrix elements are all well below 0.01. Therefore we can safely conclude that through adjoint optimization, the linear blocks can reproduce the desired linear transformation with high fidelity.

The nonlinear regression results are displayed in Supplementary Fig. 16. The gray curve visualizes the ground truth $y = \sin(2x)$. The blue line corresponds to the results obtained through linear regression, which fails to capture the oscillatory behavior. On the other hand, with the quantum-enhanced activation unit, our nonlinear model can fit the $y = \sin(2x)$ curve perfectly (red curve). To verify that the physics-aware training leads to accurate result, we also carry out full-wave FDFD simulations for 16 data points, which produce consistent results. The electric field distributions ($|E_z|$) for 4 data points are also visualized in Supplementary Fig. 16. It can be observed that for different input intensities, the nonlinear activation units can steer the optical fields to different output ports, which then leads to different output results from the differential readout. While the nonlinear regression task is simple, such behavior (re-distribution of energy among different output ports when the input intensity increases) is impossible

to achieve using a linear ONN, in which the output amplitude scales linearly with the input amplitude. Therefore the input-output relation is quadratic (considering the square-law detection), which cannot produce the oscillatory behavior of $y = \sin(2x)$.

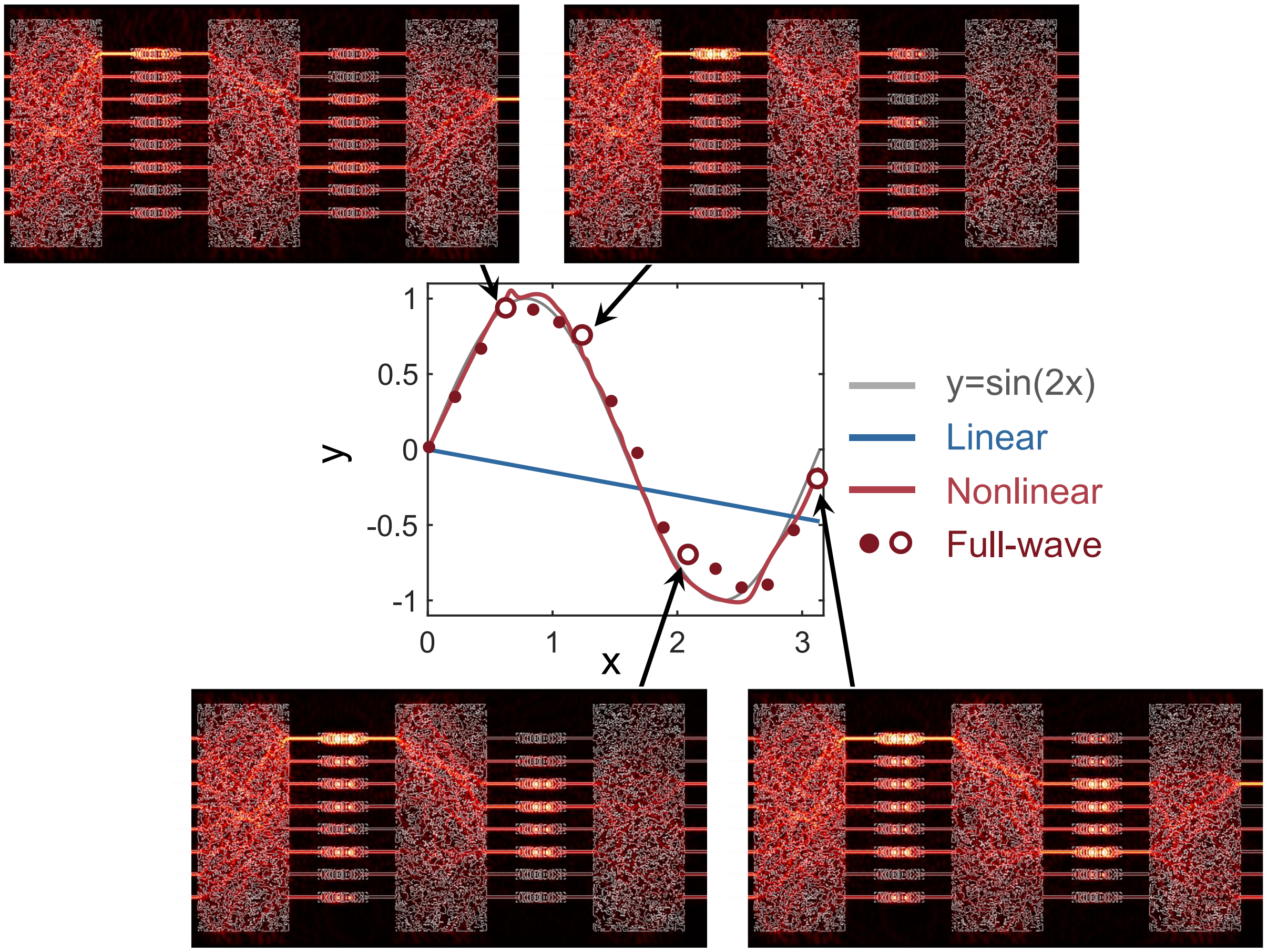

Supplementary Figure 16. **Results for the nonlinear regression task.** The red curve indicates the curve fitting result of our trained nonlinear ONN, which reproduces the ground truth $y = \sin(2x)$ (gray curve) very well. A linear model, on the other hand, fails completely, as shown by the blue curve. The electric field distributions $|E_z|$ of four different data points are visualized.

SUPPLEMENTARY NOTE 9. REINFORCEMENT LEARNING TASKS: DETAILS

In this part we discuss the details related to the training & testing of reinforcement learning tasks. While the tasks we choose seem simple, both of them reveal clear performance gap between nonlinear ONN and linear ONN. This justifies the value of nonlinearity, since for complex tasks a nonlinear mapping is always beneficial. The Atari Pong results reported here are obtained using 2D FDFD, because a 3D verification for this case would be prohibitively expensive.

A. Atari Pong

General setup: the Atari Pong environment used to test our agent is provided by the Gymnasium library [48] (version 1.1.1). Specifically, the game environment used is `ALE/Pong-ram-v5`. The observation type is set as `obs_type = "ram"` (instead of using `obs_type = "rgb"`), which means that for each frame the observation is 128-byte RAM, not the raw image. To obtain the input feature vector for our optical agent, we manually extract the (x, y) coordinates of the player (denoted as $\mathbf{r}_1$, obtained from `ram[46], ram[51]`), the in-game AI (denoted as $\mathbf{r}_2$, obtained from `ram[45], ram[50]`), and the ball (denoted as $\mathbf{r}_b$, obtained from `ram[49], ram[54]`). We also calculate the ball's velocity $\mathbf{v}_b = (v_x, v_y)$ by subtracting the ball's positions between two neighboring frames. Note that the positions are re-scaled, divided by `POSITION_SCALE = 210`; the velocities are also re-scaled, divided by `VELOCITY_SCALE = 8`. Each frame corresponds to a 8-dim feature vector, which concatenates the scaled $\mathbf{r}_1, \mathbf{r}_2, \mathbf{r}_b, \mathbf{v}_b$. Considering that historical information would be helpful for the agent, we concatenate the feature vectors obtained from the previous F frames, which is a standard approach for tackling these Atari games [49]. This is achieved by using the `VecFrameStack()` function. The final input vector has a dimension of $8F$.

The optical agent can be viewed as a policy network, whose structure mimics a standard MLP. Specifically, the network contains L hidden layers (each containing H neurons) as well as an output layer. The input vector is first casted to `complex128` before sent into the neural network. Each layer consists of an isometric linear transformation and the proposed nonlinear activation function. Finally, the output vector is divided into six sections. For each section, the detected amplitudes are summed up to obtain the logits. `softmax()` is applied to the logits to obtain a 6-dim probability distribution, corresponding to the six discrete actions. During the training stage, the actual action is sampled randomly based on the probability distribution, while during inference the agent always takes the action with the highest probability. The feature construction and optical-agent interface are summarized in Supplementary Fig. 17.

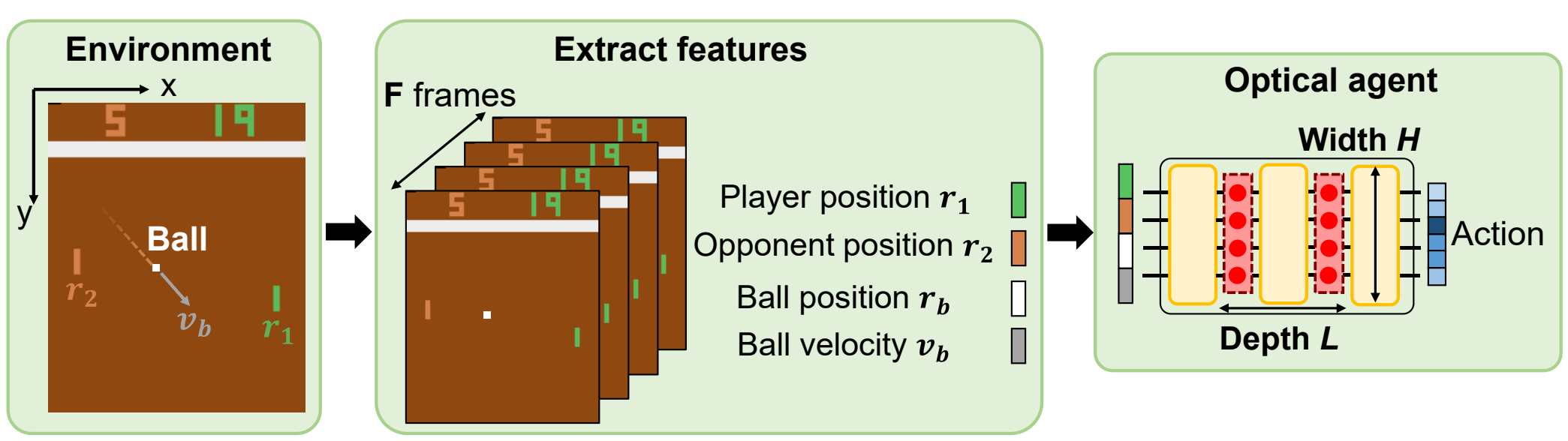

Supplementary Figure 17. **Reinforcement learning setup for Atari Pong.** Hand-crafted features (including positions $\mathbf{r}_1$, $\mathbf{r}_2$, $\mathbf{r}_b$, and velocity $\mathbf{v}_b$) are extracted from the environment. The input vector is constructed by concatenating the feature vectors obtained from F historical frames.

In order to obtain the results displayed in main text Fig. 3(d)(e), we change the model size by sweeping over F, H and L parameters. Specifically, we choose the number of frames $F \in \{1, 2, 3, 4\}$, the network width $H \in \{18, 30, 42, 54\}$, and the network depth $L \in \{2, 3, 4, 5\}$. Note that we add another restriction $H > 8F$, because for an isometric linear transformation, the dimension of the input vector should be smaller than that of the output vector. This parameter sweeping provides us with 52 different sets of parameter combinations. For each combination we train two models, one is linear model (with the activation function switched off), the other is nonlinear model (utilizing the proposed quantum-enhanced activation).

Training details: in order to train our optical agent, we use the proximal policy optimization (PPO) algorithm [50], provided by `stable-baselines3` [51]. In order to accelerate the training process, we run multiple environments (setting `n_env=8`) in parallel with the help of `SubprocVecEnv`. PPO optimizes a composite objective consisting of a

clipped policy-gradient surrogate L_{clip}, a value-function regression loss L_V, and an entropy bonus $\mathbb{E}_t[\mathcal{H}]$. Accordingly, `stable-baselines3` minimizes a loss function which consists a weighted sum of these three terms:

$$\mathcal{L} = -L_{\text{clip}} + \texttt{vf\_coef} \cdot L_V - \texttt{ent\_coef} \cdot \mathbb{E}_t[\mathcal{H}]\,. \tag{49}$$

In our experiments, we choose `vf_coef = 0.5` and linearly change the entropy coefficient from `ent_coef = 1e-2` to `1e-3` during the training process. Details of other PPO parameter settings are chosen as follows:
(1) the length of each rollout is set as `n_steps = 2048`;
(2) batch size is fixed as 512;
(3) each mini-batch is used to update the gradient descent for `n_epochs = 10` times;
(4) when calculating the return, the discount factor is fixed as $\gamma = 0.99$;
(5) we use the default optimizer, which is Adam. The learning rate is fixed as 2×10^{-4}.
The Atari game environment can only be simulated on CPU. Running the optical agent on a GPU leads to frequent data transfer between CPU and GPU, which, in our case, slows down the training process since the optical agent is just a small MLP. Therefore, the training runs purely on CPU. All ONNs are trained using 5×10^7 steps. During the training stage, we evaluate the performance every 20000 steps. Specifically, the trained optical agent plays against the in-game AI for 30 episodes, and the mean/std reward is recorded. After the training stage, only the model with the best mean reward will be saved and tested.

B. HalfCheetah

General setup: we evaluate our method on the `HalfCheetah-v5` benchmark provided by the Gymnasium library [48]. The environment simulates a 2D cheetah-like robot using the MuJoCo physics engine. The agent controls the robot by applying torques to six hinge joints, resulting in a 6-dimensional continuous action space within the range $[-1, 1]$. Each episode lasts for 1000 steps. The raw observation is a 17-dimensional vector consisting of 8 positional values followed by 9 velocity values. To ensure numerical stability, we normalize the raw observation $\mathbf{o}_t$ by dividing each dimension by a fixed scaling constant. This normalization is implemented via a custom `ObservationWrapper` and is applied consistently across all training and testing phases.

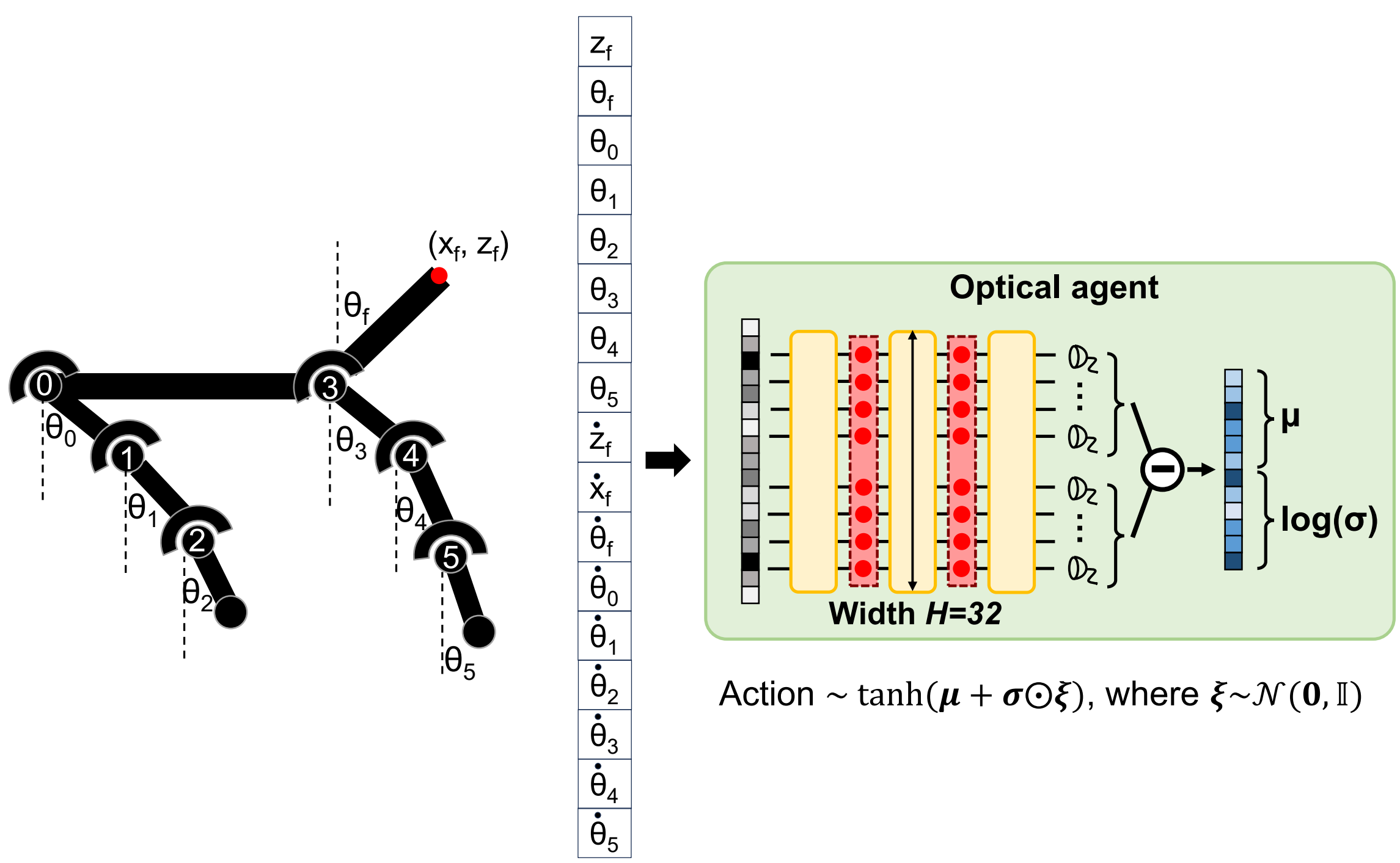

Supplementary Figure 18. **Reinforcement learning setup for HalfCheetah control task.** The 17-dimensional input vector is shown explicitly. The variables with dots indicate time derivatives (velocities or angular velocities). The ONN outputs a 24-dimensional intensity vector, which is then converted into 12 signed values using balanced detection scheme. These values are split to parameterize a Gaussian distribution ($\boldsymbol{\mu}$ and $\log \boldsymbol{\sigma}$), from which the final action (six torques) is sampled.

The optical agent is implemented as a policy network, whose structure mimics a standard MLP (see Supplementary Fig. 18). Specifically, the policy network (actor) consists of $L = 2$ hidden layers (each containing $H = 32$ neurons) and an output layer. The 17-dimensional input vector is first casted to `complex128` before sent into the neural network. Each layer consists of an isometric linear transformation and the proposed nonlinear activation function. Finally, to support the continuous control required by HalfCheetah, we implement a balanced detection mechanism. The ONN turns the input features into a 24-dimensional ($4 \times$ action_dim) output vector. These outputs are detected as non-negative intensities and split into two equal halves. The element-wise difference between these two halves leads to 12 signed values. The first six values serve as the mean $\boldsymbol{\mu}$, while the remaining six values serve as the log-standard deviation $\log \boldsymbol{\sigma}$ for a squashed Gaussian distribution. During the training stage, the continuous action is sampled randomly based on this squashed Gaussian distribution, while during inference the agent always takes the action based on the mean value.

Training & evaluation details: for the comparative experiments presented in the main text, we train two ONNs: a linear model (where the optical activation function is disabled) and the proposed nonlinear model (utilizing the quantum-enhanced activation). Both models share identical hyperparameters and training configurations, ensuring that the performance gap is caused by the nonlinearity. Training is performed using the soft actor-critic algorithm (SAC) [52], provided by `stable-baselines3` [51]. We run multiple environments (`n_env=8`) with the help of `SubprocVecEnv`. No temporal stacking is involved for the HalfCheetah task. Key hyperparameters are summarized as follows:
(1) batch size is fixed as 256;
(2) the size of the replay buffer is `buffer_size = 1000000`;
(3) the discount factor is $\gamma = 0.99$;
(4) the soft update coefficient is $\tau = 0.005$;
(5) we use the default optimizer, which is Adam. The learning rate is fixed as 3×10^{-4};
All ONNs are trained using 4×10^6 steps. We run training on CPU, which is sufficient for the small optical MLPs used here. During training, we evaluate every 10^4 steps using `EvalCallback`, running 20 evaluation episodes. The best-performing checkpoint is saved. During testing, the policy is evaluated for 10 episodes. To generate the velocity curves shown in main text Fig. 3(g), we record the horizontal position $x(t)$ (obtained from `qpos[0]`) at every timestep, and compute the forward velocity by discrete differencing

$$v_x(t) = \frac{x(t) - x(t-k)}{k}, \tag{50}$$

where $k = 10$ is a sliding-window size. This windowed estimate helps suppress high-frequency fluctuations in the trajectory. We then plot $v_x(t)$ averaged over 10 episodes, with shaded areas indicating the standard deviation.

SUPPLEMENTARY NOTE 10. THEORY: QUANTIFYING EXPRESSIVE POWER

In this part we introduce details related to quantifying the expressive power of any given activation function, regardless of whether it's digital or optical. We apply the method proposed in [31, 32], which is well-established in the machine learning theory community. Specifically, consider a trajectory of data points (can be understood as a closed curve inside high-dimensional feature space) that's been propagated layer-by-layer inside a neural network. The conclusion obtained by [32] is summarized here. Suppose the network is wide enough. For each layer, the trajectory's total length L increases by a factor of χ_1, while the averaged local curvature κ changes as

$$\kappa^2 \to \frac{\kappa^2}{\chi_1} + \frac{3\chi_2}{\chi_1^2}, \tag{51}$$

where χ_1 can be calculated based on the 1st-order derivative of the activation function, while χ_2 can be calculated based on its 2nd-order derivative. The total curvature K, which serves as a quantitative measure of the trajectory's complexity, is proportional to $\kappa^2 \cdot L^2$ approximately. Therefore, after each layer, the increase of K follows

$$K^2 \to K^2 + \frac{3\chi_2}{\chi_1} L^2, \tag{52}$$

which only increases when the activation function is nonlinear ($\chi_2 > 0$). Intuitively, this explains that nonlinear activation function can help by injecting curvature into the data manifold. On the other hand, a purely linear transformation does not add complexity to the data manifold, and the total curvature remains constant $K = 2\pi$. We then track how the total curvature of this trajectory evolves during its forward-propagation, since the total curvature K serves as a good metric for evaluating the complexity of a trajectory. Based on the above expressions, it has been proved that with nonlinearity, the total curvature grows exponentially with network depth L, following $K \propto r^L$ [32]. This helps us understand why deep neural networks have to be deep: the expressive power of a neural network grows exponentially with its depth.

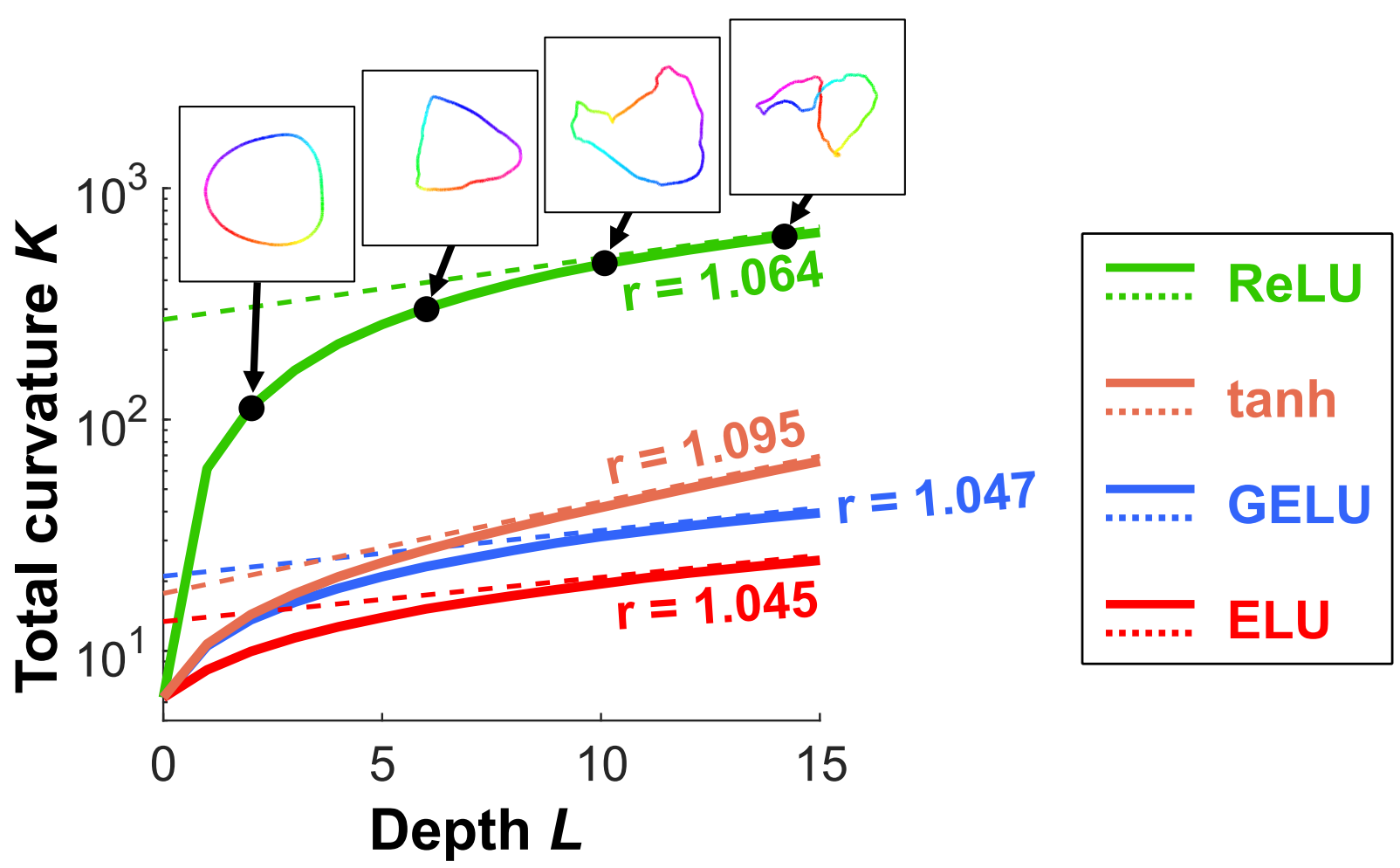

Supplementary Figure 19. **Digital neural network baseline.** The expressive power of four different nonlinear activation functions are evaluated through curve fitting.

Based on the above theory, the expressive power of any given nonlinear activation function can be quantified by ratio r. More specifically, by plugging the nonlinear activation into a neural network, the ratio r can be evaluated numerically. To provide a baseline, we first evaluate r for digital neural network (DNN), focusing on four popular activation functions: ReLU, tanh, GELU and ELU. For each activation function, we construct a simple MLP with $L = 15$ layers. Each hidden layer contains $N_{\text{dim}} = 512$ neurons. The input data points form a circular trajectory, parameterized by $\theta \in [0, 2\pi]$:

$$\mathbf{x}(\theta) = \sqrt{N} \cdot (\mathbf{u}\cos(\theta) + \mathbf{v}\sin(\theta)), \tag{53}$$

where $\mathbf{u}$ and $\mathbf{v}$ are two random unit vectors which form an orthonormal basis for a 2D subspace. The trajectory contains 10000 sampling points, distributed uniformly on $\theta \in [0, 2\pi]$. We note that generally speaking, the growth

factor r depends not only on the activation function but also on the scale of the input data. In the digital baseline considered here this scale is fixed: the input trajectory is normalized by the $\sqrt{N}$ factor above, so that r reduces to the single representative value reported below. We track the evolution of such a trajectory during the forward-propagation process. The results are shown in Supplementary Fig. 19. Four insets are included, which visualize the curves in a 2D subspace (dimension reduction done by PCA). Each data point corresponds to the average value obtained from 10 Monte Carlo simulations. Finally, by doing a curve fitting $K \propto r^L$ using the data obtained from the last five NN layers, the value of ratio r can be extracted. The fitted curves are displayed in Supplementary Fig. 19 using dashed lines. We arrive at the conclusion that, for a DNN, the data complexity increases exponentially with the number of layers L. Specifically, when using ELU, the data complexity increases by 4.5% per layer. This value is used as the digital baseline when we evaluate the intensity requirement for optical nonlinearities.

SUPPLEMENTARY NOTE 11. EXPRESSIVE POWER OF OPTICAL NONLINEARITIES

In this part we focus on understanding the expressive power of different optical nonlinear activations. We first introduce two conventional nonlinearities. Then we apply the proposed theoretical framework to quantify the expressive power of these activation functions, and compare with our proposed quantum-enhanced activation unit.

A. Optical nonlinearity of conventional materials

In this part we introduce two different baselines, for realizing optical nonlinear activation function using conventional materials. The system we consider consists of one input port and one output port. At the input port, the electric field amplitude is denoted as x, while the amplitude at the output port is denoted as y.

1. *Kerr effect of silicon*

Consider a silicon waveguide of length ΔL. The phase difference induced by Kerr nonlinearity can be calculated as $\Delta\phi = n_2 I \cdot k\Delta L$, where $I = \frac{n_0}{2\eta_0}|x|^2$ denotes the light intensity, $k = \frac{2\pi n_0}{\lambda_0}$ denotes the wavenumber inside silicon. Here the wavelength is fixed as $\lambda_0 = 1.5\ \mu$m. Based on existing experimental results [18, 53, 54], the value of n_2 is chosen to be 5×10^{-18} m^2/W. The refractive index $n_0 \approx 3.5$. The input-output relationship of such a nonlinear activation unit can be formulated as

$$y = x \cdot \exp(j\Delta\phi), \tag{54}$$

where the phase difference $\Delta\phi$ depends on input x. The phase difference $n_0 k\Delta L$ has been ignored since it does not depend on intensity, thus providing no nonlinear effect.

2. *Saturable absorption based on graphene*

Consider a saturable absorber, whose absorption coefficient depends on light intensity:

$$\alpha = \frac{\alpha_0}{1 + I/I_{\text{sat}}}, \tag{55}$$

where I_{sat} denotes the saturation intensity. Inside such a material, the evolution of light intensity follows

$$\frac{dI}{dz} = -\alpha(I) \cdot I = -\frac{\alpha_0 I}{1 + I/I_{\text{sat}}}, \tag{56}$$

which can be solved numerically to find the input-output relationship. In this paper we focus on graphene, with parameters $\alpha_0 = 6.8 \times 10^7$ m^{-1}. This coefficient is estimated based on experimental data: each graphene layer (0.34 nm thickness) leads to 2.3% absorption [55]. The saturation intensity is estimated as $I_{\text{sat}} = 6.1 \times 10^9$ W/m^2 [21]. It can be seen that for these conventional optical nonlinearities, the light intensity that's required to observe nonlinear effect is very strong.

B. Quantifying the expressive power

In this part we aim to evaluate the expressive power for optical nonlinearities, by applying the same theoretical framework we used to understand digital neural networks. The procedure has been summarized as 3 steps, shown in Supplementary Fig. 20. For any nonlinear physical system, the first step involves obtaining its input-output relationship (shown as the blue curve). This curve is then interpreted as the nonlinear activation function, and is used to construct a deep neural network in the second step. Finally, the third step involves sending a circular data trajectory into the constructed NN. We then track how its total curvature K increases, and extract the ratio r through $K \propto r^L$ curve fitting, which quantifies the expressive power of this activation function. For any given optical nonlinearity, we apply the above 3-step procedure, then require that the corresponding activation function to have the same level of expressive power as the digital baseline. Specifically, here we require that ratio r should be larger than $r_{\text{min}} = 1.045$, which corresponds to the expressivity of ELU activation function, evaluated in the previous part.

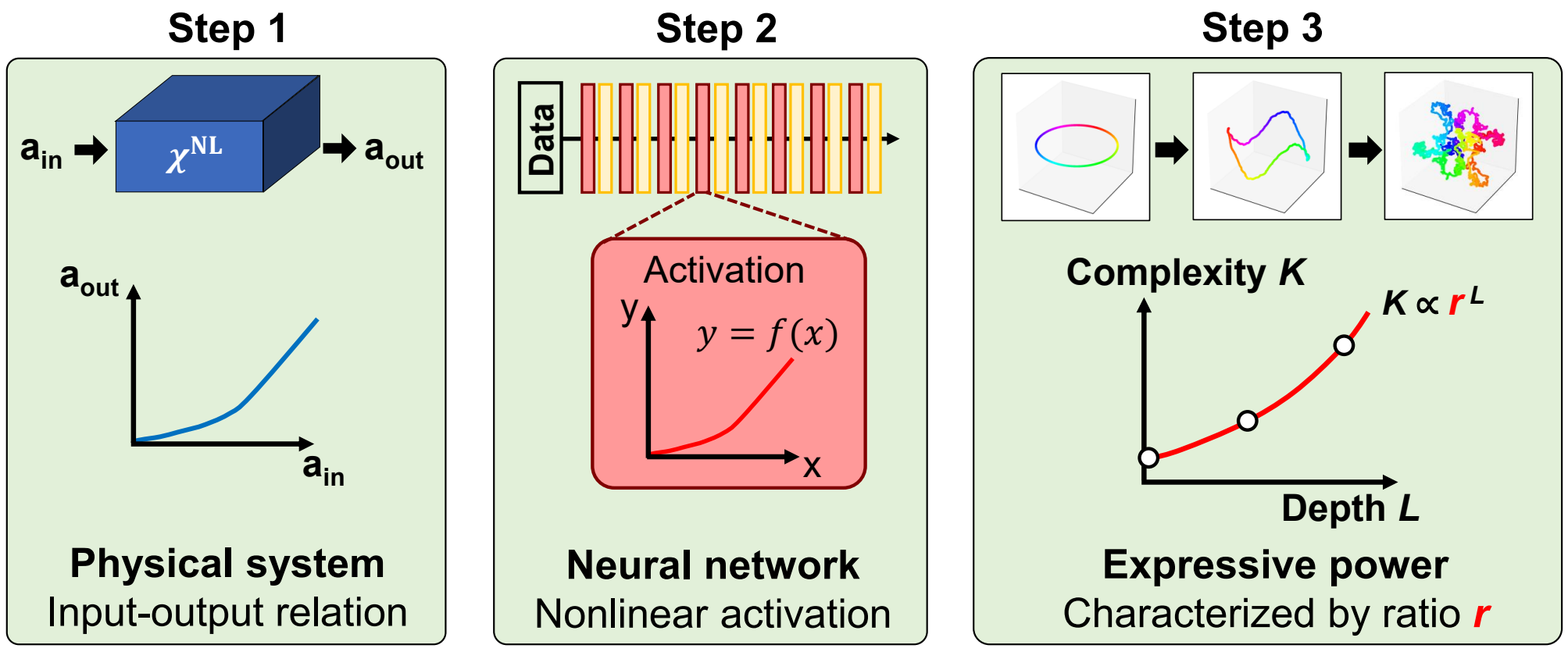

Supplementary Figure 20. **Evaluating the expressive power of a physical system.** The 3-step procedure is general and can be applied to any nonlinear system.

Obviously, r depends on the input light intensity I_0: very weak light leads to a linear mapping with $r = 1$, thus its expressive power does not grow with depth. The above $r > r_{\min}$ requirement posts constraint on the minimum light intensity. To obtain this intensity constraint, we have calculated the ratio r for the two classical nonlinearities. The results are shown in Supplementary Fig. 21. We sweep over two parameters, namely the length/thickness ΔL of the activation unit, and the input light intensity I_0. The $r = r_{\min}$ threshold has also been displayed with red dashed

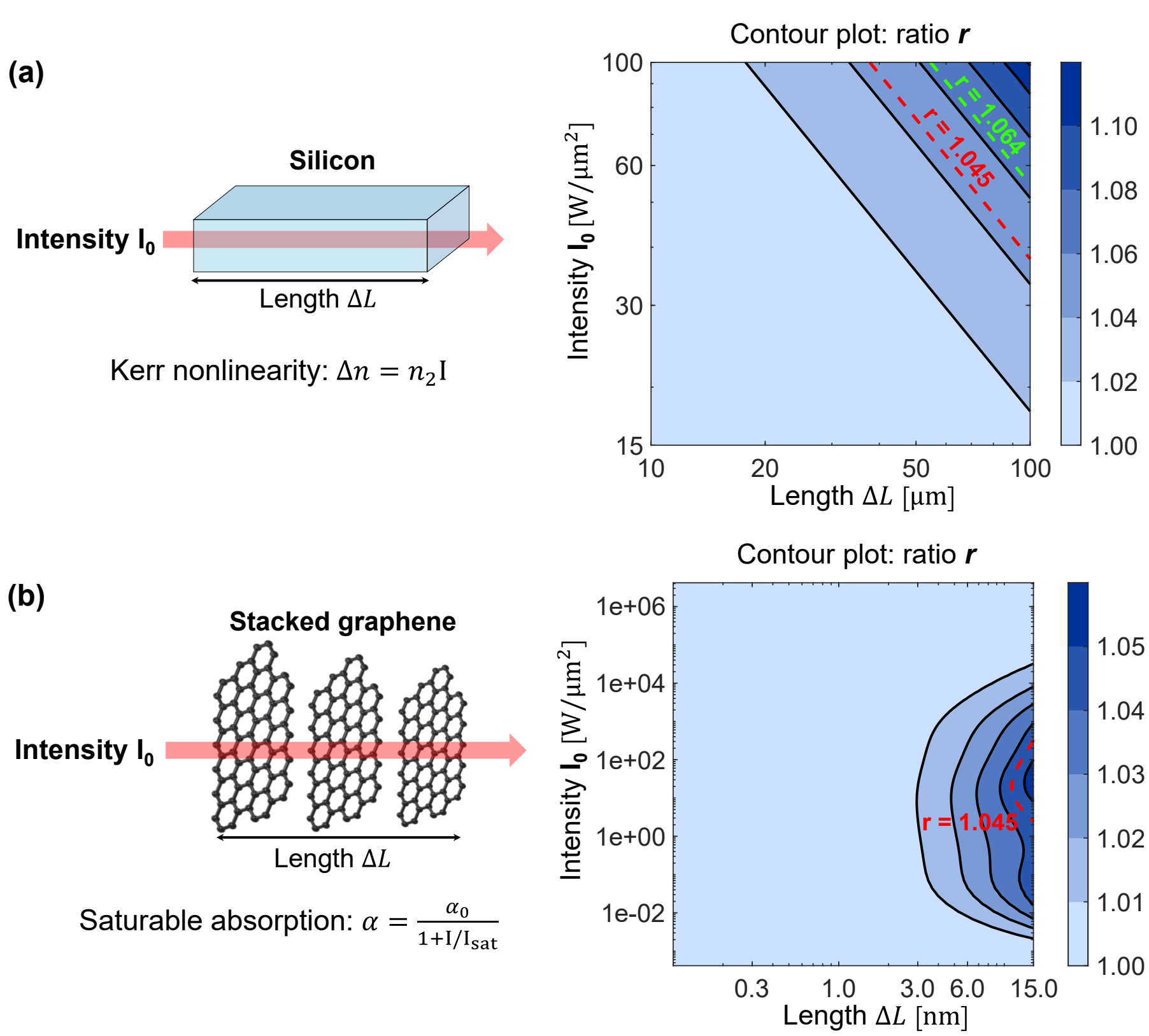

Supplementary Figure 21. **Expressive power of classical nonlinearities.** (a) Silicon waveguide with Kerr nonlinearity. (b) Stacked graphene layers as saturable absorbers. Here we do parameter sweeping w.r.t. length ΔL and light intensity I_0. For an optical nonlinearity, the ratio r has to exceed $r_{\min} = 1.045$ (shown with red dashed contours) in order to be as powerful as its digital counterpart.

lines in the contour plots. The minimum light intensity that's required to reach $r = r_{\min}$ threshold can be extracted directly from these contour plots.

C. Comparison with quantum-enhanced activation

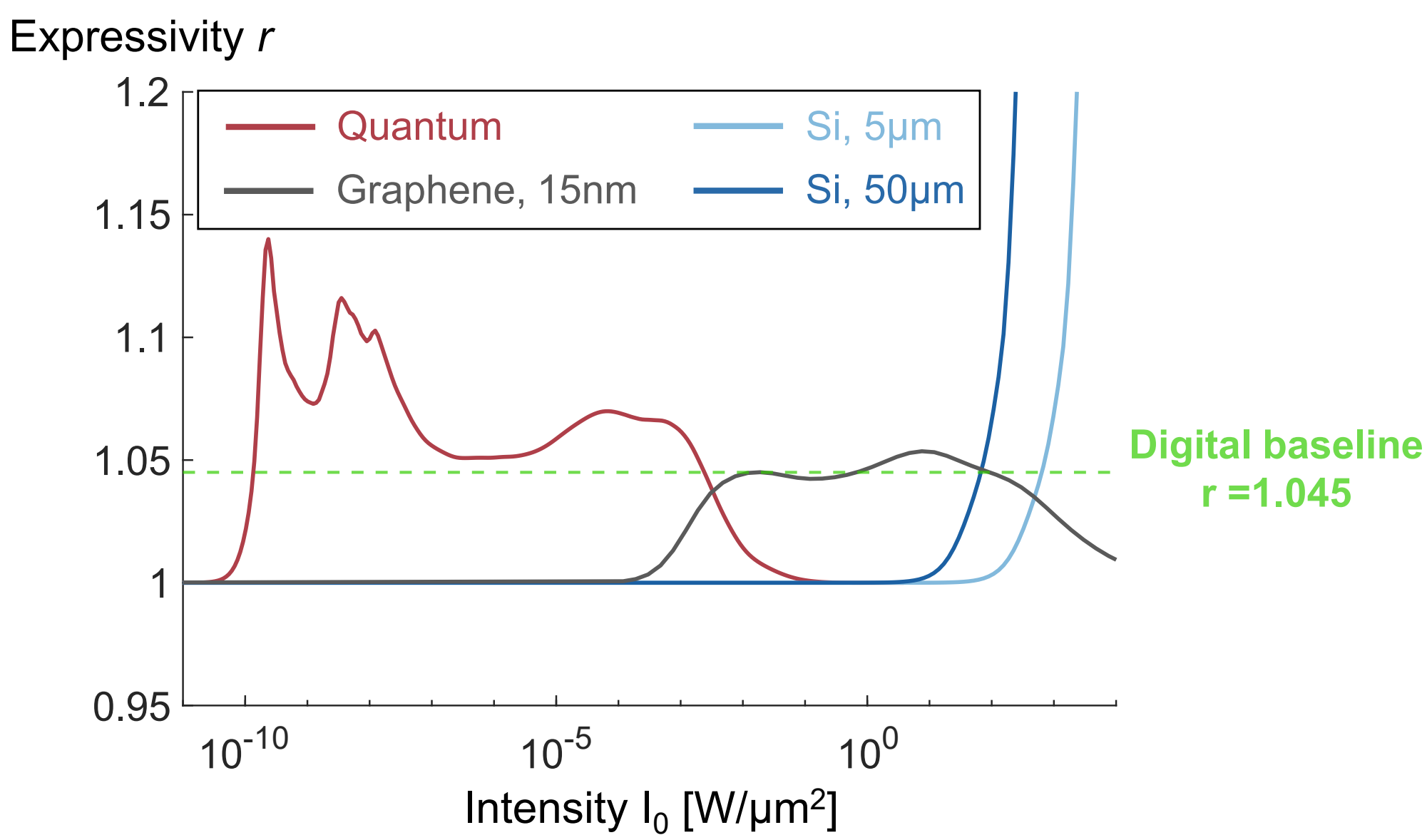

Supplementary Figure 22. **Comparing the expressive power.** For each optical nonlinearity, we visualize its corresponding ratio r (which measures the expressive power) as a function of light intensity I_0. The proposed activation unit can provide enough nonlinearity at very low intensity.

To compare the expressive power of different types of nonlinearities in a more straight-forward manner, here we summarize the relationship between expressive power and light intensity in Supplementary Fig. 22 . The two blue curves correspond to silicon waveguide, with length $\Delta L \in \{5\ \mu\text{m}, 50\ \mu\text{m}\}$. Even for a long waveguide with $\Delta L = 50\ \mu$m, a very high intensity of 72.6 W/μm^2 is required before the Kerr-based nonlinearity becomes useful. The gray dashed line corresponds to stacked graphene whose total thickness is $\Delta L = 15$ nm (this value is chosen such that the intensity does not decay below $1/e$, while keeping a high expressive power). Its threshold can be estimated as 0.02 W/μm^2, which is lower than that of silicon. On the other hand, the proposed quantum nonlinearity, displayed with the red curve, shows strong nonlinearity when the input intensity is as low as 0.24 nW/μm^2. The above intensity restrictions are used when estimating the nonlinearity-limited optical power of optical LLMs.

SUPPLEMENTARY NOTE 12. POWER REQUIREMENTS OF OPTICAL LLMS

In this part we aim to estimate the nonlinearity-limited optical power of running popular LLMs with optical nonlinearities. Our calculation is based on the minimum light intensity constraint, obtained in the previous section.

A. Architecture of modern LLMs

The Transformer architecture was proposed in [56] and has now become the backbone of most popular LLMs. Many modern LLMs can be understood as stacked Transformer blocks. Suppose the input sequence contains L_{seq} tokens (often referred to as the context length). After going through the embedding layer, the input matrix $\mathbf{X} \in \mathbb{R}^{L_{\text{seq}} \times d_{\text{model}}}$, where d_{model} denotes the dimension of embedding. The input is then multiplied with trained weight matrices to obtain the query matrix $\mathbf{Q}$, the key matrix $\mathbf{K}$, and the value matrix $\mathbf{V}$:

$$\mathbf{Q} = \mathbf{X}\mathbf{W}_Q, \mathbf{K} = \mathbf{X}\mathbf{W}_K, \mathbf{V} = \mathbf{X}\mathbf{W}_V, \tag{57}$$

where $\mathbf{Q}, \mathbf{K}, \mathbf{V} \in \mathbb{R}^{L_{\text{seq}} \times d_{\text{model}}}$. Based on this architecture, considering that the weight matrices $\mathbf{W}_Q$, $\mathbf{W}_K$ and $\mathbf{W}_V$ are fixed, only $\mathbf{X}$ should be treated as optical input. Therefore when estimating the power consumption of optical LLM, we set the input dimension of each Transformer layer as $L_{\text{seq}} \times d_{\text{model}} \times 3$. The factor of 3 comes from the fact that 3 copies of $\mathbf{X}$ are required to calculate $\mathbf{Q}$, $\mathbf{K}$ and $\mathbf{V}$.

In Supplementary Table 1 we have summarized the architecture of several LLMs [57–64]. We will use these parameters later when evaluating the power consumption. Note that it's still unclear how self-attention and layer normalization can be implemented with optical computing. Therefore our analysis only serves as a crude estimation, by considering only the intensity requirement of nonlinear activation functions.

Name	Parameters	Context length L_{seq}	Embedding d_{model}	Layer L	Reference
GPT-2-Large	774M	1024	1280	36	[57]
GPT-2-XL	1.54B	1024	1600	48	[57]
GPT-3	175B	2048	12 288	96	[58]
Llama1-65B	65B	2048	8192	80	[59]
Llama2-70B	70B	4096	8192	80	[60]
Llama3-405B	405B	8192	16 384	126	[61]
Deepseek-LLM-7B	7B	4096	4096	30	[62]
Deepseek-v2	236B	4096	5120	60	[63]
Deepseek-v3	671B	4096	7168	61	[64]

Supplementary Table 1. **Decoder-only LLM architecture**. The parameters are used to estimate the optical power requirements of optical LLMs.

B. Estimating the nonlinearity-limited optical power

Intuitively, the nonlinearity-limited optical power of an optical LLM can be estimated based on the following formalism:

$$\text{Power} = \text{Minimum intensity} \times \text{Cross section} \times \text{Input dimension} \times \text{Number of layers.} \tag{58}$$

Here we have assumed that all inputs share the same level of light intensity, which is only limited by the strength of nonlinearity. Under such assumption, the optical power P can be calculated using the following equation:

$$P = I_{\min} \cdot A \cdot (3L_{\text{seq}} \cdot d_{\text{model}}) \cdot L, \tag{59}$$

where $I_{\min}$ stands for the minimum light intensity; A denotes the physical cross section for each input dimension (fixed as $A \approx 0.1\ \mu\text{m}^2$ [65]); $3L_{\text{seq}} \cdot d_{\text{model}}$ gives the dimension of input, and L stands for the number of layers contained in the LLM. Based on the above equation, we evaluate the nonlinearity-limited optical power of several open-source LLMs. We focus on the three types of optical nonlinearities mentioned previously, namely, silicon with Kerr nonlinearity (length $\Delta L = 50\ \mu\text{m}$), graphene as saturable absorber (thickness $\Delta L = 15$ nm), and the proposed nonlinear unit based on quantum emitter. The results are displayed in Supplementary Fig. 23, which presents the same results as

Fig. 4(c) in the main text. In Supplementary Fig. 23(a) the nonlinearity-limited optical power of different models are visualized using histogram. Supplementary Fig. 23(b) visualizes the same set of data, while adding a curve fitting which shows that the optical power grows as $P \propto N_{\text{param}}^{0.66}$ empirically, consistent with [66]. For electronic processors, the power consumption typically scales linearly with N_{param}. The nonlinearity-limited optical power therefore scales more favorably with model size, although a comparison at the system level would require accounting for additional overheads.

In conclusion, the proposed architecture based on quantum nonlinearity can run all LLMs with < 1.2 W optical power. This would be 8.3×10^7 times more efficient than graphene, and 3.0×10^{11} times more efficient than silicon. We emphasize that this value is a lower bound set by the nonlinear activation alone. It does not represent system-level power estimation since the power consumption related to fetching data, A/D (D/A) conversion, modulation, and cooling, are not included. We further note that the area $A \approx 0.1$ μm^2 assumed above corresponds to an on-chip waveguide mode. We would like to point out that the matrix sizes of current integrated photonic processors (of order 10^2 ports [67, 68]) remain far below the embedding dimensions of modern LLMs, so this estimate is, in the near term, more naturally realized on free-space platforms, where the per-neuron area is set by the pixel size. This will rescale the power estimation through the prefactor $I_{\min}A$ while leaving the sublinear scaling $N_{\text{param}}^{0.66}$ unchanged.

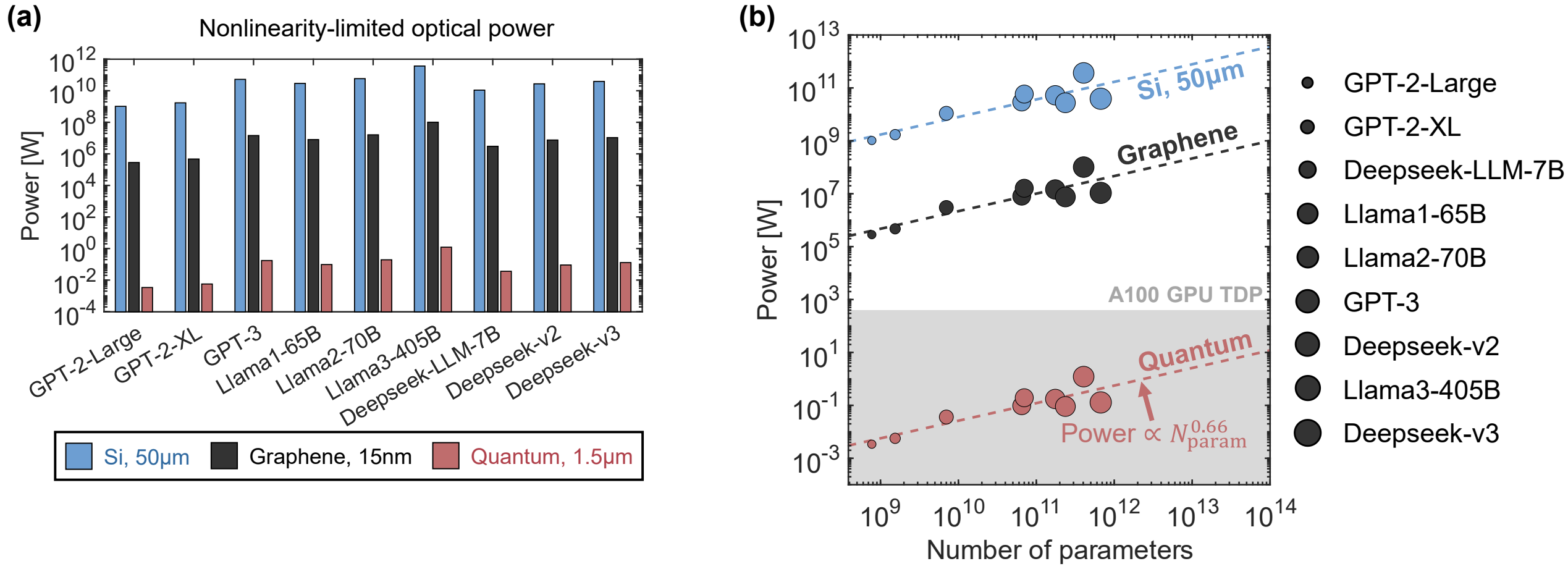

Supplementary Figure 23. **Nonlinearity-limited optical power of LLMs**. (a) Histogram showing the estimated nonlinearity-limited optical power of optical LLMs. Conventional materials demand prohibitive power levels, while the proposed scheme is not restricted by nonlinearity. (b) Estimated nonlinearity-limited optical power versus model size N_{param}. ONNs follow a sublinear scaling $P \propto N_{\text{param}}^{0.66}$.

SUPPLEMENTARY NOTE 13. PURCELL FACTOR OF THE ACTIVATION UNIT

The response bandwidth of the activation unit is limited by the total decay rate of the emitter. As stated in the main text, this speed limit can be alleviated by engineering the photonic environment, which can modify its spontaneous emission rate through the Purcell effect. We therefore quantify the modification of the Purcell factor F_P at the emitter position in the designed activation unit [69].

The spontaneous emission rate is proportional to the imaginary part of the dyadic Green's function evaluated at the emitter position. We place a y-oriented point dipole at the emitter cell and perform two linear frequency-domain (3D FDFD) solves on an identical grid: one filled with the device permittivity ε_{dev} and one filled with bulk-diamond $\varepsilon_{\text{bulk}}$. The Purcell factor is then

$$F_P = \frac{\operatorname{Im} G_{yy}(\mathbf{r}_0, \mathbf{r}_0; \varepsilon_{\text{dev}})}{\operatorname{Im} G_{yy}(\mathbf{r}_0, \mathbf{r}_0; \varepsilon_{\text{bulk}})}, \tag{60}$$

where $\mathbf{r}_0$ is the emitter position and G_{yy} is the yy-component of the dyadic Green's function. The device is a GaP-on-diamond structure (Supplementary Fig. 24): a patterned GaP layer of thickness 200 nm with an in-plane design region of 1.5×0.7 μm, on a 160 nm-thick diamond layer. The SiV^- emitter is located at the center of the design region, 30 nm below the top diamond surface. For the designed activation unit we obtain $F_P \approx 2.74$. We emphasize that this enhancement is emergent since we didn't include the cavity quality factor as a optimization target explicitly. Using a larger photonic crystal cavity should be able to increase F_P considerably, thus the response bandwidth should not be a fundamental limit.

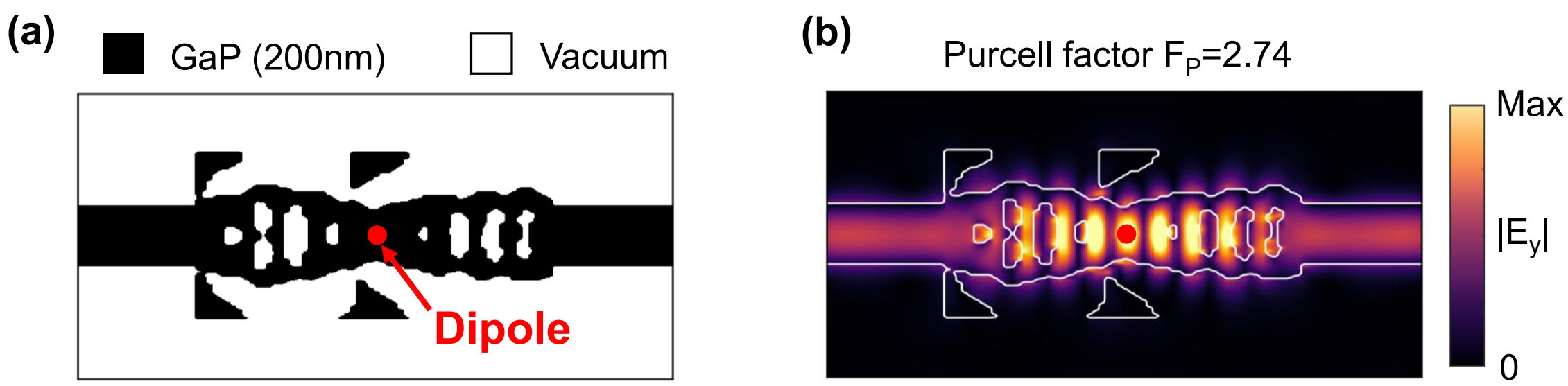

Supplementary Figure 24. Purcell-factor analysis of the designed activation unit. (a) Schematic of the GaP-on-diamond device; the white marker indicates the position of the y-oriented SiV^- emitter, located 30 nm below the diamond surface at the center of the design region. (b) Simulated field distribution through the emitter. The emergent Purcell factor $F_P \approx 2.74$ is obtained as the ratio of the imaginary part of the dyadic Green's function at the emitter position in the device to that in homogeneous diamond.

SUPPLEMENTARY NOTE 14. ROBUSTNESS AGAINST EMITTER NON-IDEALITIES

In this part we characterize how the activation unit performs under two emitter non-idealities, namely the position randomness of the emitter and the spectral disorder. The effect of a finite quantum efficiency has already been presented in Supplementary Note 5. All the designs and analysis presented here are based on the 3D GaP-on-diamond platform. Material parameters, geometry, the FoM, and the optimization hyperparameters remain the same as explained in Supplementary Note 4.

A. Robustness to emitter position randomness

While placement of single SiV^- can be realized by focused ion beam implantation, the position of the obtained emitter is random and often spans a few tens of nm [37, 38]. To assess whether the activation unit tolerates such uncertainty, we randomize the lateral position of the SiV^- emitter over a 50nm $\times$ 60nm region, as shown in Supplementary Fig. 25(a). The emitter depth is fixed as 30 nm. At each optimization iteration, six positions are drawn from this region and the FoM is averaged over the batch, so the inverse design should lead to a structure which is insensitive to the emitter's position.

As shown in Supplementary Fig. 25(b), the optimization converges to FoM = 2.295 (the original design leads to 2.557). We then verify the converged design on 5 different positions drawn from the same region. The resulting transmission curves remain sigmoidal, as shown in Supplementary Fig. 25(c). Across all tested positions, the transmission contrast $|S_{21}|^2$ between the strong- and weak-signal limits remains above 22$\times$, well above the level required by the classification task. Therefore, we conclude that with the help of inverse design, the activation unit can become pretty robust to realistic random placement.

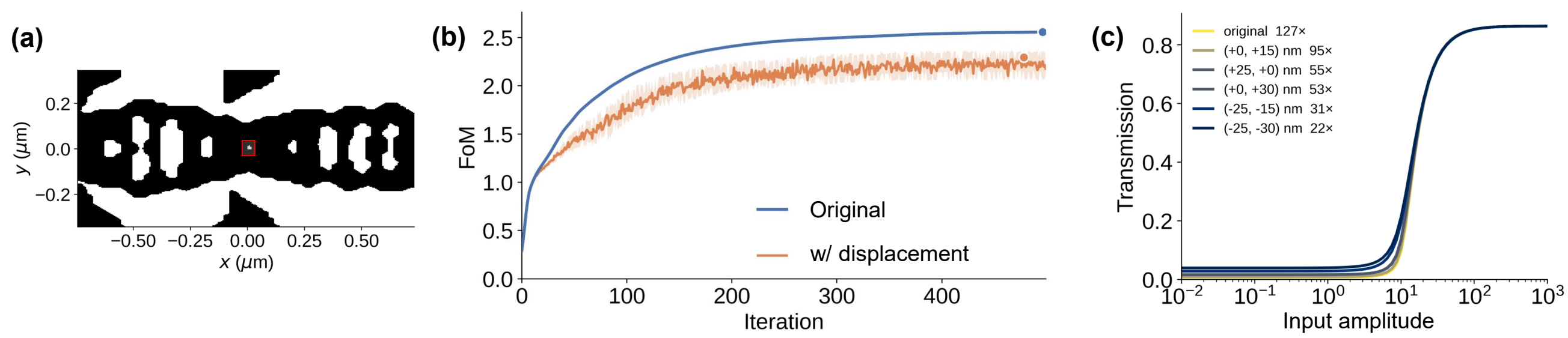

Supplementary Figure 25. **Robustness to emitter position randomness.** (a) Setup: the inverse-designed GaP pattern. The emitter's lateral position is sampled from a 50nm $\times$ 60nm region, marked by the red rectangle. (b) FoM curves: the original training curve (emitter position fixed at the middle) and the position-randomized training curve. The shaded area marks the batch minimum/maximum at each iteration. (c) The obtained transmission curve $|S_{21}|^2$ for several different emitter positions, showing that the activation curve is preserved.

B. Robustness to spectral disorder

Due to inhomogeneous broadening, the transition frequency of SiV^- is not a fixed value. This leads to a detuning Δ between the emitter and the optical signal (see Supplementary Fig. 26(a)). Since the activation relies on the emitter being resonant with the input signal, such detuning weakens the nonlinear response. To understand how inhomogeneity affects the performance, we sample the detuning from Gaussian distribution $\Delta \sim \mathcal{N}(0, \sigma^2)$ with $\sigma/2\pi = 50$ MHz. During the optimization the FoM is averaged over six detunings, similar to the previous part.

As shown in Supplementary Fig. 26(b), the optimization converges to FoM = 2.297. The resulting design, shown in Supplementary Fig. 26(c), is then verified with 5 different detunings [Supplementary Fig. 26(d)]. As can be seen, while the sigmoidal shape is retained, the transmission contrast drops as the detuning goes to 100 MHz. We emphasize that the realistic inhomogeneous linewidth of SiV^- is of order GHz, which is much higher than what we can handle. Spectral disorder therefore remains a limitation. In principle, it can be mitigated by tuning the resonance of each emitter individually, for example via the DC Stark shift [70], as we have noted in the main text.

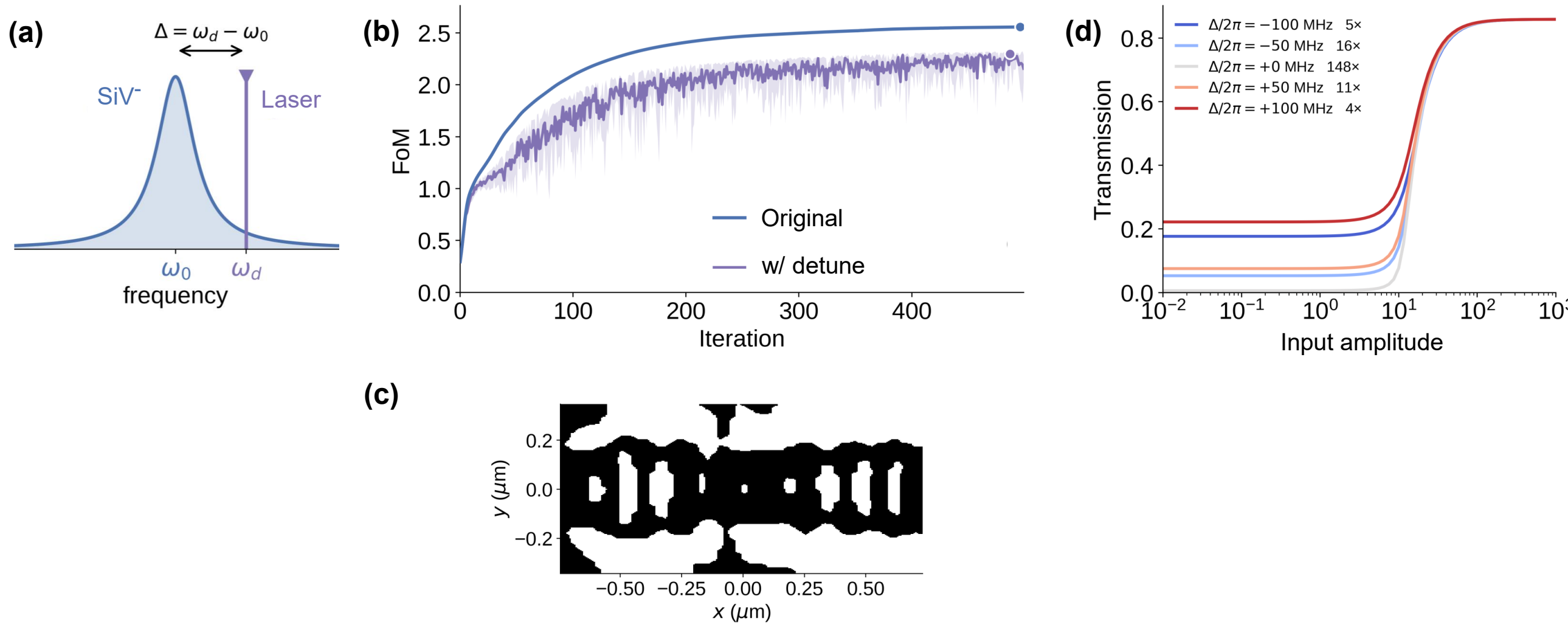

Supplementary Figure 26. **Robustness to spectral disorder.** (a) Setup: schematic of the detuning $\Delta = \omega_d - \omega_0$. (b) FoM curves: the original training curve ($\Delta = 0$) and the detuning-aware training curve. The shaded area marks the batch minimum/maximum at each iteration. (c) The designed pattern which is robust against spectral disorder. (d) The obtained transmission curve $|S_{21}|^2$ for several detunings $\Delta/2\pi = 0, \pm 50, \pm 100$ MHz, showing how the contrast degrades with larger detune.

SUPPLEMENTARY REFERENCES

[1] T. W. Hughes, M. Minkov, I. A. Williamson, and S. Fan, Adjoint method and inverse design for nonlinear nanophotonic devices, ACS Photonics **5**, 4781 (2018).

[2] J. Feldmann, N. Youngblood, C. D. Wright, H. Bhaskaran, and W. H. Pernice, All-optical spiking neurosynaptic networks with self-learning capabilities, Nature **569**, 208 (2019).

[3] T. W. Hughes, I. A. Williamson, M. Minkov, and S. Fan, Wave physics as an analog recurrent neural network, Science advances **5**, eaay6946 (2019).

[4] W. Yu, S. Zheng, Z. Zhao, B. Wang, and W. Zhang, Reconfigurable low-threshold all-optical nonlinear activation functions based on an add-drop silicon microring resonator, IEEE Photonics Journal **14**, 1 (2022).

[5] Y. Shi, J. Ren, G. Chen, W. Liu, C. Jin, X. Guo, Y. Yu, and X. Zhang, Nonlinear germanium-silicon photodiode for activation and monitoring in photonic neuromorphic networks, Nature Communications **13**, 6048 (2022).

[6] B. Wu, H. Li, W. Tong, J. Dong, and X. Zhang, Low-threshold all-optical nonlinear activation function based on a ge/si hybrid structure in a microring resonator, Optical Materials Express **12**, 970 (2022).

[7] C. Chen, Z. Yang, T. Wang, Y. Wang, K. Gao, J. Wu, J. Wang, J. Qiu, and D. Tan, Ultra-broadband all-optical nonlinear activation function enabled by mote2/optical waveguide integrated devices, Nature Communications **15**, 9047 (2024).

[8] Z. Yang, J. He, Z. Yan, Y. Hu, X. Li, N. Dong, and J. Wang, Inverse-designed integrated all-optical nonlinear activators for optical computing, Optics Express **32**, 34001 (2024).

[9] B. Zhao, L. Lin, A. Samuel, and M. Lawrence, High-resolution and ultra-low power nonlinear image processing with passive high-quality factor metasurfaces, arXiv preprint arXiv:2504.02981 (2025).

[10] C. Huang, T. F. De Lima, A. Jha, S. Abbaslou, A. N. Tait, B. J. Shastri, and P. R. Prucnal, Programmable silicon photonic optical thresholder, IEEE Photonics Technology Letters **31**, 1834 (2019).

[11] A. Jha, C. Huang, and P. R. Prucnal, Reconfigurable all-optical nonlinear activation functions for neuromorphic photonics, Optics letters **45**, 4819 (2020).

[12] T. Wu, Y. Li, L. Ge, and L. Feng, Field-programmable photonic nonlinearity, Nature Photonics **19**, 725 (2025).

[13] G. H. Li, R. Sekine, R. Nehra, R. M. Gray, L. Ledezma, Q. Guo, and A. Marandi, All-optical ultrafast relu function for energy-efficient nanophotonic deep learning, Nanophotonics **12**, 847 (2023).

[14] M. M. Pour Fard, I. A. Williamson, M. Edwards, K. Liu, S. Pai, B. Bartlett, M. Minkov, T. W. Hughes, S. Fan, and T.-A. Nguyen, Experimental realization of arbitrary activation functions for optical neural networks, Optics Express **28**, 12138 (2020).

[15] F. Ashtiani, A. J. Geers, and F. Aflatouni, An on-chip photonic deep neural network for image classification, Nature **606**,

501 (2022).
[16] C. Zhong, K. Liao, T. Dai, M. Wei, H. Ma, J. Wu, Z. Zhang, Y. Ye, Y. Luo, Z. Chen, *et al.*, Graphene/silicon heterojunction for reconfigurable phase-relevant activation function in coherent optical neural networks, Nature Communications **14**, 6939 (2023).
[17] Q. Feng, C. B. Uzundal, R. Guo, C. Sanborn, R. Qi, J. Xie, J. Zhang, J. Wu, and F. Wang, Femtojoule optical nonlinearity for deep learning with incoherent illumination, Science Advances **11**, eads4224 (2025).
[18] E. Dulkeith, Y. A. Vlasov, X. Chen, N. C. Panoiu, and R. M. Osgood Jr, Self-phase-modulation in submicron silicon-on-insulator photonic wires, Optics express **14**, 5524 (2006).
[19] N. Kumar, S. Najmaei, Q. Cui, F. Ceballos, P. M. Ajayan, J. Lou, and H. Zhao, Second harmonic microscopy of monolayer mos 2, Physical Review B—Condensed Matter and Materials Physics **87**, 161403 (2013).
[20] B. Liu, K. Liang, Q. Zhou, A. R. Khan, Z. Lu, T. Yildirim, X. Sun, S. Rahman, Y. Liu, Z. Yu, *et al.*, Giant second harmonic generation in two-dimensional tellurene with synthesis and thickness engineering, Applied physics reviews **12** (2025).
[21] Q. Bao, H. Zhang, Y. Wang, Z. Ni, Y. Yan, Z. X. Shen, K. P. Loh, and D. Y. Tang, Atomic-layer graphene as a saturable absorber for ultrafast pulsed lasers, Advanced Functional Materials **19**, 3077 (2009).
[22] Q. Bao, H. Zhang, Z. Ni, Y. Wang, L. Polavarapu, Z. Shen, Q.-H. Xu, D. Tang, and K. P. Loh, Monolayer graphene as a saturable absorber in a mode-locked laser, Nano Research **4**, 297 (2011).
[23] M. R. Lamont, B. Luther-Davies, D.-Y. Choi, S. Madden, and B. J. Eggleton, Supercontinuum generation in dispersion engineered highly nonlinear ($\gamma = 10$ /w/m) as_2s_3 chalcogenide planar waveguide, Optics Express **16**, 14938 (2008).
[24] M. Pu, L. Ottaviano, E. Semenova, and K. Yvind, Efficient frequency comb generation in algaas-on-insulator, Optica **3**, 823 (2016).
[25] C. Wang, X. Xiong, N. Andrade, V. Venkataraman, X.-F. Ren, G.-C. Guo, and M. Lončar, Second harmonic generation in nano-structured thin-film lithium niobate waveguides, Optics Express **25**, 6963 (2017).
[26] G. Kopp and J. L. Lean, A new, lower value of total solar irradiance: Evidence and climate significance, Geophysical Research Letters **38** (2011).
[27] Q. Zhou, S. Gangaraj, M. Zhou, and Z. Yu, Simulating quantum emitters in arbitrary photonic environments using fdtd: beyond the semi-classical regime, arXiv preprint arXiv:2410.16118 (2024).
[28] H. Wang and S. Fan, Lorentz–drude dipoles in the radiative limit and their modeling in finite-difference time-domain methods, Annalen der Physik , e00156 (2025).
[29] J. Nocedal, Numerical optimization (2006).
[30] W. Wirtinger, Zur formalen theorie der funktionen von mehr komplexen veränderlichen, Mathematische Annalen **97**, 357 (1927).
[31] B. Poole, S. Lahiri, M. Raghu, J. Sohl-Dickstein, and S. Ganguli, Exponential expressivity in deep neural networks through transient chaos, Advances in neural information processing systems **29** (2016).
[32] M. Raghu, B. Poole, J. Kleinberg, S. Ganguli, and J. Sohl-Dickstein, On the expressive power of deep neural networks, in *international conference on machine learning* (PMLR, 2017) pp. 2847–2854.
[33] C. Hepp, T. Müller, V. Waselowski, J. N. Becker, B. Pingault, H. Sternschulte, D. Steinmüller-Nethl, A. Gali, J. R. Maze, M. Atatüre, and C. Becher, Electronic structure of the silicon vacancy color center in diamond, Physical Review Letters **112**, 036405 (2014).
[34] S. W. Ding, M. Haas, X. Guo, K. Kuruma, C. Jin, Z. Li, D. D. Awschalom, N. Delegan, F. J. Heremans, A. A. High, and M. Lončar, High-q cavity interface for color centers in thin film diamond, Nat. Commun. **15**, 6358 (2024).
[35] S. Chakravarthi, N. S. Yama, A. Abulnaga, D. Huang, C. Pederson, K. Hestroffer, F. Hatami, N. P. de Leon, and K.-M. C. Fu, Hybrid integration of GaP photonic crystal cavities with silicon-vacancy centers in diamond by stamp-transfer, Nano Lett. **23**, 3708 (2023).
[36] N. S. Yama, C.-C. Wu, F. Hatami, and K.-M. C. Fu, A scalable gallium-phosphide-on-diamond spin-photon interface, arXiv preprint arXiv:2601.04733 (2026).
[37] T. Schröder, M. E. Trusheim, M. Walsh, L. Li, J. Zheng, M. Schukraft, A. Sipahigil, R. E. Evans, D. D. Sukachev, C. T. Nguyen, J. L. Pacheco, R. M. Camacho, E. S. Bielejec, M. D. Lukin, and D. Englund, Scalable focused ion beam creation of nearly lifetime-limited single quantum emitters in diamond nanostructures, Nature Communications **8**, 15376 (2017).
[38] M. Titze, H. Byeon, A. Flores, J. Henshaw, C. T. Harris, A. M. Mounce, and E. S. Bielejec, In situ ion counting for improved implanted ion error rate and silicon vacancy yield uncertainty, Nano Lett. **22**, 3212 (2022).
[39] K. D. Jahnke, A. Sipahigil, J. M. Binder, M. W. Doherty, M. Metsch, L. J. Rogers, N. B. Manson, M. D. Lukin, and F. Jelezko, Electron–phonon processes of the silicon-vacancy centre in diamond, New Journal of Physics **17**, 043011 (2015).
[40] S. Fan, W. Suh, and J. D. Joannopoulos, Temporal coupled-mode theory for the fano resonance in optical resonators, Journal of the Optical Society of America A **20**, 569 (2003).
[41] M. Hammer and O. V. Ivanova, Effective index approximations of photonic crystal slabs: a 2-to-1-d assessment, Optical and Quantum Electronics **41**, 267 (2009).
[42] V. Nikkhah, A. Pirmoradi, F. Ashtiani, B. Edwards, F. Aflatouni, and N. Engheta, Inverse-designed low-index-contrast structures on a silicon photonics platform for vector–matrix multiplication, Nature Photonics **18**, 501 (2024).
[43] M. Bézard, Y. Mindarava, R. Blinder, J.-B. Trebbia, P. Tamarat, F. Jelezko, and B. Lounis, Unveiling the high quantum efficiency of single silicon-vacancy centers through dielectric tuning of their local environment, AVS Quantum Science **6**, 031401 (2024).
[44] Y. LeCun, The mnist database of handwritten digits, http://yann. lecun. com/exdb/mnist/ (1998).
[45] H. Xiao, K. Rasul, and R. Vollgraf, Fashion-mnist: a novel image dataset for benchmarking machine learning algorithms,

arXiv preprint arXiv:1708.07747 (2017).
[46] X. Lin, Y. Rivenson, N. T. Yardimci, M. Veli, Y. Luo, M. Jarrahi, and A. Ozcan, All-optical machine learning using diffractive deep neural networks, Science **361**, 1004 (2018).
[47] Z. Wu, M. Zhou, E. Khoram, B. Liu, and Z. Yu, Neuromorphic metasurface, Photonics Research **8**, 46 (2019).
[48] M. Towers, A. Kwiatkowski, J. Terry, J. U. Balis, G. De Cola, T. Deleu, M. Goulão, A. Kallinteris, M. Krimmel, A. KG, *et al.*, Gymnasium: A standard interface for reinforcement learning environments, arXiv preprint arXiv:2407.17032 (2024).
[49] V. Mnih, K. Kavukcuoglu, D. Silver, A. A. Rusu, J. Veness, M. G. Bellemare, A. Graves, M. Riedmiller, A. K. Fidjeland, G. Ostrovski, *et al.*, Human-level control through deep reinforcement learning, nature **518**, 529 (2015).
[50] J. Schulman, F. Wolski, P. Dhariwal, A. Radford, and O. Klimov, Proximal policy optimization algorithms, arXiv preprint arXiv:1707.06347 (2017).
[51] A. Raffin, A. Hill, A. Gleave, A. Kanervisto, M. Ernestus, and N. Dormann, Stable-baselines3: Reliable reinforcement learning implementations, Journal of machine learning research **22**, 1 (2021).
[52] T. Haarnoja, A. Zhou, P. Abbeel, and S. Levine, Soft actor-critic: Off-policy maximum entropy deep reinforcement learning with a stochastic actor, in *International conference on machine learning* (Pmlr, 2018) pp. 1861–1870.
[53] M. Dinu, F. Quochi, and H. Garcia, Third-order nonlinearities in silicon at telecom wavelengths, Applied physics letters **82**, 2954 (2003).
[54] A. D. Bristow, N. Rotenberg, and H. M. Van Driel, Two-photon absorption and kerr coefficients of silicon for 850–2200nm, Applied physics letters **90** (2007).
[55] K. Y. Lau, X. Liu, and J. Qiu, A comparison for saturable absorbers: Carbon nanotube versus graphene, Advanced Photonics Research **3**, 2200023 (2022).
[56] A. Vaswani, N. Shazeer, N. Parmar, J. Uszkoreit, L. Jones, A. N. Gomez, Ł. Kaiser, and I. Polosukhin, Attention is all you need, Advances in neural information processing systems **30** (2017).
[57] A. Radford, J. Wu, R. Child, D. Luan, D. Amodei, I. Sutskever, *et al.*, Language models are unsupervised multitask learners, OpenAI blog **1**, 9 (2019).
[58] T. Brown, B. Mann, N. Ryder, M. Subbiah, J. D. Kaplan, P. Dhariwal, A. Neelakantan, P. Shyam, G. Sastry, A. Askell, *et al.*, Language models are few-shot learners, Advances in neural information processing systems **33**, 1877 (2020).
[59] H. Touvron, T. Lavril, G. Izacard, X. Martinet, M.-A. Lachaux, T. Lacroix, B. Rozière, N. Goyal, E. Hambro, F. Azhar, *et al.*, Llama: Open and efficient foundation language models, arXiv preprint arXiv:2302.13971 (2023).
[60] H. Touvron, L. Martin, K. Stone, P. Albert, A. Almahairi, Y. Babaei, N. Bashlykov, S. Batra, P. Bhargava, S. Bhosale, *et al.*, Llama 2: Open foundation and fine-tuned chat models, arXiv preprint arXiv:2307.09288 (2023).
[61] A. Dubey, A. Jauhri, A. Pandey, A. Kadian, A. Al-Dahle, A. Letman, A. Mathur, A. Schelten, A. Yang, A. Fan, *et al.*, The llama 3 herd of models, arXiv e-prints , arXiv (2024).
[62] X. Bi, D. Chen, G. Chen, S. Chen, D. Dai, C. Deng, H. Ding, K. Dong, Q. Du, Z. Fu, *et al.*, Deepseek llm: Scaling open-source language models with longtermism, arXiv preprint arXiv:2401.02954 (2024).
[63] A. Liu, B. Feng, B. Wang, B. Wang, B. Liu, C. Zhao, C. Dengr, C. Ruan, D. Dai, D. Guo, *et al.*, Deepseek-v2: A strong, economical, and efficient mixture-of-experts language model, arXiv preprint arXiv:2405.04434 (2024).
[64] A. Liu, B. Feng, B. Xue, B. Wang, B. Wu, C. Lu, C. Zhao, C. Deng, C. Zhang, C. Ruan, *et al.*, Deepseek-v3 technical report, arXiv preprint arXiv:2412.19437 (2024).
[65] Y. Shen, N. C. Harris, S. Skirlo, M. Prabhu, T. Baehr-Jones, M. Hochberg, X. Sun, S. Zhao, H. Larochelle, D. Englund, *et al.*, Deep learning with coherent nanophotonic circuits, Nature photonics **11**, 441 (2017).
[66] M. Anderson, S.-Y. Ma, T. Wang, L. Wright, and P. McMahon, Optical transformers, Transactions on Machine Learning Research (2023).
[67] S. Hua, E. Divita, S. Yu, B. Peng, C. Roques-Carmes, *et al.*, An integrated large-scale photonic accelerator with ultralow latency, Nature **640**, 361 (2025).
[68] S. R. Ahmed, R. Baghdadi, M. Bernadskiy, *et al.*, Universal photonic artificial intelligence acceleration, Nature **640**, 368 (2025).
[69] L. Novotny and B. Hecht, *Principles of nano-optics* (Cambridge University Press, 2012).
[70] A. Laucht, J. Villas-Bôas, S. Stobbe, N. Hauke, F. Hofbauer, G. Böhm, P. Lodahl, M.-C. Amann, M. Kaniber, and J. Finley, Mutual coupling of two semiconductor quantum dots via an optical nanocavity, Physical Review B—Condensed Matter and Materials Physics **82**, 075305 (2010).
